# Pharmacological Mechanisms of Ursolic Acid Derivative Against Prostate Cancer via Regulating Cytoskeletal Homeostasis and Apoptotic Pathways

**DOI:** 10.3390/ph19050726

**Published:** 2026-05-02

**Authors:** Huiyue Shen, Zhaolan Ni, Haibo Guo, Xiaofeng Liu, Yaru Zhao, Xuan He, Yinghan Liu, Yan Zhao, Hongbo Teng

**Affiliations:** 1 College of Chinese Medicinal Materials, Jilin Agricultural University, Changchun 130118, China; 2 People’s Hospital of Ningxia Hui Autonomous, Ningxia Medical University, Yinchuan 750002, China; 3 International Joint Laboratory for Development of Animal and Plant Resources for Food and Medicine, Jilin Agricultural University, Changchun 130118, China

**Keywords:** ursolic acid derivatives, triphenylphosphine compounds, PC3-M cells, cytoskeleton, apoptosis

## Abstract

**Background**: Ursolic acid (UA) is a natural pentacyclic triterpenoid with notable antitumor activity, yet its poor water solubility and insufficient targeting restrict clinical translation. **Methods**: Forty novel ursolic acid-phosphine derivatives bearing seven distinct lipophilic cationic moieties were synthesized via C28 modification and structurally characterized by ^1^H NMR and ^13^C NMR. Their antitumor activities in PC3-M cells were evaluated via in vitro assays. Mechanistic investigations were performed using transcriptomic analysis and Western blot. Molecular docking was performed to predict the binding profile of Compound **25** with FGFR1. In vivo antitumor efficacy and biosafety were assessed in RM-1 xenograft models in C57BL/6 mice. **Results**: Compound **25** (bearing a tris(3,5-dimethylphenyl)phosphine group at the C28 position with an alkyl chain length of five methylene units) exhibited the most potent activity against PC3-M cells, dose-dependently inhibiting proliferation, migration, and invasion and inducing apoptosis. It triggered mitochondrial apoptosis via ROS accumulation and disrupted cytoskeletal homeostasis by suppressing the FGFR1/KRAS/RAC1/PIP4K2 axis. Molecular docking results suggested its strong binding affinity and specificity. In vivo studies confirmed its significant antitumor effect and favorable safety. **Conclusions**: These results highlight the potential of Compound **25** as a promising lead compound and provide valuable insights for further medicinal chemistry optimization and the development of novel anticancer drugs derived from ursolic acid.

## 1. Introduction

Prostate cancer (PCa) is one of the most common malignant tumors in men worldwide [1]. According to data released by the U.S. National Center for Health Statistics (NCHS) [2], a total of 608,371 cancer-related deaths were recorded in the United States in 2022, among which 33,363 were attributed to prostate cancer, indicating that both the incidence and mortality of prostate cancer are among the highest of all male malignancies. Current clinical therapeutic strategies for prostate cancer mainly include androgen deprivation therapy, chemotherapy, and emerging targeted drug therapy. Although chemotherapy represented by docetaxel and targeted therapies have improved patient survival to a certain extent, issues such as dose-limiting toxicity, drug resistance, and high recurrence rates still severely compromise their clinical efficacy [3,4]. In contrast, natural anticancer agents have attracted extensive attention in cancer prevention and treatment due to their prominent advantages, such as low toxicity, good biocompatibility, wide sources, and multi-target regulatory effects [5,6]. Therefore, they provide a new research direction for the clinical treatment of prostate cancer.

Triterpenoids are a class of natural products widely distributed in plants. Among them, pentacyclic triterpenoids have shown promising application prospects in various tumor intervention studies due to their diverse structural types and various pharmacological activities [7,8], and have become a research focus in this field in recent years [9]. Ursolic acid (UA; 3β-hydroxy-12-urs-12-en-28-oic acid) is a typical pentacyclic triterpenoid and a secondary plant metabolite, which is widely present in the stem bark, leaves, or peels of many plants [10]. In recent years, UA has been verified to possess multiple pharmacological activities, including anti-inflammatory [11], antitumor [12], antiviral [13], and antioxidant activities [14], among which its antitumor effect has aroused widespread concern among researchers [10]. Accumulating studies have confirmed that UA exerts significant inhibitory effects on various cancers, including prostate cancer [2], colorectal cancer [15], and breast cancer [16], demonstrating broad-spectrum antitumor potential. However, UA suffers from obvious drawbacks such as poor water solubility and insufficient targeting [17], which greatly restrict its clinical translation and application. Therefore, structural modification of UA represents an effective strategy to improve its pharmaceutical properties, enhance its antitumor activity, and strengthen its targeting ability.

Mitochondria are not only the core site of cellular energy metabolism, producing more than 90% of intracellular ATP via oxidative phosphorylation [18], but also play critical roles in cell apoptosis, signal transduction, and calcium homeostasis regulation [19]. Mitochondrial dysfunction is closely associated with tumorigenesis and tumor progression. Recent studies have confirmed that mitochondria can regulate the death process of tumor cells through multiple pathways, among which the apoptotic pathway and imbalance of cytoskeletal homeostasis serve as core regulatory targets [20]. When cells receive apoptotic stimuli, mitochondria release pro-apoptotic factors such as cytochrome c into the cytoplasm, which in turn activates the caspase cascade and ultimately initiates the apoptotic program [21].

As a core structure that maintains cell morphology and regulates cell functions, the cytoskeleton mainly consists of three types of filamentous structures: microfilaments, microtubules, and intermediate filaments [22]. Among them, microfilaments are assembled from highly conserved actin, and their dynamic polymerization and depolymerization (i.e., the F-actin/G-actin equilibrium) provide the core driving force for cell morphogenesis, migration, and invasion, which are directly related to the integrity and stability of the cytoskeleton [23]. Upon activation by FGF, FGFR1 recruits and activates KRAS via adaptor proteins, thereby initiating the MAPK and PI3K/AKT signaling pathways [24]. Simultaneously, KRAS not only directly regulates the cytoskeleton but also activates Rac1 through PI3K [25]. Rac1, in turn, modulates F-actin assembly and PIP4K2 activity [26,27,28], while PIP4K2 catalyzes the production of PIP2 [29]. PIP2 regulates actin dynamics and provides membrane anchoring sites for gelsolin (GSN) [30,31]. Gelsolin (GSN), an important actin-severing protein, is a key regulator of actin dynamics. Upon calcium activation, it directly cleaves F-actin, thereby altering the F-actin/G-actin ratio, disrupting cytoskeletal integrity, and further affecting key pathophysiological processes such as cell motility and invasion [30,31]. In addition, mitochondrial dysfunction can affect the expression and activity of cytoskeleton regulatory proteins through ROS accumulation and abnormal energy metabolism, revealing a close interactive regulatory relationship between the mitochondrial apoptotic pathway and cytoskeletal remodeling. These two processes coordinately regulate the fate of tumor cells, providing an important multi-target intervention strategy for antitumor drug development. Furthermore, they lay a theoretical foundation for this study to investigate how Compound **25** disrupts cytoskeletal homeostasis and exert antitumor effects by regulating the above key molecules and pathways.

Although a previous review has systematically summarized the extensive research progress on ursolic acid (UA) derivatives and their anti-prostate cancer effects [2], most existing ursolic acid-triphenylphosphine (TPP) conjugates adopt a simple direct conjugation mode or only short alkyl linkers (≤3 methylene units), which significantly limits their structural flexibility and mitochondrial targeting efficiency. Furthermore, the majority of reported UA derivatives mediate anti-prostate cancer activity through a single mechanism (typically mitochondrial apoptosis), without in-depth exploration of synergistic multi-pathway effects.

In this study, we rationally designed, synthesized, and structurally characterized a series of ursolic acid-phosphine derivatives by modifying the C28 carboxyl group of UA with dibromoalkanes of different chain lengths (2–6 methylene units) and further conjugating them with seven diverse lipophilic cationic TPP-derived moieties. Meanwhile, their in vitro and in vivo antitumor activities were systematically evaluated. Based on the remarkable cytotoxicity of Compound **25** against the prostate cancer cell line PC3-M, we further investigated its underlying molecular mechanisms. The results demonstrated that the mitochondria-targeted Compound **25** induced cell apoptosis by regulating the mitochondrial apoptotic pathway. Meanwhile, it inhibited the FGFR1/KRAS/RAC1/PIP4K2 axis and overactivated gelsolin (GSN) by modulating the actin cytoskeleton, thereby disrupting cytoskeletal homeostasis. This dual-action mode not only enhances therapeutic efficacy but also reduces the potential risk of drug resistance, which is distinctly different from the single-pathway pattern of most UA derivatives documented in previous studies. Using in vitro cell experiments and in vivo animal models, we systematically verified the antitumor efficacy and dual mechanism of Compound **25**, and clarified that ursolic acid-phosphine derivatives synergistically regulate mitochondrial-mediated apoptosis and cytoskeletal pathways. These findings provide new candidate compounds, novel targets, and theoretical evidence for the treatment of prostate cancer with active ingredients from traditional Chinese medicine.

## 2. Results

### 2.1. Chemical Synthesis

**Figure 1:**
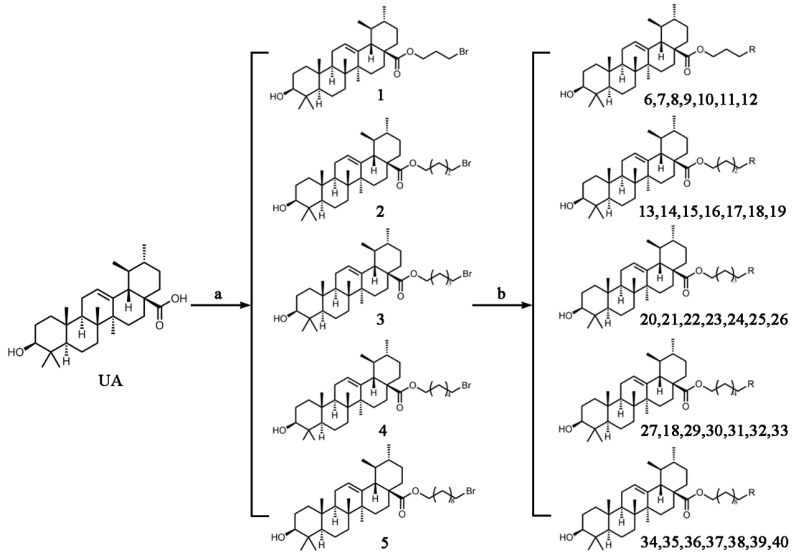
Synthetic route of UA derivatives. (**a**) Acetone, K_2_CO_3_, reflux, 24 h; (**b**) Acetonitrile, K_2_CO_3_, reflux, 24 h, where R is selected from triphenylphosphine, diphenylcyclohexylphosphine, tri-p-tolylphosphine, tris(4-methoxyphenyl)phosphine, tris(4-chlorophenyl)phosphine, tris(3,5-xylyl)phosphine, and tris(o-methoxyphenyl)phosphine.

The hydrogen atom on the carboxyl group (-COOH) at the C28 position of ursolic acid underwent a substitution reaction with the bromine atom in dibromoalkanes. The two moieties were linked via an ester bond to form raw material intermediates 1, 2, 3, 4, and 5. Subsequently, the prepared intermediates were further reacted with seven different lipophilic cation (DLC) moieties, The reaction results are shown in Figure 1, and their specific structures are listed in Table 1, including triphenylphosphine, diphenylcyclohexylphosphine, tri-p-tolylphosphine, and tris(4-methoxyphenyl)phosphine, yielding target products 6–12, 13–19, 20–26, 27–33, and 34–40. The detailed synthetic route is illustrated in Scheme 1. The terminal bromine atom of the ursolic acid intermediate bound to the phosphorus atom (P) in triphenylphosphine-series compounds via nucleophilic substitution, finally generating the target ursolic acid derivatives.

All intermediates and target products were structurally characterized using ^1^H-nuclear magnetic resonance (^1^H-NMR) and ^13^C-nuclear magnetic resonance (^13^C-NMR) spectroscopy. The spectral data confirmed that their structures were consistent with the designed structures. Detailed structural identification of all compounds is provided in the Supporting Information (Figures S1–S40).

### 2.2. Screening of Pharmacological Activities of Ursolic Acid Derivatives Against Various Tumor Cell Lines

To evaluate the in vitro cytotoxicity of ursolic acid derivatives against human tumor cell lines, three representative cancer cell lines—PC3-M, A549, and HepG2—were selected. Cell viability was determined by the Cell Counting Kit-8 (CCK-8) assay after treatment with 40 synthesized ursolic acid-phosphine derivatives. The half-maximal inhibitory concentration (IC_50_) was calculated to quantitatively assess the antiproliferative potency of each derivative. As shown in Table 2 (Supporting Information), ursolic acid-phosphine derivatives exhibited differential and concentration-dependent cytotoxicity against the three tested cell lines.

The results revealed that all derivatives conjugated with tris(3,5-xylyl)phosphine (Compounds **11**, **18**, **25**, **32**, and **39**) displayed potent tumor cytotoxicity, with IC_50_ values ranging from 1.302 ± 0.13 to 17.18 ± 1.87 μM. Among them, Compound **25** showed the most remarkable antiproliferative activity.

Notably, the chain length of the dibromoalkyl intermediate played a critical regulatory role in the cytotoxic potency of ursolic acid-phosphine derivatives. Carbon chain length was closely correlated with antiproliferative activity: cytotoxicity gradually increased with longer linker chains and peaked at five methylene units, followed by a gradual decrease upon further chain extension. This trend was consistently observed across multiple derivative series, verifying that five methylene units represent the optimal linker length for maximum cytotoxicity.

Furthermore, PC3-M cells were the most sensitive to ursolic acid-phosphine derivatives, A549 cells exhibited moderate sensitivity, and HepG2 cells were relatively less sensitive. Among all compounds, Compound **25** exerted the strongest cytotoxicity against the three human tumor cell lines, particularly toward PC3-M cells, with an IC_50_ value of 1.302 ± 0.13 μM. Based on its excellent broad-spectrum cytotoxic profile, PC3-M cells were used as the model cell line, and Compound **25** was selected for subsequent mechanistic investigations in this study.

### 2.3. Compound ***25*** Significantly Inhibits the Growth of PC3-M Cells

**Figure 2:**
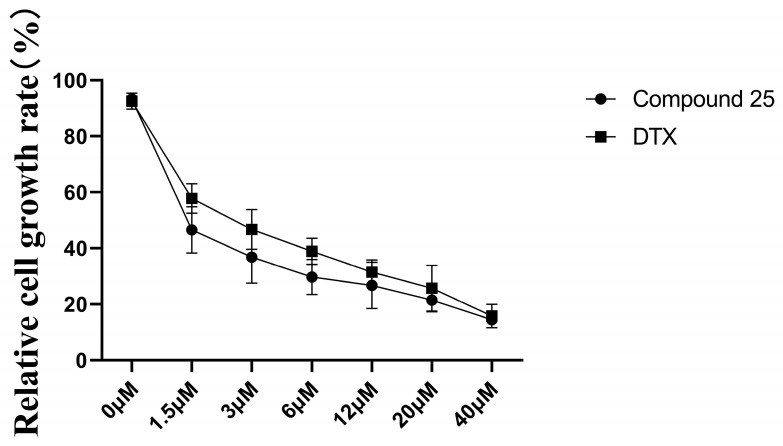
Growth curves of PC3-M cells treated with Compound **25** and docetaxel (0–40 µM). Values are expressed as mean ± standard deviation (*n* = 6).

To systematically evaluate the anti-prostate cancer potential of Compound **25**, parallel control experiments were performed between Compound **25** and the clinically used anti-prostate cancer drug docetaxel. The inhibitory effects of the two agents on the proliferation of PC3-M cells were detected by the CCK-8 assay, and the results are presented in Figure 2. Compound **25** exerted a marked concentration-dependent inhibitory effect on the proliferation of PC3-M cells. At a concentration of 1.5 µM, the relative cell growth rate of the Compound **25**-treated group was 46.52%, which was significantly lower than that of docetaxel at the same concentration (*p* < 0.05).

These results clearly demonstrate that the growth-inhibitory activity of Compound **25** against PC3-M cells is superior to that of the clinical chemotherapeutic drug docetaxel, indicating its outstanding anti-prostate cancer potential. This provides solid in vitro experimental support for the subsequent in-depth investigation of its antitumor mechanism and in vivo activity evaluation.

### 2.4. Compound ***25*** Markedly Inhibits Colony Formation of PC3-M Cells

**Figure 3:**
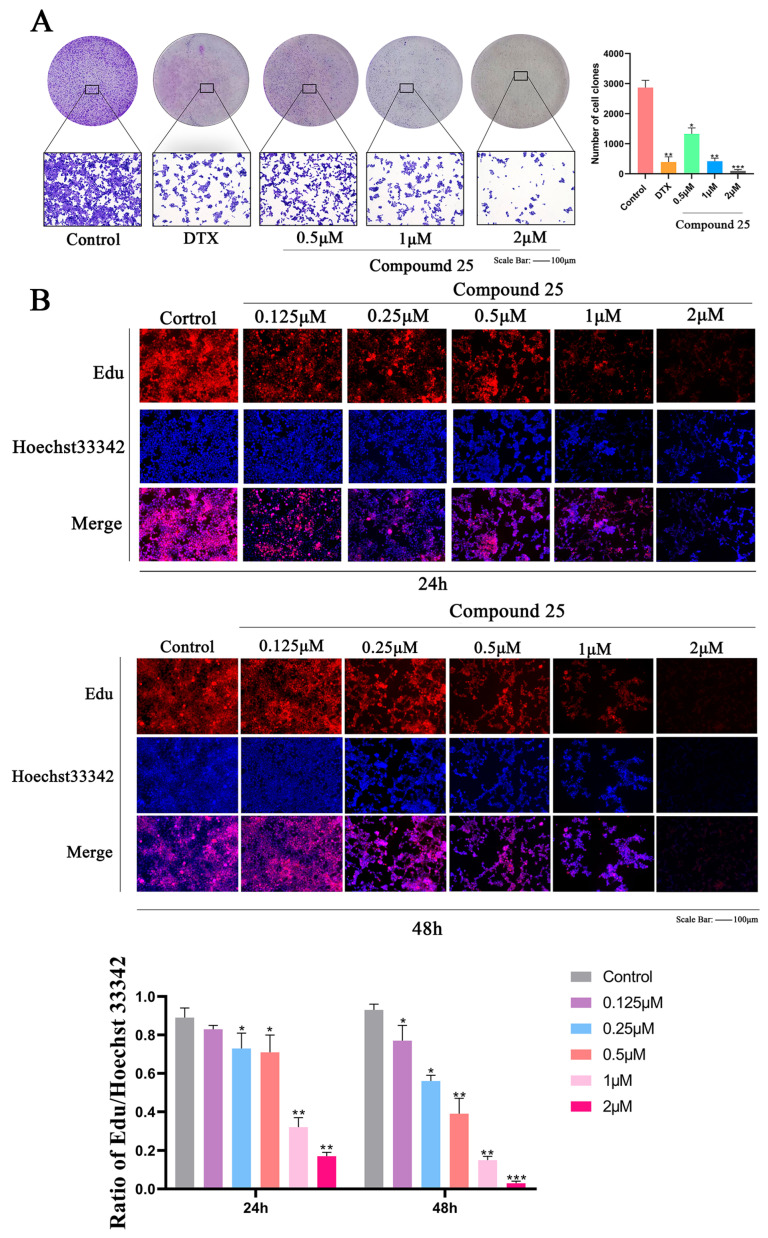
Compound **25** inhibits colony formation and proliferation of PC3-M cells in a dose- and time-dependent manner. (**A**) Effect of different concentrations of Compound **25** and docetaxel (DTX, positive control) on colony formation of PC3-M cells (scale bar = 100 μm). The histogram shows the quantitative analysis of colony numbers. (Red represents EdU staining, blue represents Hoechst 33342 staining, and purple indicates the merged image of the two.) (**B**) Proliferation ability and quantitative histograms of PC3-M cells treated with different concentrations of Compound **25** or DTX for 24 h and 48 h (Scale bar = 100 μm). Red: EdU staining; blue: Hoechst 33342 staining. All data are presented as mean ± standard deviation (SD) of three independent experiments. Compared with the control group, * *p* < 0.05, ** *p* < 0.01, *** *p* < 0.001.

To evaluate the antiproliferative effect of Compound **25** on PC3-M prostate cancer cells, colony formation assays were conducted in this study. As shown in Figure 3A, PC3-M cells in the control group exhibited strong colony-forming ability. In contrast, the number of colonies was slightly reduced in the group treated with 0.5 μM Compound **25**, while colony formation was significantly suppressed in the 1 μM and 2 μM treatment groups. Notably, the inhibitory effect of Compound **25** at the higher concentration (2 μM) was even more pronounced than that of the positive control group.

These results indicate that Compound **25** remarkably inhibits the colony-forming capacity of PC3-M cells in a dose-dependent manner, confirming its potential application value in the treatment of prostate cancer.

### 2.5. Compound ***25*** Significantly Inhibits Proliferation of PC3-M Cells

To further investigate the antiproliferative effect of Compound **25** on PC3-M prostate cancer cells, 5-ethynyl-2′-deoxyuridine (EdU) staining was performed after 24 h and 48 h of treatment, respectively. As shown in Figure 3B, the number of EdU-positive cells gradually decreased with increasing concentrations of Compound **25**, whereas Hoechst 33342 staining confirmed the presence of viable cell nuclei in all treatment groups. These results demonstrate that Compound **25** inhibits the proliferation of PC3-M cells in a dose- and time-dependent manner.

At the 24 h time point, the ratio of EdU-positive cells decreased slightly in the low-concentration groups (0.125 μM and 0.25 μM), while more significant inhibition was observed in the 0.5 μM, 1 μM, and 2 μM groups. This inhibitory effect was further enhanced at 48 h: reduced proliferative cells were detected even at the lowest concentration (0.125 μM), and DNA synthesis was almost completely suppressed in the 1 μM and 2 μM groups. Collectively, these findings indicate that Compound **25** exerts a sustained and potent antiproliferative effect on PC3-M cells, consistent with the long-term inhibition of colony formation observed in the colony formation assay.

### 2.6. Compound ***25*** Significantly Suppresses Migration and Invasion of PC3-M Cells

**Figure 4:**
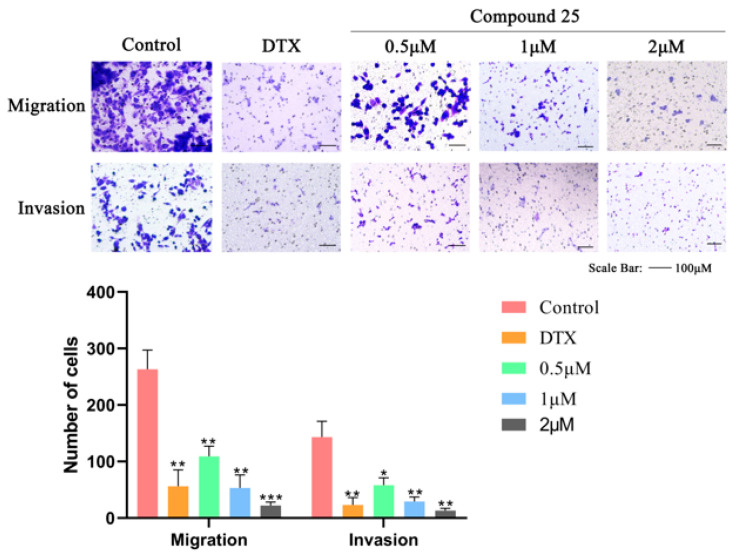
Effects of Compound **25** and docetaxel (DTX, positive control) on migration and invasion of PC3-M cells and corresponding quantitative histograms (Purple represents crystal violet.). (Scale bar = 100 μm) Data are presented as mean ± standard deviation of three independent experiments (*n* = 3). Compared with the control group, * *p* < 0.05, ** *p* < 0.01, *** *p* < 0.001.

Transwell assays were performed to explore the effects of Compound **25** on the migration and invasion of PC3-M cells. As shown in Figure 4, treatment with docetaxel (DTX, positive control) markedly reduced the migratory and invasive capacities of PC3-M cells. Both the migratory and invasive capacities of PC3-M cells were markedly reduced in a concentration-dependent manner after 24 h of treatment with Compound **25**. Notably, the inhibitory effect of Compound **25** at 2 μM was slightly superior to that of the DTX group. These results suggest that Compound **25** effectively inhibits the migration and invasion of PC3-M cells in a dose-dependent manner, indicating its potential to suppress the metastasis of prostate cancer.

### 2.7. Analysis of Differentially Expressed Genes upon Compound ***25*** Treatment

**Figure 5:**
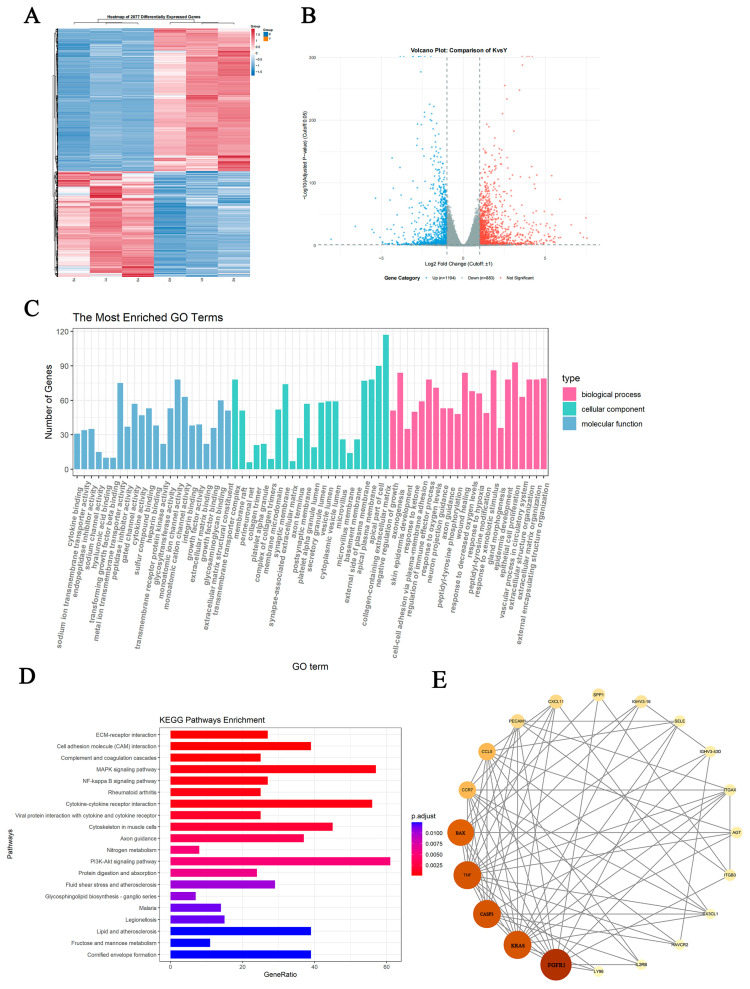
Analysis results of differentially expressed genes. (**A**) Heatmap of expression patterns of DEGs. (**B**) Volcano plot of differentially expressed genes. (**C**) GO enrichment analysis of DEGs between the Compound **25** treatment and control groups. (**D**) Histogram of DEG enrichment in KEGG pathways. (**E**) Protein–protein interaction (PPI) network.

Based on RNA-seq data, differential expression analysis was performed to identify genes regulated by Compound **25** in PC3-M cells. During screening, a fold change (FC) ≥ 1.5 and false discovery rate (FDR) < 0.05 were used as the cutoff criteria. Hierarchical clustering analysis of differentially expressed genes (DEGs) in the heatmap showed satisfactory clustering, indicating good reproducibility of the treatment (Figure 5A). Volcano plots allowed rapid visualization of gene expression differences and their statistical significance between the two groups. Compared with the blank control group, a total of 2114 DEGs were identified in the Compound **25**-treated group, including 896 upregulated genes and 1218 downregulated genes (Figure 5B).

To clarify the biological functions and signaling pathways involved in the DEGs regulated by Compound **25**, Gene Ontology (GO) enrichment analysis and Kyoto Encyclopedia of Genes and Genomes (KEGG) pathway enrichment analysis were conducted. GO enrichment analysis (Figure 5C) revealed that, in the biological process (BP) category, DEGs were mainly involved in actin cytoskeleton organization, actin filament polymerization, microtubule-related processes, and cell adhesion. In the cellular component (CC) category, DEGs were predominantly enriched in the actin cytoskeleton, cytoskeleton, and microtubule-organizing center. In the molecular function (MF) category, DEGs were mainly associated with actin binding, microtubule binding, and structural constituents of the cytoskeleton. Notably, genes encoding cytoskeleton-regulatory proteins, including those involved in actin capping and severing, were significantly enriched.

KEGG enrichment analysis (Figure 5D) showed that DEGs were significantly enriched in multiple tumor-associated pathways, including ECM-receptor interaction, regulation of actin cytoskeleton (e.g., myocyte cytoskeleton, actin cytoskeleton regulation), MAPK signaling pathway, and cytokine-cytokine receptor interaction. Among them, the enrichment of cytoskeleton-related pathways provided transcriptomic evidence that Compound **25** modulates tumor cell migration and invasion, further confirming its synergistic intervention in prostate cancer progression through multiple pathways.

To further explore core regulatory genes, a protein–protein interaction (PPI) network was constructed. Network analysis (Figure 5E) revealed a tightly interconnected functional module, with key genes showing the highest connectivity (Degree), including FGFR1, KRAS, CASP3, and BAX. These results strongly support that Compound **25** simultaneously targets the cytoskeleton signaling axis (FGFR1/KRAS/RAC1/GSN) and the mitochondrial apoptotic signaling axis (CASP3/BAX/BCL2), exerting antitumor effects through crosstalk between these two core pathways.

In summary, transcriptomic analysis indicates that Compound **25** extensively modulates gene expression and significantly disturbs key biological processes, including cytoskeletal remodeling and apoptosis in PC3-M cells.

### 2.8. Compound ***25*** Induces ROS Accumulation and Significantly Reduces Mitochondrial Membrane Potential

**Figure 6:**
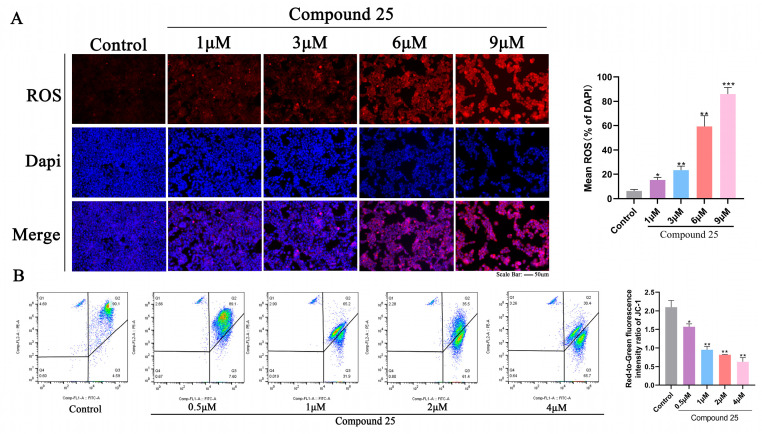
Compound **25** induces the loss of mitochondrial membrane potential in PC3-M cells. (**A**) Changes in ROS levels induced by Compound **25** and corresponding quantitative histograms. (**B**) Flow cytometric results and quantitative histograms of mitochondrial membrane potential changes in PC3-M cells induced by Compound **25**. Compared with the control group, * *p* < 0.05, ** *p* < 0.01, *** *p* < 0.001.

Prolonged elevation of reactive oxygen species (ROS) levels exacerbates oxidative damage to DNA, proteins, and lipids, leading to progressive cellular dysfunction and ultimately mediating apoptosis [32]. To determine whether Compound **25** induces oxidative stress in PC3-M cells, intracellular ROS levels were measured using the ROS-sensitive probe DCFH-DA (red fluorescence), with DAPI counterstaining to visualize cell nuclei. As shown in Figure 6A, the intensity of red fluorescence gradually increased with rising concentrations of Compound **25** (0, 0.5, 1, 2, and 4 μM). DAPI staining (blue fluorescence) was used to label cell nuclei.

In the control group, only negligible red fluorescent signals were detected, reflecting a basal oxidative state. Treatment with 0.5 μM Compound **25** resulted in a moderate increase in ROS-positive cells. This effect was markedly enhanced at 1 μM and 2 μM, with intense red fluorescence observed in most cells. At the highest concentration tested (4 μM), prominent ROS accumulation was accompanied by obvious morphological changes in nuclei as revealed by DAPI staining, indicating severe cellular stress and apoptosis.

The effects of Compound **25** on mitochondrial function were further investigated by measuring mitochondrial membrane potential (ΔΨm) and ROS levels in PC3-M cells. To assess whether Compound **25** triggers mitochondrial dysfunction, JC-1 staining combined with flow cytometry was performed. Representative scatter plots (Figure 6B) showed that control cells mainly exhibited high red fluorescence (Q2), indicating intact mitochondrial membranes. Following treatment with increasing concentrations of Compound **25** (0.5, 1, 2, and 4 μM), the cell population gradually shifted toward the green fluorescence channel (Q4), indicating a reduction in ΔΨm. Moreover, the ratio was significantly decreased in a concentration-dependent manner after Compound **25** treatment (* *p* < 0.01 vs. control group).

Collectively, these results demonstrate that Compound **25** triggers concentration-dependent intracellular ROS accumulation and concomitant dissipation of mitochondrial membrane potential, suggesting that Compound **25** induces oxidative stress and mitochondrial dysfunction, thereby impairing mitochondrial function.

### 2.9. Compound ***25*** Induces Apoptosis in PC3-M Cells by Regulating Mitochondrial Apoptosis-Related Proteins

To verify whether Compound **25** induces PC3-M cell death through the apoptotic pathway, Annexin V-FITC/PI double staining combined with flow cytometry was used to evaluate its apoptosis-inducing effect. PC3-M cells were treated with various concentrations of Compound **25** (1, 2, and 4 µM) for 12 h, stained with Annexin V-FITC and propidium iodide (PI), and analyzed by flow cytometry.

**Figure 7:**
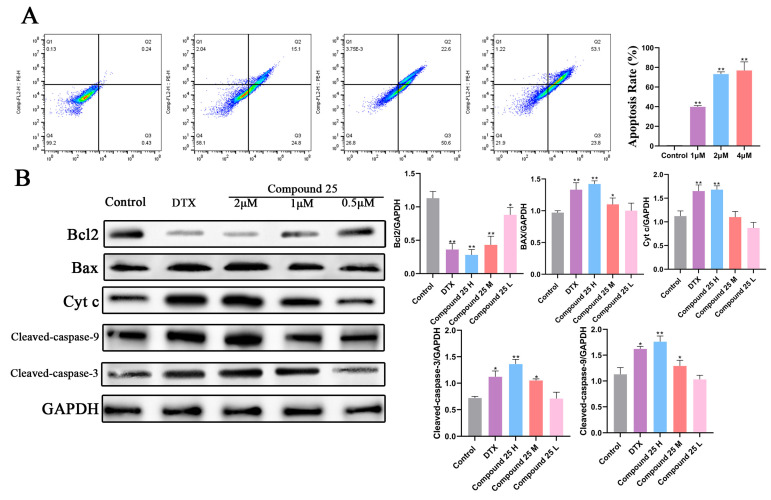
Effect of Compound **25** on apoptosis in PC3-M cells. (**A**) Apoptosis of PC3-M cells assessed by Annexin V-FITC/PI double-staining flow cytometry. Apoptotic rate (%) was calculated as the sum of early and late apoptotic cells. (The color gradient ranging from blue to green, yellow, and red in the dot plots indicates cell density at each coordinate, with blue representing the lowest and red the highest event density, to better visualize cell population distribution.) Data are presented as mean ± SD of three independent experiments. Compared with the control group, ** *p* < 0.01. (**B**) Effect of Compound **25** on the expression levels of proteins related to the mitochondrial apoptotic pathway and corresponding quantitative histograms. Values are expressed as mean ± SD (*n* = 3). Compared with the blank group, * *p* < 0.05, ** *p* < 0.01 for downregulated proteins.

As shown in Figure 7A, only a small number of spontaneous apoptotic cells were observed in the control group, whereas the total apoptotic rate in the Compound **25**-treated groups increased significantly in a concentration-dependent manner. Further analysis revealed that the proportions of both early and late apoptotic cells were markedly elevated with increasing drug concentration. These findings were closely associated with the previously observed reduction in mitochondrial membrane potential (ΔΨm) and accumulation of reactive oxygen species (ROS), confirming the activation of the mitochondrial apoptotic pathway at the phenotypic level.

To confirm the involvement of the mitochondrial apoptotic pathway at the molecular level, Western blot analysis was performed to detect the expression of key apoptosis-regulatory proteins, including Bcl-2, Bax, Cytochrome c, Cleaved-Caspase-9, and Cleaved-Caspase-3. As shown in Figure 7B, the expression levels of Bcl-2 family proteins and components of the caspase cascade exhibited significant concentration-dependent changes with increasing concentrations of Compound **25**.

The anti-apoptotic protein Bcl-2 was significantly downregulated in a concentration-dependent manner (with the most pronounced inhibition in the 2 μM group). In contrast, the pro-apoptotic protein Bax was upregulated dose-dependently, leading to a remarkable decrease in the Bcl-2/Bax ratio. This ratio shift is a critical molecular event for the opening of the mitochondrial permeability transition pore and mitochondrial outer membrane permeabilization (MOMP).

Accompanying MOMP, cytoplasmic Cytochrome c levels were elevated in a concentration-dependent manner, confirming its release from the mitochondrial intermembrane space into the cytosol. This process further triggered the activation of the downstream caspase cascade, as evidenced by the significant upregulation of Cleaved-Caspase-9 and Cleaved-Caspase-3 along the concentration gradient. Docetaxel (DTX), used as a positive control, also induced caspase activation, but its regulatory pattern differed from that of Compound **25**.

In summary, these results demonstrate that Compound **25** induces mitochondrial permeability transition by downregulating Bcl-2 and upregulating Bax, promotes Cytochrome c release, sequentially activates Cleaved-Caspase-9 and Cleaved-Caspase-3, and ultimately initiates the intrinsic mitochondrial apoptotic pathway to mediate PC3-M cell death.

### 2.10. Compound ***25*** Disrupts Cytoskeletal Homeostasis in PC3-M Cells by Inhibiting the FGFR1/KRAS/RAC1/PIP4K2 Signaling Axis

**Figure 8:**
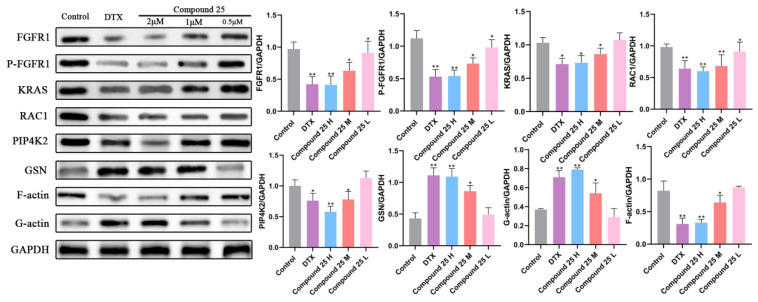
Effect of Compound **25** on the levels of cytoskeleton homeostasis-related proteins in PC3-M cells and corresponding quantitative histograms. Values are expressed as mean ± SD (*n* = 3). Compared with the blank group, * *p* < 0.05, ** *p* < 0.01 for downregulated proteins.

To investigate the molecular mechanism by which Compound **25** induces cytoskeletal destruction in PC3-M cells, Western blot analysis was performed to detect the expression levels of key regulators of cytoskeletal dynamics. As shown in Figure 8, the FGFR1/KRAS/RAC1/PIP4K2 signaling axis exhibited significant concentration-dependent changes after treatment with various concentrations of Compound **25**.

Total FGFR1 protein levels remained relatively stable in all treatment groups. However, compared with the control group, phosphorylated FGFR1 (p-FGFR1) levels were markedly decreased in the 1 μM and 2 μM Compound **25**-treated groups, indicating that Compound **25** directly inhibits the phosphorylation and activation of FGFR1, thereby blocking the initial signal transduction of this receptor tyrosine kinase.

As key downstream effector molecules of FGFR1, the expression levels of KRAS, RAC1, and PIP4K2 were gradually downregulated with increasing concentrations of Compound **25** (0.5, 1, and 2 μM), with the most significant inhibitory effect observed in the 2 μM group (*p* < 0.05). This result is highly consistent with the biological effect of blocked signal transmission along the KRAS/RAC1/PIP4K2 axis following inhibition of FGFR1 activation.

Gelsolin (GSN), a critical severing protein governing actin dynamics, acts as a downstream effector of the above signaling cascade. The results showed that Compound **25** treatment led to a concentration-dependent increase in GSN protein levels, suggesting that inhibition of the FGFR1/KRAS/RAC1/PIP4K2 axis further induces GSN overexpression and enhances its F-actin-severing activity.

Consistent with the enhanced GSN-mediated actin cleavage, the ratio of filamentous actin (F-actin) to globular actin (G-actin) was significantly decreased after Compound **25** treatment, indicating impaired integrity of the actin cytoskeleton. Docetaxel, a microtubule stabilizer used as a positive control, showed a distinct regulatory pattern on the F-actin/G-actin ratio, further supporting the specificity of Compound **25** in targeting the actin cytoskeleton.

Collectively, these results confirm that Compound **25** inhibits FGFR1 phosphorylation and the downstream KRAS/RAC1/PIP4K2 signaling axis, induces GSN overexpression and subsequent actin cytoskeleton destabilization, thereby jointly disrupting cytoskeletal homeostasis in PC3-M cells and ultimately suppressing cell migration and invasion.

### 2.11. Molecular Docking Simulation of the Binding Between Compound ***25*** and FGFR1

**Figure 9:**
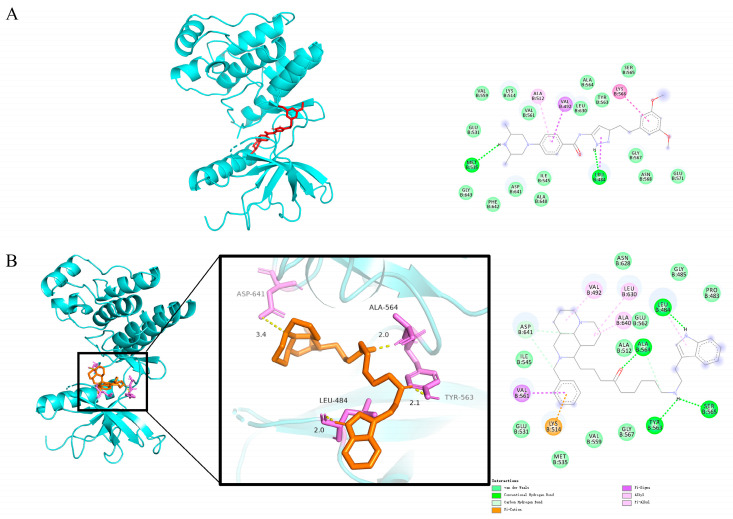
Molecular docking simulation of Compound **25** with FGFR1. (**A**) Molecular docking results of the FGFR1 inhibitor AZD4547 with FGFR1. The 3D overview shows the protein in cyan cartoon representation and AZD4547 as red sticks. The corresponding 2D diagram illustrates binding interactions between AZD4547 and key FGFR1 residues. (**B**) Three-dimensional and two-dimensional interaction diagrams of the binding mode of Compound **25** with FGFR1. The zoomed-in view of the binding pocket shows Compound **25** (orange sticks) and interacting FGFR1 residues (purple sticks), with hydrogen bond distances (Å) labeled. The 2D diagram details the interactions between Compound **25** and FGFR1 residues..

To investigate the binding mode and interaction mechanism between Compound **25** and FGFR1, molecular docking simulation was performed using the FGFR1-selective inhibitor AZD4547 as a positive control. As shown in the docking results (Figure 9A), the binding affinity of Compound **25** to FGFR1 reached −8.79 kcal/mol, which was superior to that of the positive control drug AZD4547 (−8.51 kcal/mol), indicating strong spontaneous binding capacity and a stable conformation of the formed complex.

Further interaction analysis (Figure 9B) revealed that the binding system was primarily stabilized by van der Waals forces and conventional hydrogen bonds. Specifically, the core structure of Compound **25** formed multiple stable hydrophobic interactions and a hydrogen-bond network with key residues of the receptor protein, including LEU-484, ALA-564, TYR-563, and ASP-641.

This binding mode allows Compound **25** to precisely fit into the active pocket of the target protein, interfering with conformational changes and signal transduction through steric hindrance and energy complementation. The molecular docking results further confirmed the high binding affinity and specificity of Compound **25** toward FGFR1, enabling effective targeting of the protein’s active center. These findings provide a direct structural biological basis for the antitumor effect of Compound **25** via regulation of the FGFR1/KRAS/RAC1/PIP4K2 signaling axis.

### 2.12. Compound ***25*** Inhibits Tumor Growth in a PC3-M Xenograft Mouse Model

To evaluate the in vivo antitumor efficacy and safety of Compound **25**, a subcutaneous RM-1 allograft tumor model was established in immunocompetent C57BL/6 mice. Tumor-bearing mice were randomly divided into five groups (*n* = 10 per group): control group, positive control group (docetaxel), low-dose Compound **25** group, medium-dose Compound **25** group, and high-dose Compound **25** group.

**Figure 10:**
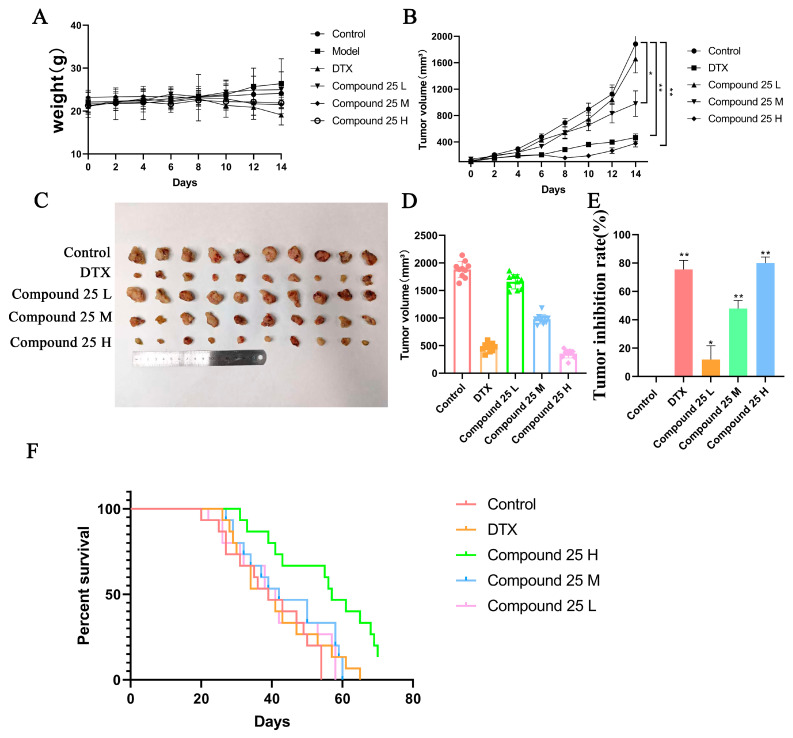
Antitumor effects of Compound **25** in a mouse xenograft model. (**A**) Body weight changes in mice during treatment. Mice were intraperitoneally injected with normal saline, docetaxel (10 mg/kg), or Compound **25** at low (5 mg/kg), medium (10 mg/kg), and high (15 mg/kg) doses twice a week for 14 consecutive days. (**B**) Tumor growth curves during treatment. Tumor volume was measured every two days. (**C**) Images of isolated tumor tissues in each group after 14 days. (**D**) Tumor volume plots over the 14-day treatment period. (**E**) Tumor inhibition rates. (**F**) Survival curves of tumor-bearing mice. Data are expressed as mean ± SD (*n* = 10). Compared with the control group, * *p* < 0.05, ** *p* < 0.01.

During the 14-day treatment period, tumor volume and body weight were monitored every two days, and the results are shown in Figure 10A,B. Compared with the control group, no significant body weight loss was observed in mice treated with different doses of Compound **25**, indicating favorable in vivo safety. Meanwhile, tumor growth was markedly inhibited in a dose-dependent manner, with the most pronounced inhibitory effect observed in the high-dose group (Compound **25** H).

After the mice were sacrificed, tumor tissues were dissected (Figure 10C,D). Tumor volume increased significantly in the control group, whereas it was reduced to varying degrees in all treatment groups. Both the high-dose group and the positive control group exhibited remarkable tumor-suppressive effects. Statistical results of the tumor inhibition rate are presented in Figure 10E; the inhibition rate of the high-dose group was close to that of docetaxel, with a statistically significant difference (*p* < 0.01).

In addition, survival curve analysis (Figure 10F) revealed that Compound **25** treatment significantly prolonged the survival time of tumor-bearing mice, and the survival benefit became more prominent with increasing dosage. The median survival time of mice in the high-dose group was significantly longer than that in the control and low-dose groups.

In conclusion, Compound **25** effectively inhibits the growth of PC3-M xenografts in vivo, prolongs the survival of tumor-bearing mice, and exhibits favorable biosafety, demonstrating excellent in vivo antitumor potential.

### 2.13. Histopathological Observation

To further evaluate the in vivo safety of Compound **25**, histopathological examination was performed on major organs, including the heart, liver, spleen, lung, and kidney, using hematoxylin and eosin (H&E) staining. Tissue sections from the blank group, model group, positive control group, and Compound **25** treatment groups (low, medium, and high doses) were analyzed to assess morphological abnormalities, inflammatory infiltration, cellular injury, and structural damage. As shown in Figure S41, no obvious pathological changes were observed in the visceral tissues of mice in the control group and all Compound **25**-treated groups, including intact cellular structure, absence of inflammatory infiltration, necrosis, fibrosis, or other abnormal manifestations.

### 2.14. TUNEL Assay Results

**Figure 11:**
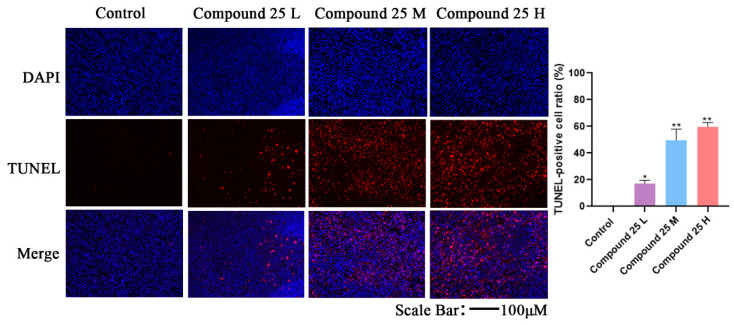
TUNEL assay results and quantitative histograms of apoptosis induced by Compound **25** in tumor tissues in vivo. TUNEL staining images (Magnification × 100). Data are presented as mean ± SD (*n* = 3). (Representative immunofluorescence images showing TUNEL-positive apoptotic cells (red fluorescence) and DAPI-stained cell nuclei (blue fluorescence) in different groups.) * *p* < 0.05, ** *p* < 0.01.

To further verify the in vivo antitumor mechanism and in situ apoptosis-inducing ability of Compound **25**, a TUNEL assay was used for quantitative analysis of apoptotic cells in tumor tissues. As shown in Figure 11, only a few TUNEL-positive cells were observed in tumor tissues of the control group, indicating a low level of spontaneous apoptosis. In contrast, the number of TUNEL-positive cells in Compound **25**-treated groups increased in a dose-dependent manner.

These results were consistent with in vitro apoptosis assays, confirming that Compound **25** can effectively induce tumor cell apoptosis in vivo. Combined with the finding that no obvious pathological damage was observed in major organs, Compound **25** can specifically induce apoptosis in tumor cells without significant toxicity to normal tissues, further supporting its potential as an antitumor candidate agent.

## 3. Discussion

Prostate cancer is one of the most common malignant tumors in men worldwide, and its clinical treatment is hindered by bottlenecks such as drug resistance and severe side effects [33]. Therefore, the development of novel targeted drugs with high efficacy and low toxicity holds great clinical significance. As a natural pentacyclic triterpenoid compound, ursolic acid has attracted extensive attention due to its broad-spectrum antitumor activity and favorable biocompatibility. However, its clinical translation is limited by poor water solubility and weak targeting ability [34].

In this study, structural modification was performed on the C28 carboxyl group of ursolic acid by introducing a mitochondria-targeting moiety. Forty ursolic acid derivatives were successfully synthesized, and Compound **25**, which exhibited the optimal activity, was screened out. The molecular mechanism by which Compound **25** synergistically induces PC3-M cell death through multiple signaling pathways was systematically elucidated, providing new experimental evidence for the structural modification of natural products and targeted therapy of prostate cancer.

In this study, dibromoalkanes were conjugated to the C28 carboxyl group (-COOH) of ursolic acid to form intermediates 1–5. Triphenylphosphine, a classic mitochondria-targeting carrier, can accumulate in the mitochondrial matrix driven by the mitochondrial membrane potential owing to its cationic property [35]. The prepared intermediates were further reacted with seven lipophilic cationic moieties, including triphenylphosphine, diphenylcyclohexylphosphine, tri-p-tolylphosphine, and tris(4-methoxyphenyl)phosphine, yielding target products 6–40.

In addition, cytotoxicity assays revealed that among the three tested tumor cell lines (PC3-M, A549, HepG2), PC3-M prostate cancer cells were the most sensitive to the target derivatives. This phenomenon may be related to differences in mitochondrial membrane potential, metabolic characteristics, and target expression levels among different tumor cells, suggesting that Compound **25** may exert relatively specific therapeutic advantages against prostate cancer. Notably, all structurally modified ursolic acid derivatives exhibited significantly enhanced cytotoxicity compared to ursolic acid itself. Among them, compound **25**, bearing a tris(3,5-xylyl)phosphine group at the C28 position with an alkyl chain length of five methylene units, showed the strongest cytotoxicity, indicating that both the type of triphenylphosphine moiety and the alkyl chain length play critical roles in regulating the antitumor activity of ursolic acid derivatives. This finding is consistent with previous studies, which demonstrated that the introduction of lipophilic cationic groups (triphenylphosphine-based moieties) improves the cell membrane permeability of triterpenoids, enhances tumor-targeting ability, and thereby elevates antitumor efficacy. The structures of all derivatives were confirmed by ^1^H NMR and ^13^C NMR spectroscopy, verifying their successful synthesis and laying a solid foundation for subsequent activity screening and mechanistic studies.

Meanwhile, to systematically evaluate the anti-prostate cancer potential of Compound **25**, the CCK-8 assay was used to compare the proliferation-inhibitory effects of Compound **25** and docetaxel on PC3-M cells in parallel. The results showed that Compound **25** inhibited PC3-M cell proliferation in a significant concentration-dependent manner, with superior inhibitory activity to docetaxel, establishing a reliable in vitro basis for further investigation of its antitumor mechanism and in vivo activity.

In vitro functional assays demonstrated that Compound **25** suppressed the proliferation, migration, and invasion of PC3-M cells in a dose- and time-dependent manner, which are key characteristics of a promising antitumor candidate. Transcriptomic analysis revealed that differentially expressed genes were mainly enriched in apoptosis and cytoskeleton regulation pathways, with FGFR1, KRAS, CASP3, and BAX as core regulatory genes, providing important clues for exploring the molecular mechanism of Compound **25**.

This study demonstrated that Compound **25** reduced mitochondrial membrane potential and promoted intracellular reactive oxygen species (ROS) accumulation, thereby impairing mitochondrial structure and function. Flow cytometric analysis using Annexin V-FITC/PI double staining further confirmed that Compound **25** induced apoptosis in PC3-M cells in a concentration-dependent manner. Western blot analysis showed that Compound **25** regulated the balance of Bcl-2 family proteins by downregulating the anti-apoptotic protein Bcl-2 and upregulating the pro-apoptotic protein Bax, increasing the Bax/Bcl-2 ratio and promoting cytochrome c release from mitochondria into the cytoplasm. This process ultimately activated the Caspase-9/Caspase-3 cascade and initiated the mitochondrial apoptotic pathway, verifying that Compound **25** exerts antitumor effects through the mitochondria-mediated apoptotic pathway.

The cytoskeleton is closely associated with tumor cell proliferation, migration, and invasion [36], and its abnormal remodeling is a hallmark of tumor progression [37]. In this study, Western blot analysis further demonstrated that Compound **25** significantly reduced the levels of phosphorylated FGFR1, downregulated the expression of KRAS, RAC1, and PIP4K2, and simultaneously upregulated the expression of the downstream effector GSN. This regulatory effect led to a decrease in the F-actin/G-actin ratio, instability of actin microfilaments, disruption of the integrity of the actin cytoskeleton, and subsequent inhibition of tumor cell proliferation and migration. The inhibitory effect of Compound **25** on this pathway may be the main mechanism underlying its suppression of PC3-M cell migration and invasion. This discovery enriches the antitumor mechanism of Compound **25**, indicating that it induces tumor cell apoptosis through multiple pathways, which helps enhance antitumor efficacy and reduce the risk of drug resistance. Molecular docking simulation further confirmed the potential binding mode between Compound **25** and FGFR1, providing a theoretical basis for the regulation of the cytoskeleton by Compound **25**.

In vivo experiments further confirmed the antitumor activity and biosafety of Compound **25**. In tumor-bearing mouse models, Compound **25** significantly inhibited tumor growth in a dose-dependent manner, and the antitumor effect of the high-dose group was comparable to that of the positive control docetaxel. Meanwhile, Compound **25** effectively prolonged the survival of tumor-bearing mice. More importantly, no significant body weight loss was observed in mice of all dose groups, no obvious pathological abnormalities were found in major organs, and Compound **25** specifically induced apoptosis in tumor tissues in vivo. These results fully demonstrate the favorable in vivo safety and tumor-targeting ability of Compound **25**, providing critical support for its future clinical translation.

Despite the significant progress achieved in this study, certain limitations still exist that need to be addressed in future research. Although the structural modification of ursolic acid with triphenylphosphine-based moieties effectively enhanced its mitochondrial targeting and antitumor activity, the delivery efficiency of ursolic acid and its derivatives in vivo could be further improved. Notably, relevant research has been reported that focuses on triphenylphosphine-modified triblock copolymer nanoparticles for the mitochondrial-targeted delivery of ursolic acid in cancer therapy, providing a feasible direction for optimizing the delivery of ursolic acid [38]. As a classic mitochondria-targeting molecule, triphenylphosphine can be fabricated into nanoparticles to construct a more efficient targeted delivery platform, which is expected to further improve the bioavailability of ursolic acid and its derivatives (including Compound **25**) and reduce their non-specific distribution in normal tissues. Therefore, future research will focus on developing triphenylphosphine-based nanoparticles for the targeted delivery of ursolic acid and its derivatives, aiming to establish a more efficient and safe targeted delivery system that can further enhance the therapeutic effect of ursolic acid derivatives on prostate cancer and promote their clinical translation. Additionally, the long-term in vivo toxicity and pharmacokinetic characteristics of such nanoparticle-delivered ursolic acid will be systematically investigated to provide a more comprehensive theoretical and experimental basis for its clinical application.

In conclusion, Compound **25**, a ursolic acid derivative obtained through structural modification in this study, exerts synergistic anti-prostate cancer effects via multiple pathways by regulating the mitochondrial apoptotic pathway and disrupting cytoskeletal homeostasis through inhibition of the FGFR1/KRAS/RAC1/PIP4K2 signaling axis. It exhibits remarkable antitumor activity in vitro and in vivo as well as favorable safety profiles. This study not only provides a novel strategy for the structural modification of ursolic acid but also offers a new candidate compound and therapeutic target for the targeted treatment of prostate cancer, with important theoretical significance and clinical application value.

## 4. Materials and Methods

### 4.1. Reagents and Instruments

Ursolic acid (Cat. No. U118635, China, purity ≥ 98% by HPLC), 1,3-Dibromopropane (Cat. No. D104778), 1,4-dibromobutane (Cat. No. D105749), 1,5-dibromopentane (Cat. No. D106538), 1,6-dibromohexane (Cat. No. D106541), 1,10-dibromodecane (Cat. No. D103590), triphenylphosphine (Cat. No. T104475), diphenylcyclohexylphosphine (Cat. No. C115369), tri-p-tolylphosphine (Cat. No. T115585), tris(4-methoxyphenyl)phosphine (Cat. No. T115587), tris(4-chlorophenyl)phosphine (Cat. No. T115602), tris(3,5-dimethylphenyl)phosphine (Cat. No. T123980), tris(2-methoxyphenyl)phosphine (Cat. No. T115589), and 4 Å molecular sieves (Cat. No. M103739) were purchased from Shanghai Aladdin Biochemical Technology Co., Ltd., Shanghai, China. Methanol, dichloromethane, acetonitrile, deuterated chloroform, and other reagents were obtained from Beijing Chemical Works Co., Ltd., Beijing, China.

The structures of all compounds were confirmed by ^1^H NMR and ^13^C NMR spectroscopy, recorded at 300 MHz using CDCl_3_ as the solvent, and supported by the known signals of the ursolic acid core and characteristic resonances of ester and phosphonium moieties. An inverted fluorescence microscope (Phenix Optical Co., Ltd., Shangrao, China) model XDS200-PH/PH100-3B41L-IPL) was used for cellular fluorescence imaging, TUNEL staining, and mitochondrial localization assays. A flow cytometer (BD FACSCanto II, BD Biosciences, San Jose, CA, USA) was employed to measure mitochondrial membrane potential (ΔΨm). A microplate reader (BD Biosciences, USA, model FACSVerse/FACSCanto II) was used for CCK-8 and EdU proliferation assays. A carbon dioxide incubator (Shanghai Yiheng Scientific Instruments Co., Ltd., Shanghai, China. model BPN-50CH (UV)) was used for cell culture.

### 4.2. Chemical Synthesis

#### 4.2.1. Preparation of Ursolic Acid-Phosphine Derivatives

##### Preparation of Intermediates

Ursolic acid (UA, 1.0 mmol) was dissolved in 20 mL of acetone. An appropriate amount of anhydrous potassium carbonate was added, followed by the slow addition of 1,3-dibromopropane, 1,4-dibromobutane, 1,5-dibromopentane, 1,6-dibromohexane, or 1,10-dibromodecane (molar ratio to UA = 5:1). The mixture was heated to reflux and reacted at a constant temperature for 24 h, with the reaction progress monitored by thin-layer chromatography (TLC). After completion of the reaction, the mixture was concentrated under reduced pressure and purified by silica gel column chromatography (dichloromethane/methanol = 20:1) to afford Intermediates 1–5.

##### Preparation of Target Compounds

The corresponding intermediate (1.0 mmol) was dissolved in 20 mL of acetonitrile. Triphenylphosphine, diphenylcyclohexylphosphine, tri-p-tolylphosphine, tris(4-methoxyphenyl)phosphine, tris(4-chlorophenyl)phosphine, tris(3,5-dimethylphenyl)phosphine, or tris(2-methoxyphenyl)phosphine was added, and the mixture was heated to reflux for 24 h. After the reaction, the solvent was removed under reduced pressure, and the residue was purified by silica gel column chromatography (dichloromethane/methanol = 20:1) to obtain Derivatives 6–40.

### 4.3. Cell Experimental Methods

#### 4.3.1. Cell Lines and Materials

Human prostate cancer cells (PC3-M, Cat. No. CL-0304), human lung adenocarcinoma cells (A549, Cat. No. CL-0016), human hepatoblastoma cells (HepG2, Cat. No. CL-0103), and mouse prostate cancer cells (RM-1, Cat. No. CL-0198) were purchased from Wuhan Procell Life Technology Co., Ltd. (Wuhan, China). All cell lines were authenticated by short tandem repeat (STR) profiling and confirmed to be free of mycoplasma contamination prior to use, in compliance with MDPI’s ethical guidelines for research involving cell lines. Docetaxel (Cat. No. D107319) was obtained from Shanghai Aladdin Biochemical Technology Co., Ltd. High-glucose DMEM medium was purchased from Wuhan Procell Life Technology Co., Ltd. Fetal bovine serum (FBS) was obtained from CLARK Bioscience, Richmond, VA, USA. Phosphate-buffered saline (PBS), trypsin-EDTA digestion solution, penicillin, streptomycin, superoxide dismutase (SOD), malondialdehyde (MDA), Trizol reagent, RIPA lysis buffer, and the ROS assay kit were purchased from Beijing Solarbio Science & Technology Co., Ltd., Beijing, China. CCK-8 assay kit, JC-1 mitochondrial membrane potential assay kit, and PVDF membranes were obtained from Shanghai Beyotime Biotechnology Co., Ltd., Shanghai, China. All primary antibodies used for Western blot analysis (Cytochrome c, Bcl-2, Cleaved Caspase-3, Cleaved Caspase-9, Bax, GAPDH, FGFR1, KRAS, RAC1, PIP4K2, GSN) were purchased from Wanlei Biotechnology Co., Ltd., Shenyang, China.

#### 4.3.2. Cell Culture

PC3-M, A549, and HepG2 cells were cultured in DMEM medium supplemented with 10% fetal bovine serum, 100 U/mL penicillin, and 100 μg/mL streptomycin and maintained in a constant-temperature incubator at 37 °C with 5% CO_2_. Cells were passaged every 2–3 days, and cells in the logarithmic growth phase were used for experiments. All test compounds were dissolved in dimethyl sulfoxide (DMSO) to prepare a 100 mM stock solution.

#### 4.3.3. CCK-8 Assay for Cytotoxicity

Cells in the logarithmic growth phase were seeded into 96-well plates at a density of 1 × 10^4^ cells per well. After overnight incubation for adherence, cells were treated with various concentrations of the test compound (0.75, 1.5, 3, 6, 12, 20, 40, 80 μM) with three replicate wells per concentration. A blank control group was set up simultaneously, and cells were cultured for another 24 h. Then, 10 μL of CCK-8 reagent was added to each well. After incubation for 2 h, the optical density (OD) at 450 nm was measured using a microplate reader. Cell viability and IC_50_ values were calculated. The IC_50_ values were determined by nonlinear regression analysis using GraphPad Prism 8.0.2 software.

#### 4.3.4. Colony Formation Assay

PC3-M cells in the logarithmic growth phase were seeded into 6-well plates at 1000 cells per well. After 24 h of culture for adherence, cells were treated with different concentrations of Compound **25** (0, 0.5, 1, 2 μM) and cultured for 14 days. At the end of the culture period, the medium was discarded, and cells were washed twice with PBS, fixed with 4% paraformaldehyde for 30 min, stained with 0.1% crystal violet for 20 min, washed with distilled water, and air-dried. Colonies containing ≥ 50 cells were counted as valid colonies.

#### 4.3.5. EDU Cell Proliferation Assay

PC3-M cells were seeded into 24-well plates at 5 × 10^4^ cells per well. After 24 h of culture, cells were treated with various concentrations of Compound **25** (0, 0.125, 0.25, 0.5, 1, 2 μM) and cultured for another 24 h or 48 h. EdU working solution (final concentration 10 μM) was added, and cells were incubated in the dark for 2 h. Then, cells were fixed with 4% paraformaldehyde for 30 min, permeabilized with 0.5% Triton X-100 for 15 min, incubated with Click reaction solution in the dark for 30 min, and stained with DAPI for 10 min. The percentage of EdU-positive cells was observed and counted under a fluorescence microscope.

#### 4.3.6. Transwell Migration and Invasion Assays

Migration assay: PC3-M cells in the logarithmic growth phase were starved for 12 h in serum-free DMEM. The cell concentration was adjusted to 1 × 10^6^ cells/mL, and 200 μL of cell suspension was added to the upper chamber of Transwell inserts, while 600 μL of DMEM containing 10% FBS was added to the lower chamber. Cells were treated with various concentrations of Compound **25** (0, 0.5, 1, 2 μM) and incubated for 24 h. Non-migrated cells on the upper surface were removed with a cotton swab. Cells were fixed with 4% paraformaldehyde, stained with crystal violet, and migrated cells were counted under a microscope.

Invasion assay: The upper chambers of Transwell inserts were pre-coated with Matrigel (diluted 1:8). The remaining procedures were the same as those for the migration assay. Invasive cells were counted after 24 h of incubation.

#### 4.3.7. Flow Cytometric Analysis of Apoptosis

PC3-M cells were treated with different concentrations of Compound **25** for 24 h. Both floating and adherent cells were collected and washed twice with pre-cooled PBS. Apoptosis was detected using an Annexin V-FITC/PI apoptosis detection kit according to the manufacturer’s instructions. Annexin V-FITC and PI staining solution were added sequentially, followed by incubation for 15 min at room temperature in the dark. Apoptotic rates were analyzed by flow cytometry. Total apoptosis was defined as the sum of early and late apoptotic cell populations. Quantitative analysis was performed using FlowJo 10.8.1 software.

#### 4.3.8. RNA Sequencing and Bioinformatics Analysis

PC3-M cells were divided into a control group and a Compound **25**-treated group, with three biological replicates per group. Total RNA was extracted using the Trizol method, and RNA purity and integrity were verified. RNA sequencing was performed on the Illumina NovaSeq 6000 platform. Differentially expressed genes were screened with thresholds of |log_2_FC| > 1 and *p*-adjust < 0.05. GO and KEGG pathway enrichment analyses were performed using the ClusterProfiler package (v4.19.6). The protein–protein interaction (PPI) network was constructed using the STRING database (https://cn.string-db.org/) (accessed on 15 August 2025) and visualized with Cytoscape 3.9.1 software.

#### 4.3.9. ROS Detection

Intracellular ROS levels were measured using the DCFH-DA probe. PC3-M cells were seeded in 24-well plates and cultured for 24 h, then treated with various concentrations of Compound **25** (0, 1, 3, 6, 9 μM) for another 24 h. DCFH-DA working solution was added, followed by incubation at 37 °C for 30 min in the dark. After three washes with PBS, cells were stained with DAPI for 10 min. Fluorescence images were captured under a fluorescence microscope, and fluorescence intensity was quantitatively analyzed using ImageJ software (v1.54r).

#### 4.3.10. Mitochondrial Membrane Potential (ΔΨm) Assay

Mitochondrial membrane potential was detected using JC-1 staining combined with flow cytometry. PC3-M cells were seeded in 6-well plates and cultured for 24 h, then treated with different concentrations of Compound **25** (0, 0.5, 1, 2, 4 μM) for 24 h. Cells were collected, washed twice with PBS, and incubated with JC-1 staining solution at 37 °C for 20 min in the dark. Fluorescence intensity was analyzed by flow cytometry.

#### 4.3.11. Western Blot Analysis

PC3-M cells were seeded in 6-well plates and cultured for 24 h, then treated with various concentrations of Compound **25** (0, 0.5, 1, 2, 4 μM) for 24 h. A positive control group was also established. Cells were harvested and lysed on ice for 30 min in RIPA lysis buffer containing protease and phosphatase inhibitors. Lysates were centrifuged at 12,000 rpm for 15 min, and supernatants were collected. Protein concentration was determined using the BCA method. A total of 50 μg of denatured protein was separated by SDS-PAGE and transferred to a PVDF membrane. The membrane was blocked with 5% non-fat milk for 1 h, then incubated with primary antibodies (1:1000 dilution) at 4 °C overnight. After three washes with TBST, the membrane was incubated with secondary antibodies (1:5000 dilution) at room temperature for 1 h. Protein bands were visualized using ECL chemiluminescence reagent and quantified using a gel imaging system.

#### 4.3.12. Molecular Docking Simulation

The crystal structure of FGFR1 was downloaded from the PDB database (PDB ID: 6 MZQ). Water molecules and original ligands were removed, and hydrogen bond repair was performed using PyMOL 3.1.10 software. The structure of Compound **25** was drawn and optimized via energy minimization using Chem3D 2020 software. Molecular docking was performed using AutoDock Vina (v1.2.7). The grid box was set to cover the active pocket of FGFR1, and other parameters were used as defaults. The conformation with the lowest binding energy was selected for interaction analysis.

### 4.4. Animal Experimental Methods

#### 4.4.1. Experimental Animals

Fifty specific-pathogen-free (SPF) C57BL/6 male mice (6–8 weeks old) were purchased from Beijing Sibefu Biotechnology Co., Ltd., Beijing, China. (SYXK (Ji) 2023-0021). All mice were housed in an SPF-class animal facility, fed with standard feed and provided with distilled water, allowing free access to food and water. After 7 days of acclimatization, the xenograft tumor model was established. All animal experimental protocols were approved by the Institutional Animal Care and Use Committee (IACUC) of Jilin Agricultural University (Approval No. 2024011003).

#### 4.4.2. Establishment of Mouse Xenograft Model and Drug Administration

After one week of adaptive feeding, C57BL/6 mice were subcutaneously injected with 1 × 10^6^ RM-1 cells in the right axilla. When tumor volume reached 100–150 mm^3^, mice were randomly divided into 5 groups (*n* = 10 per group): control group (normal saline), positive control group (docetaxel, 10 mg/kg), low-dose Compound **25** group (5 mg/kg), medium-dose group (10 mg/kg), and high-dose group (15 mg/kg). All drugs were administered by intraperitoneal injection twice a week for 14 consecutive days. The control and model groups received an equal volume of normal saline. Body weight and tumor volume were measured every 2 days during treatment.

#### 4.4.3. Evaluation of Antitumor Activity In Vivo

After 14 days of administration, mice were sacrificed by cervical dislocation. Tumor tissues were dissected and weighed, and the tumor inhibition rate was calculated. Survival of mice was recorded, and Kaplan–Meier survival curves were plotted to evaluate the effect of Compound **25** on the survival of tumor-bearing mice.

#### 4.4.4. Histopathological Analysis

Major organs, including the heart, liver, spleen, lung, and kidney, were harvested and fixed in 4% paraformaldehyde for 24 h, followed by conventional paraffin embedding and sectioning at 5 μm thickness. After hematoxylin–eosin (H&E) staining, dehydration, clearing, and mounting, tissue morphology was observed under a light microscope.

#### 4.4.5. TUNEL Assay

Xenograft tumor tissues were embedded in paraffin and sectioned routinely. Apoptosis in tumor tissues was detected using a TUNEL apoptosis detection kit according to the manufacturer’s instructions. After dewaxing to water, sections were digested with proteinase K for 15 min and washed 3 times with PBS. TUNEL reaction mixture was added, followed by incubation at 37 °C for 1 h in the dark. Sections were then stained with DAPI for 10 min and observed under a fluorescence microscope. All experiments were repeated independently three times, and the mean values were calculated.

### 4.5. Statistical Analysis

All experimental data are expressed as mean ± standard deviation (mean ± SD). Statistical analysis was performed using SPSS 26.0 and GraphPad Prism 8.0.2 software. One-way analysis of variance (ANOVA) was used for comparisons among multiple groups. *p* < 0.05 was considered statistically significant.

## 5. Conclusions

In summary, this study not only provides scientific evidence for the structural modification of ursolic acid by introducing mitochondria-targeting triphenylphosphine moieties, confirming the potential of Compound **25** as a highly effective and low-toxic antitumor candidate, but also establishes a novel approach for prostate cancer targeted therapy by identifying the coordinated regulation of mitochondrial apoptosis and cytoskeletal homeostasis (via the FGFR1/KRAS/RAC1/PIP4K2/GSN axis) as a core mechanism. This approach involves developing novel antitumor agents from natural products and identifying precise multi-target regulatory mechanisms within cancer cells. Furthermore, it lays an experimental foundation for the subsequent development of prostate cancer therapeutic drugs or adjunctive intervention protocols based on ursolic acid derivatives.

## Figures and Tables

**Table 1:** R is selected from triphenylphosphine, diphenylcyclohexylphosphine, tri-p-tolylphosphine, tris(4-methoxyphenyl)phosphine, tris(4-chlorophenyl)phosphine, tris(3,5-xylyl)phosphine, and tris(o-methoxyphenyl)phosphine.

R	Compound	R	Compound
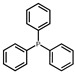	**6**, **13**, **20**, **27**, **34**	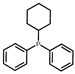	**7**, **14**, **21**, **28**, **35**
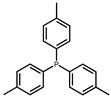	**8**, **15**, **22**, **29**, **36**	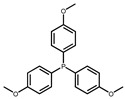	**9**, **16**, **23**, **30**, **37**
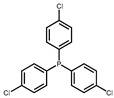	**10**, **17**, **24**, **31**, **38**	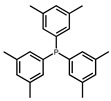	**11**, **18**, **25**, **32**, **39**
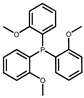	**12**, **19**, **26**, **33**, **40**		

**Table 2:** IC_50_ values of ursolic acid derivatives against three human tumor cell lines.

		IC_50_ (μM)				IC_50_ (μM)	
Compound	A549	PC3M	HepG2	Compound	A549	PC3M	HepG2
**1**	50.54 ± 11.23	45.96 ± 4.24	40.53 ± 10.35	**21**	4.72 ± 1.23	3.554 ± 0.87	10.25 ± 4.32
**2**	59.824 ± 7.25	≥200	≥200	**22**	4.11 ± 1.61	3.001 ± 0.96	9.34 ± 3.39
**3**	77.28 ± 9.29	46.16 ± 11.21	62.75 ± 9.32	**23**	4.534 ± 1.01	3.446 ± 0.83	10.69 ± 6.26
**4**	≥200	≥200	66.11 ± 11.68	**24**	2.931 ± 0.62	1.835 ± 0.94	8.497 ± 2.37
**5**	≥200	≥200	≥200	**25**	2.32 ± 0.73	1.302 ± 0.13	6.08 ± 2.42
**6**	6.524 ± 1.26	6.43 ± 0.63	7.89 ± 2.37	**26**	3.635 ± 1.22	2.794 ± 0.28	7.11 ± 2.32
**7**	17.48 ± 6.33	12.1 ± 4.23	46.16 ± 16.53	**27**	13.67 ± 3.37	7.13 ± 1.53	19.58 ± 5.36
**8**	7.92 ± 3.2	5.73 ± 1.25	32.51 ± 10.87	**28**	9.56 ± 4.26	6.83 ± 2.11	21.27 ± 8.27
**9**	6.72 ± 1.21	5.12 ± 1.32	9.89 ± 3.62	**29**	12.8 ± 1.58	9.561 ± 2.91	36.38 ± 5.88
**10**	7.015 ± 2.36	5.541 ± 1.25	7.196 ± 0.25	**30**	8.211 ± 2.43	10.2 ± 2.26	46.43 ± 18.31
**11**	5.182 ± 1.11	3.559 ± 0.22	6.532 ± 1.26	**31**	7.74 ± 1.79	9.22 ± 0.12	32.89 ± 10.56
**12**	6.23 ± 0.68	4.483 ± 0.54	9.124 ± 2.87	**32**	5.81 ± 0.63	2.485 ± 0.11	10.82 ± 0.56
**13**	7.376 ± 1.21	5.359 ± 1.63	6.643 ± 0.93	**33**	7.93 ± 0.17	3.724 ± 0.66	12.48 ± 4.28
**14**	6.582 ± 1.24	8.221 ± 3.38	10.98 ± 5.25	**34**	32.81 ± 8.28	21.04 ± 9.37	37.4 ± 8.53
**15**	1.33 ± 0.11	3.075 ± 1.62	7.641 ± 1.31	**35**	32.63 ± 3.29	24.54 ± 5.89	43.73 ± 6.38
**16**	2.931 ± 0.88	2.835 ± 0.26	8.497 ± 2.33	**36**	23.951 ± 6.52	18.563 ± 4.77	32.18 ± 15.43
**17**	4.271 ± 1.72	3.579 ± 0.25	8.75 ± 4.25	**37**	19.68 ± 6.23	14.25 ± 2.69	26.82 ± 4.37
**18**	2.82 ± 0.33	1.582 ± 0.13	4.532 ± 0.12	**38**	20.35 ± 5.53	16.71 ± 3.54	34.38 ± 15.28
**19**	3.74 ± 1.11	3.913 ± 0.82	8.653 ± 0.78	**39**	16.32 ± 2.84	7.483 ± 0.86	17.18 ± 1.87
**20**	3.723 ± 0.83	2.835 ± 0.18	7.58 ± 0.56	**40**	14.2 ± 1.36	8.6 ± 1.17	19.4 ± 4.62
DTX	8.63 ± 1.37	3.432 ± 0.827	13.25 ± 4.52	UA	13.55 ± 2.73	37.36 ± 5.14	12.33 ± 4.29

## Data Availability

The original contributions presented in this study are included in the article/Supplementary Materials. Further inquiries can be directed to the corresponding authors.

## Supplementary Materials

The following supporting information can be downloaded at: https://www.mdpi.com/article/10.3390/ph19050726/s1.

## Abbreviations

UA	Ursolic acid
PCa	Prostate cancer
NCHS	U.S. National Center for Health Statistics
ROS	Reactive oxygen species
ATP	Adenosine triphosphate
F-actin	Filamentous actin
G-actin	Globular actin
FGF	Fibroblast growth factor
FGFR1	Fibroblast growth factor receptor 1
MAPK	Mitogen-activated protein kinase
PI3K	Phosphatidylinositol 3-kinase
AKT	Protein kinase B
PIP4K2	Phosphatidylinositol-5-phosphate 4-kinase type 2
PIP2	Phosphatidylinositol 4,5-bisphosphate
GSN	Gelsolin
NMR	Nuclear magnetic resonance
^1^H NMR	Proton nuclear magnetic resonance
^13^C NMR	Carbon-13 nuclear magnetic resonance
TLC	Thin-layer chromatography
CCK-8	Cell Counting Kit-8
EdU	5-ethynyl-2′-deoxyuridine
DMSO	Dimethyl sulfoxide
OD	Optical density
IC_50_	Half-maximal inhibitory concentration
PBS	Phosphate-buffered saline
DMEM	Dulbecco’s Modified Eagle Medium
FBS	Fetal bovine serum
SOD	Superoxide dismutase
MDA	Malondialdehyde
RIPA	Radio Immunoprecipitation Assay
PVDF	Polyvinylidene fluoride
Bcl-2	B-cell lymphoma-2
Bax	Bcl-2-associated X protein
GAPDH	Glyceraldehyde-3-phosphate dehydrogenase
ΔΨm	Mitochondrial membrane potential
JC-1	5,5′,6,6′-tetrachloro-1,1′,3,3′-tetraethylbenzimidazolylcarbocyanine iodide
SDS-PAGE	Sodium dodecyl sulfate-polyacrylamide gel electrophoresis
BCA	Bicinchoninic acid
TBST	Tris-buffered saline with Tween-20
ECL	Enhanced chemiluminescence
PDB	Protein Data Bank
SPF	Specific-pathogen-free
IACUC	Institutional Animal Care and Use Committee
H&E	Hematoxylin-eosin
TUNEL	Terminal deoxynucleotidyl transferase dUTP nick end labeling
SD	Standard deviation
ANOVA	One-way analysis of variance
FC	Fold change
FDR	False discovery rate
GO	Gene Ontology
KEGG	Kyoto Encyclopedia of Genes and Genomes
PPI	Protein–protein interaction
MOMP	Mitochondrial outer membrane permeabilization
DTX	Docetaxel
ECM	Extracellular matrix
PI	Propidium iodide
FITC	Fluorescein isothiocyanate
