# Supplementary material for: Pharmacological Mechanisms of Ursolic Acid Derivative Against Prostate Cancer via Regulating Cytoskeletal Homeostasis and Apoptotic Pathways
Source: Pharmaceuticals (Basel). 2026 May 2;19(5):726. doi: 10.3390/ph19050726 (PMC13209419; doi:10.3390/ph19050726)

Supplementary Materials：

Table S1. Structural Formulas of Ursolic Acid Derivatives

Compound No.	Structure
1	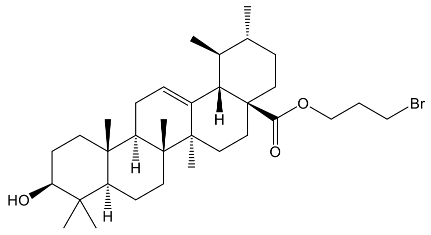
2	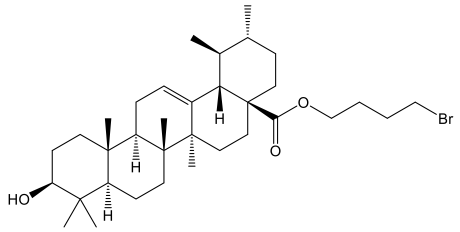
3	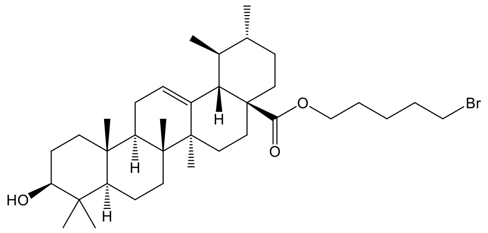
4	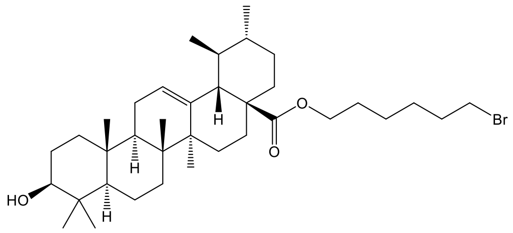
5	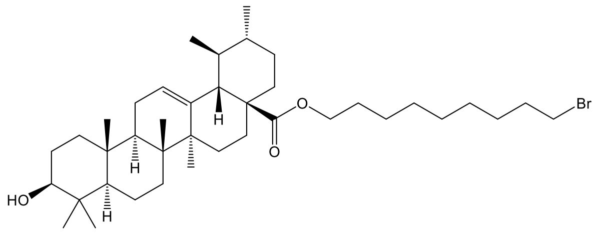
6	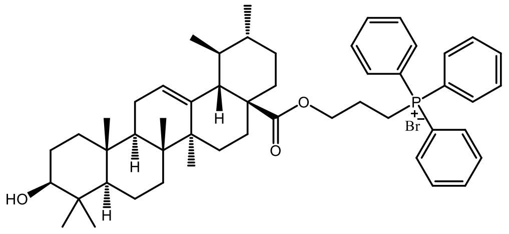

Continued from Table S1

Table S1 Continued

Compound No.	Structure
7	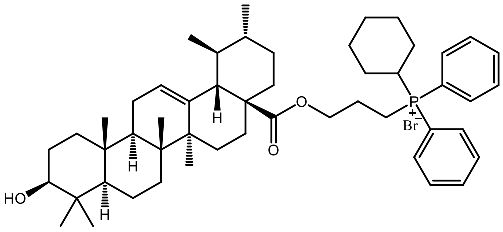
8	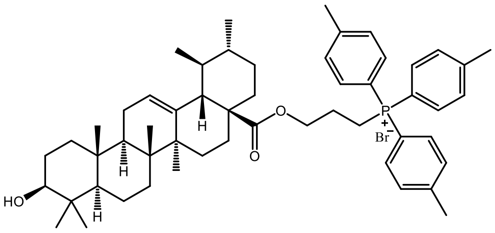
9	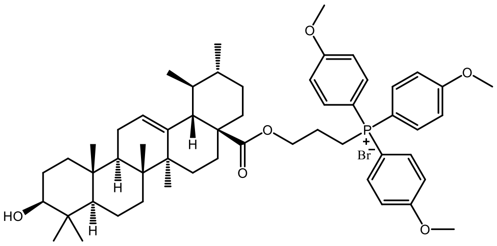
10	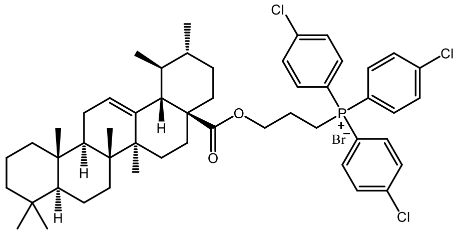
11	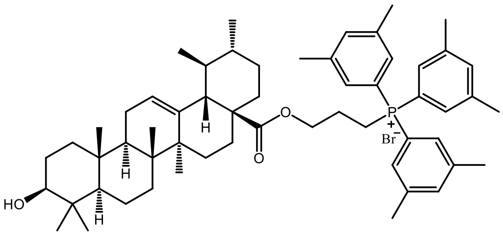
12	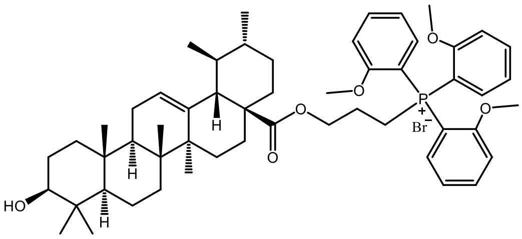

Continued from Table S1

Table S1 Continued

Compound No.	Structure
13	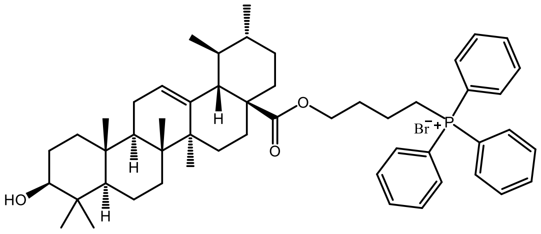
14	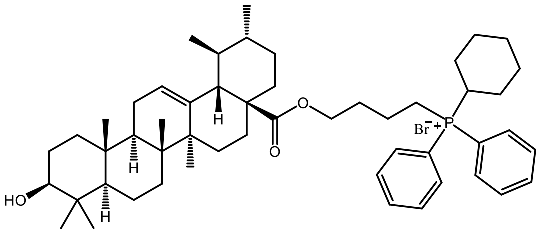
15	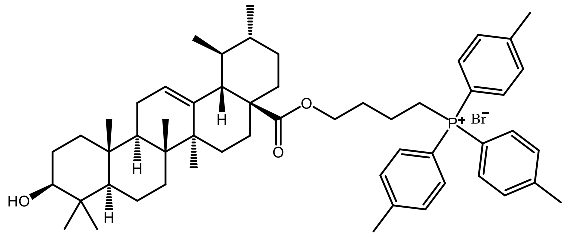
16	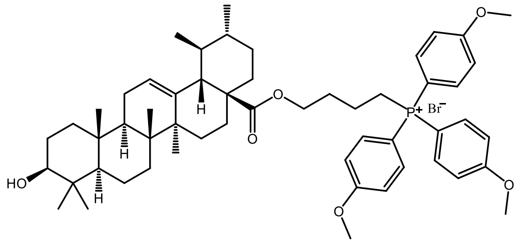
17	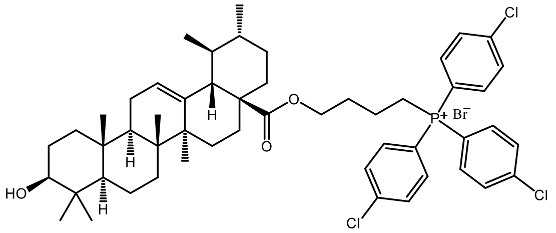
18	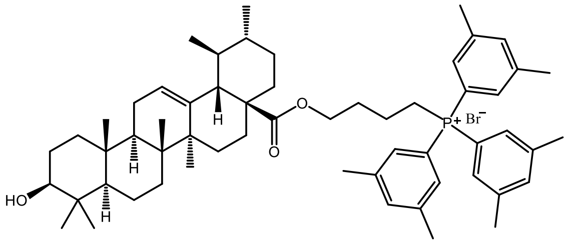

Continued from Table S1

Table S1 Continued

Compound No.	Structure
19	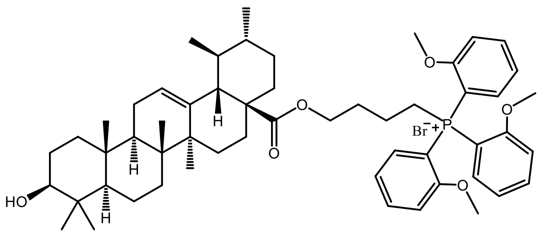
20	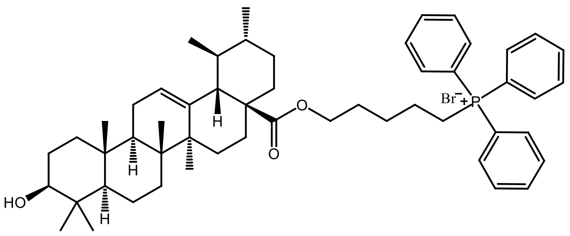
21	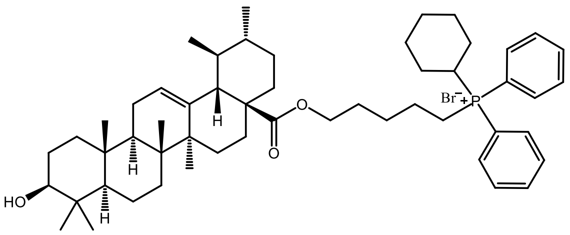
22	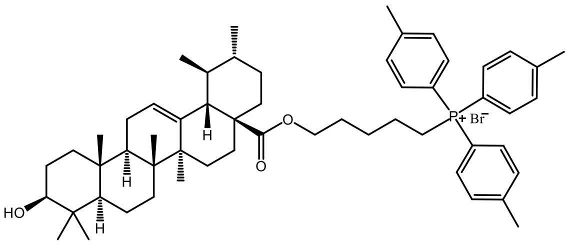
23	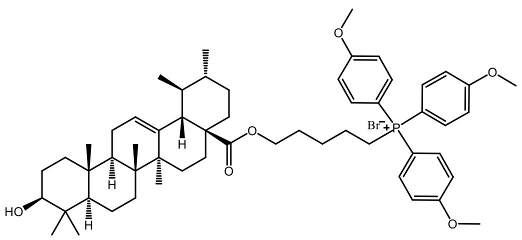
24	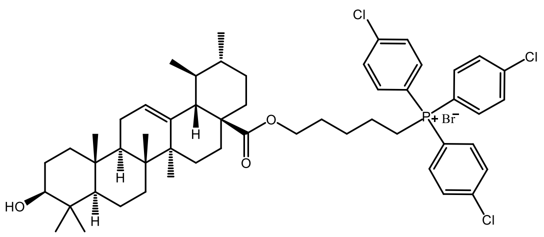

Continued from Table S1

Table S1 Continued

Compound No.	Structure
25	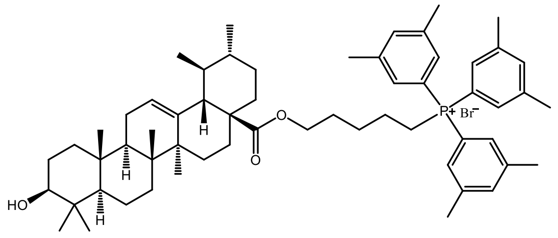
26	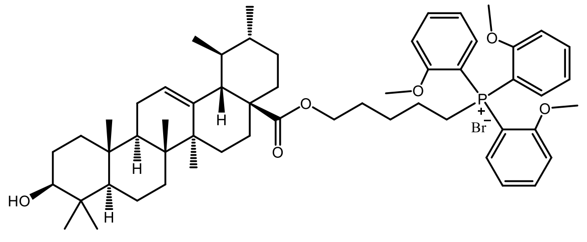
27	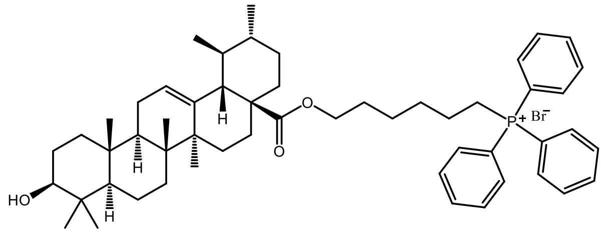
28	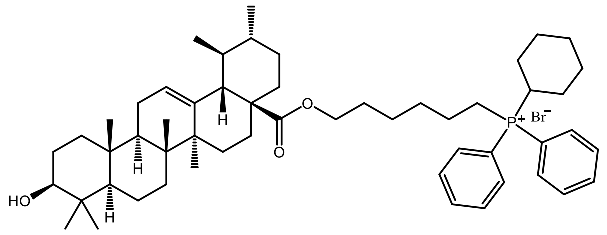
29	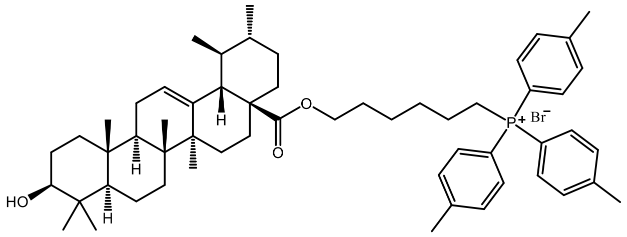
30	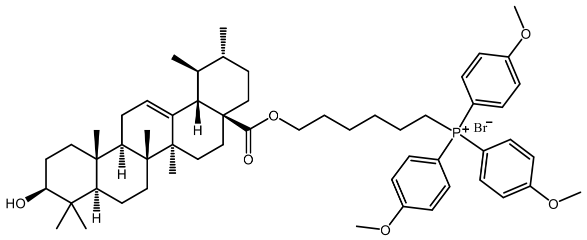

Continued from Table S1

Table S1 Continued

Compound No.	Structure
31	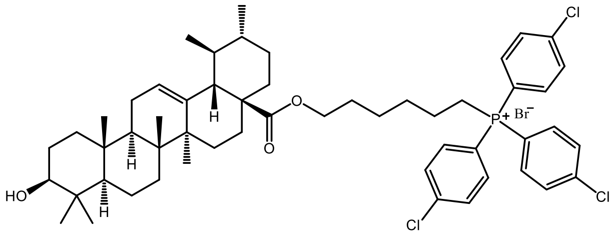
32	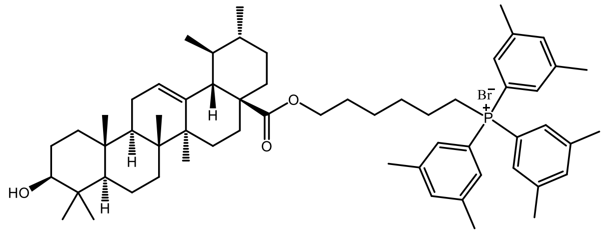
33	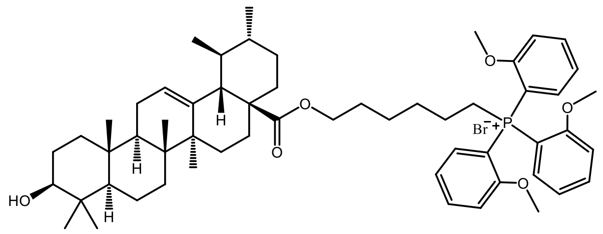
34	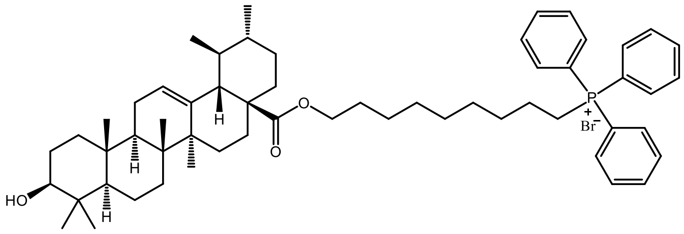
35	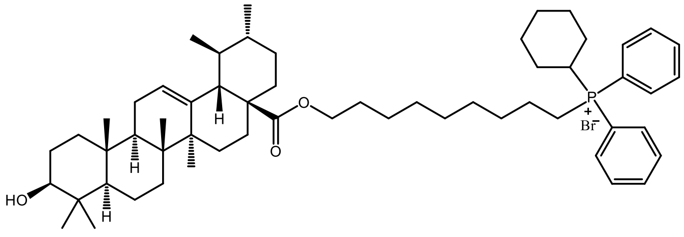
36	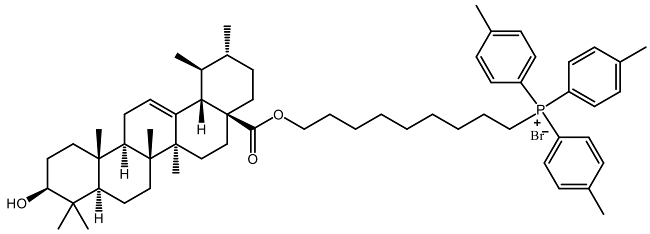

Continued from Table S1

Table S1 Continued

Compound No.	Structure
37	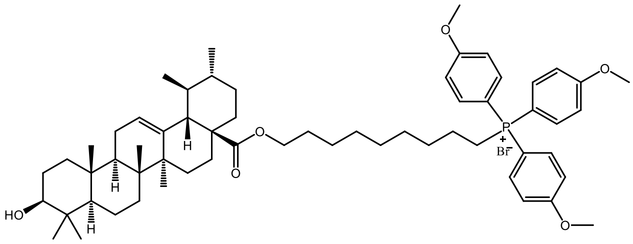
38	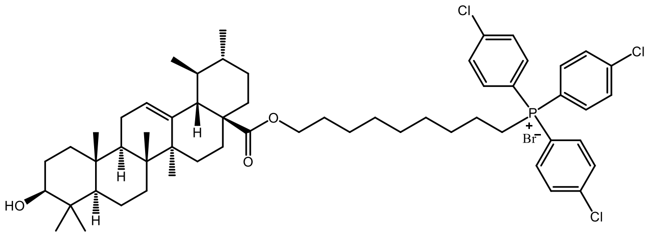
39	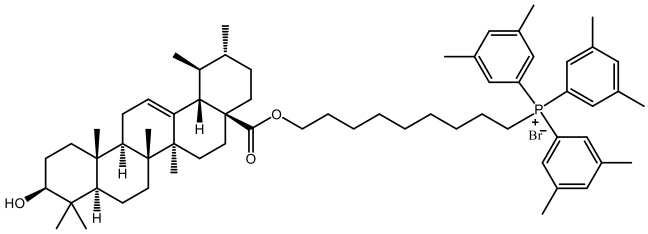
40	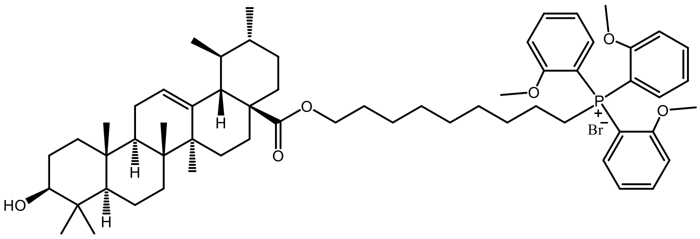

Figures S1. *^{13}C and ^{1}H NMR of compound **1**.*
Figures S2. *^{13}C and ^{1}H NMR of compound **2**.*
Figures S3. *^{13}C and ^{1}H NMR of compound **3**.*
Figures S4. *^{13}C and ^{1}H NMR of compound **4**.*
Figures S5. *^{13}C and ^{1}H NMR of compound **5**.*
Figures S6. *^{13}C and ^{1}H NMR of compound **6**.*
Figures S7. *^{13}C and ^{1}H NMR of compound 7.*
Figures S8. *^{13}C and ^{1}H NMR of compound **8**.*
Figures S9. *^{13}C and ^{1}H NMR of compound **9**.*
Figures S10. *^{13}C and ^{1}H NMR of compound **10**.*
Figures S11 *^{13}C and ^{1}H NMR of compound **11**.*
Figures S12. *^{13}C and ^{1}H NMR of compound **12**.*
Figures S13. *^{13}C and ^{1}H NMR of compound **13**.*
Figures S14. *^{13}C and ^{1}H NMR of compound **14**.*
Figures S15. *^{13}C and ^{1}H NMR of compound **15**.*
Figures S16. *^{13}C and ^{1}H NMR of compound **16**.*
Figures S17. *^{13}C and ^{1}H NMR of compound **1**7.*
Figures S18. *^{13}C and ^{1}H NMR of compound **18**.*
Figures S19. *^{13}C and ^{1}H NMR of compound **19**.*
Figures S20. *^{13}C and ^{1}H NMR of compound **20**.*
Figures S21. *^{13}C and ^{1}H NMR of compound **21**.*
Figures S22. *^{13}C and ^{1}H NMR of compound **22**.*
Figures S23. *^{13}C and ^{1}H NMR of compound **23**.*
Figures S24. *^{13}C and ^{1}H NMR of compound **24**.*
Figures S25. *^{13}C and ^{1}H NMR of compound **25**.*
Figures S26. *^{13}C and ^{1}H NMR of compound **26**.*
Figures S27. *^{13}C and ^{1}H NMR of compound **2**7.*
Figures S28. *^{13}C and ^{1}H NMR of compound **28**.*
Figures S29. *^{13}C and ^{1}H NMR of compound **29**.*
Figures S30. *^{13}C and ^{1}H NMR of compound **30**.*
Figures S31. *^{13}C and ^{1}H NMR of compound **31**.*
Figures S32. *^{13}C and ^{1}H NMR of compound **32**.*
Figures S33. *^{13}C and ^{1}H NMR of compound **33**.*
Figures S34. *^{13}C and ^{1}H NMR of compound **34**.*
Figures S35. *^{13}C and ^{1}H NMR of compound **35**.*
Figures S36. *^{13}C and ^{1}H NMR of compound **36**.*
Figures S37. *^{13}C and ^{1}H NMR of compound **3**7.*
Figures S38. *^{13}C and ^{1}H NMR of compound **38**.*
Figures S39. *^{13}C and ^{1}H NMR of compound **39**.*
Figures S40. *^{13}C and ^{1}H NMR of compound **40**.*
Figures S41. Compound 25 shows no obvious toxic effects on the heart, liver, spleen, lung, and kidney of mice. Data are presented as mean ± SD (n = 3) (Magnification × 100).

Figures S1. ^{13}*C and* ^{1}*H NMR of compound* ***1***.

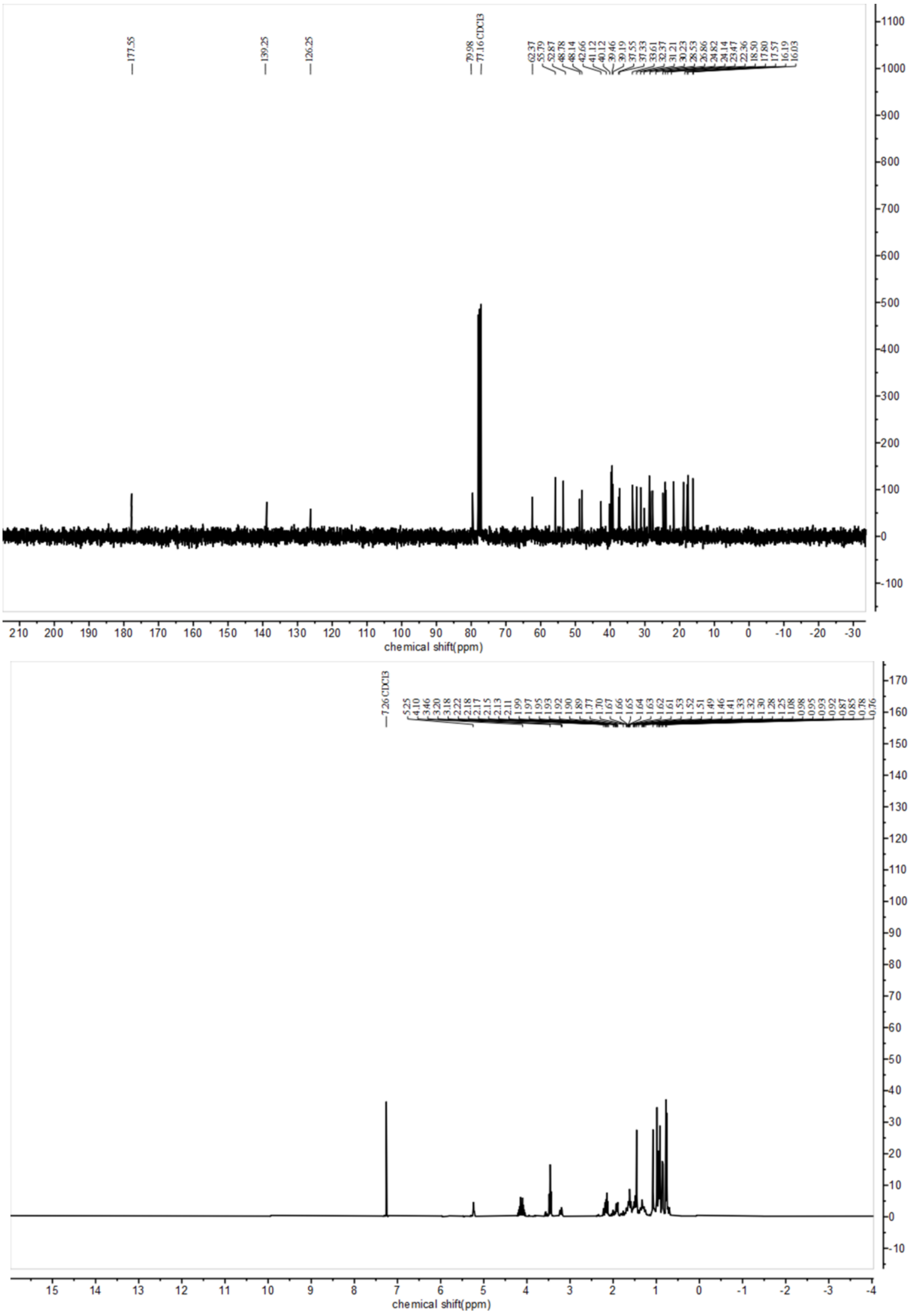

Figures S2. ^{13}C *and* ^{1}H *NMR of compound* ***2***.

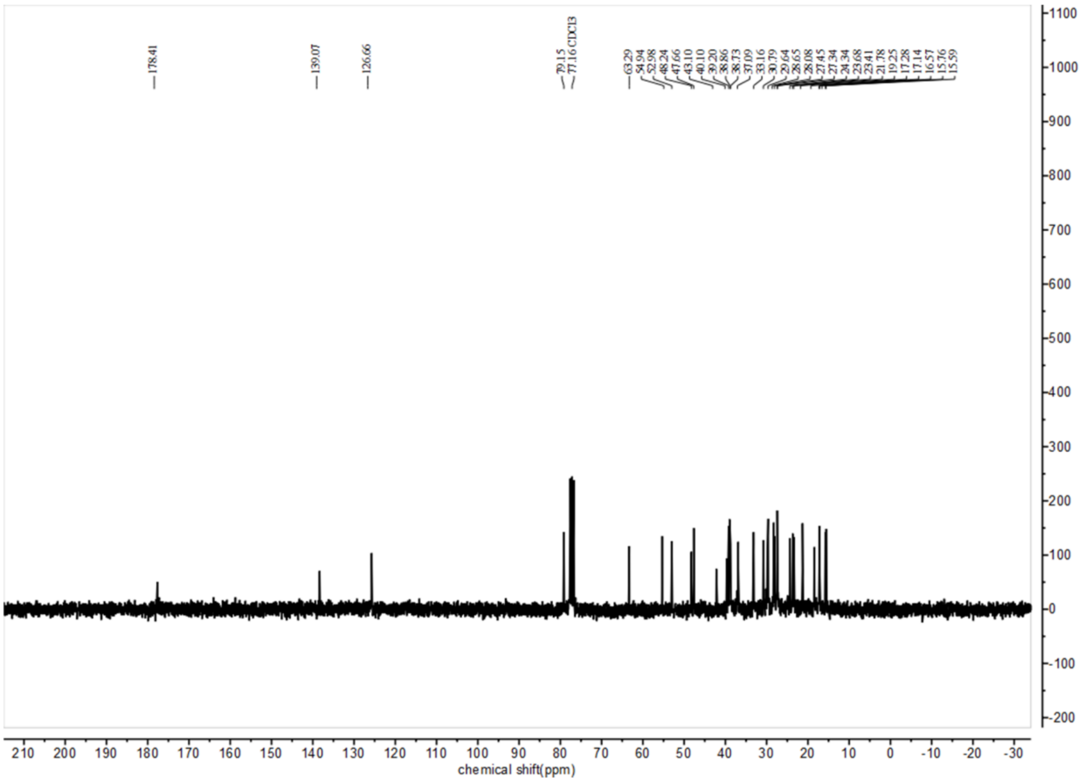

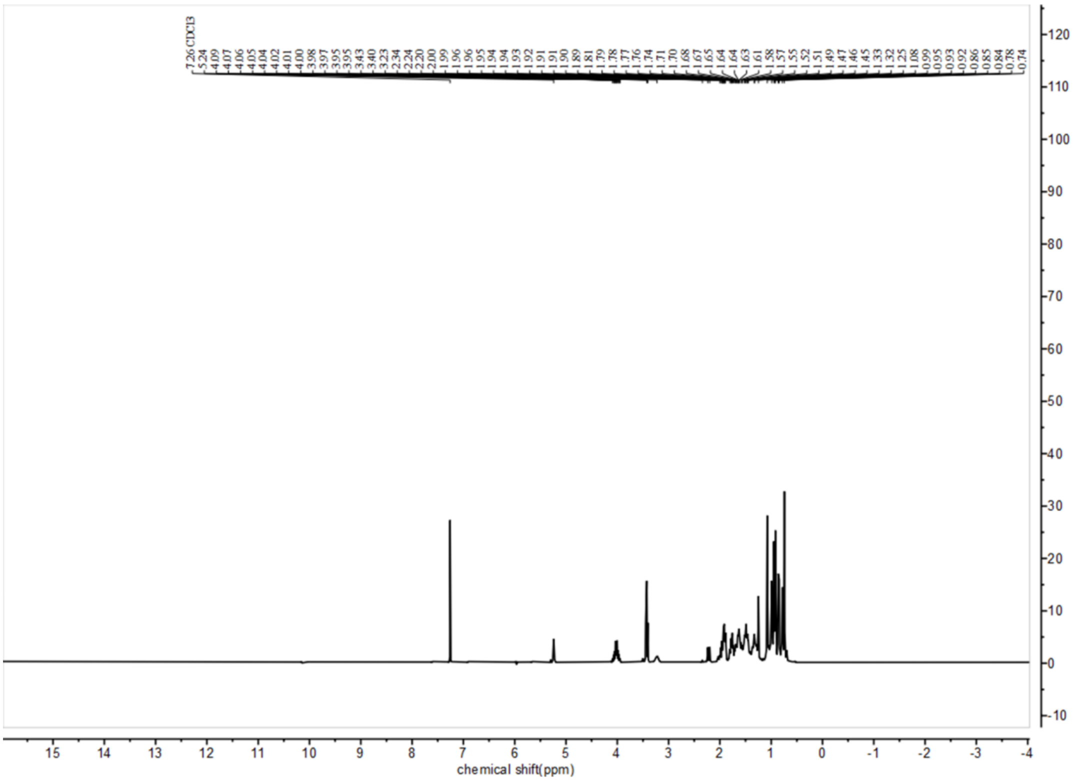

Figures S3. *^{13}C and ^{1}H NMR of compound **3***.

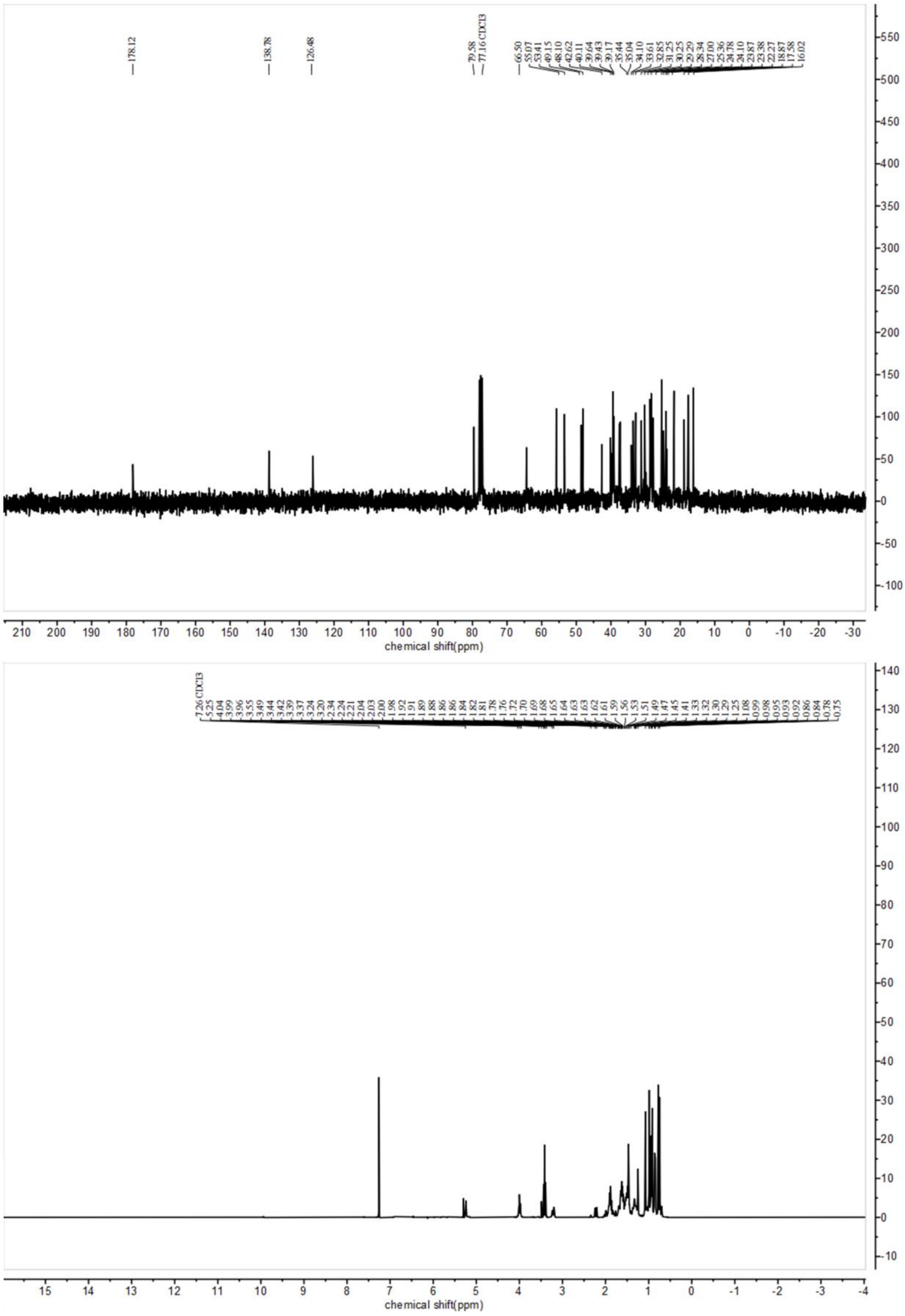

Figures S4. ^{13}C *and* ^{1}H *NMR of compound* ***4***.

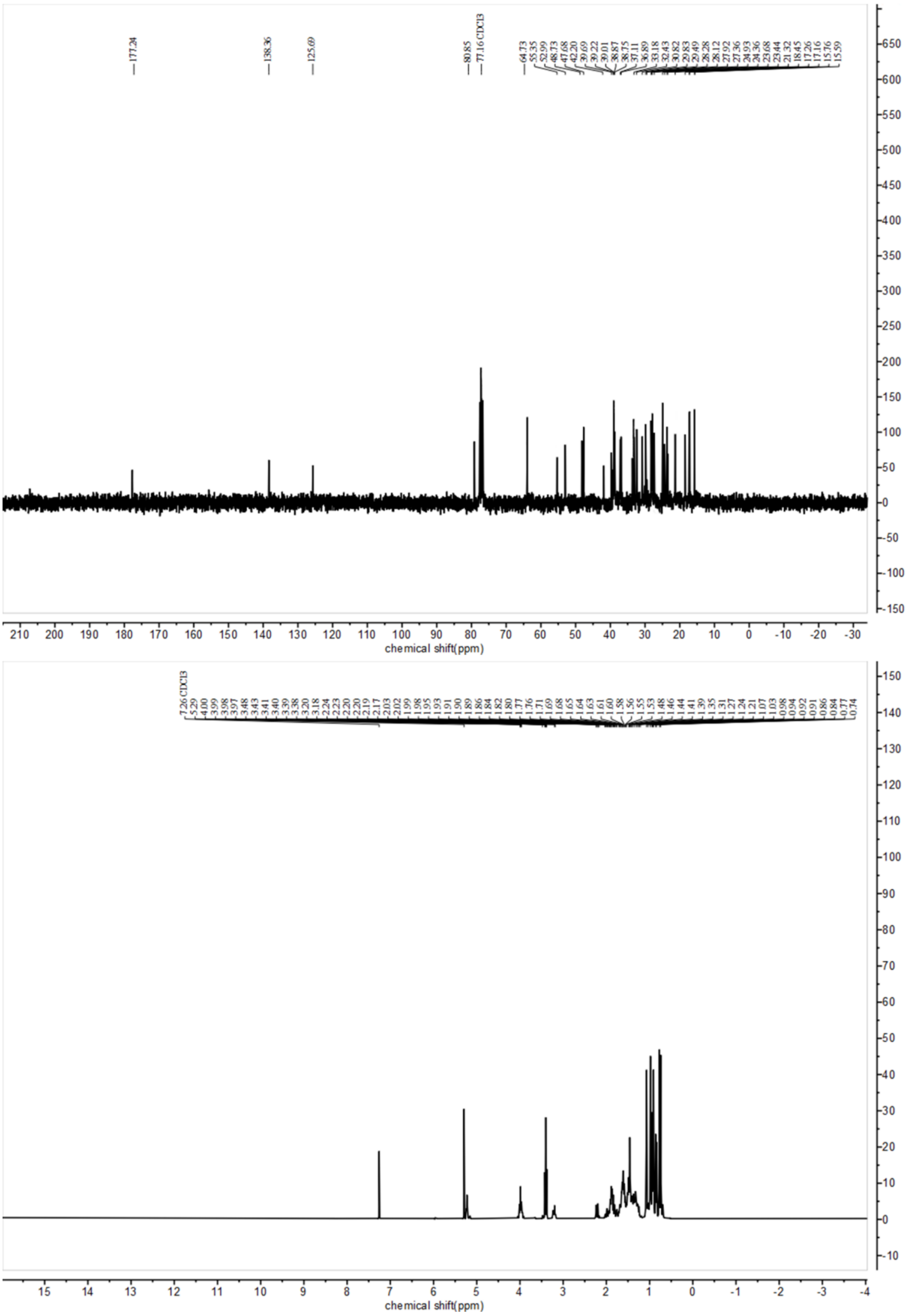

Figures S5. ^{13}C *and* ^{1}H *NMR of compound* ***5***.

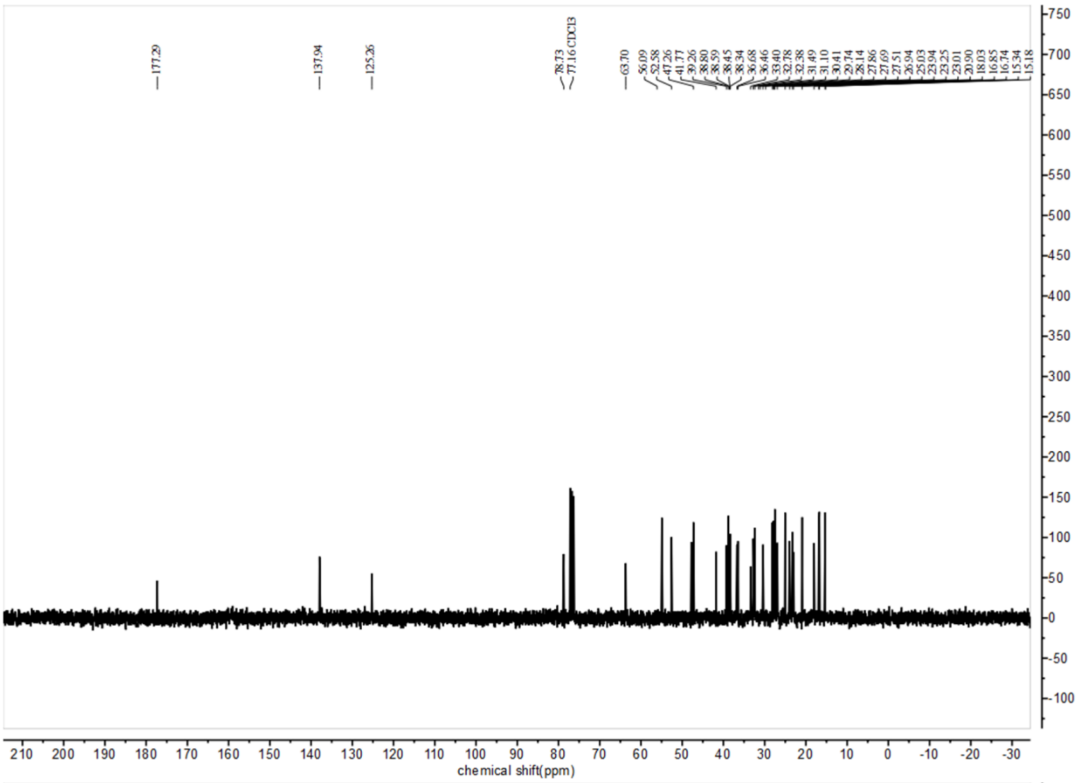

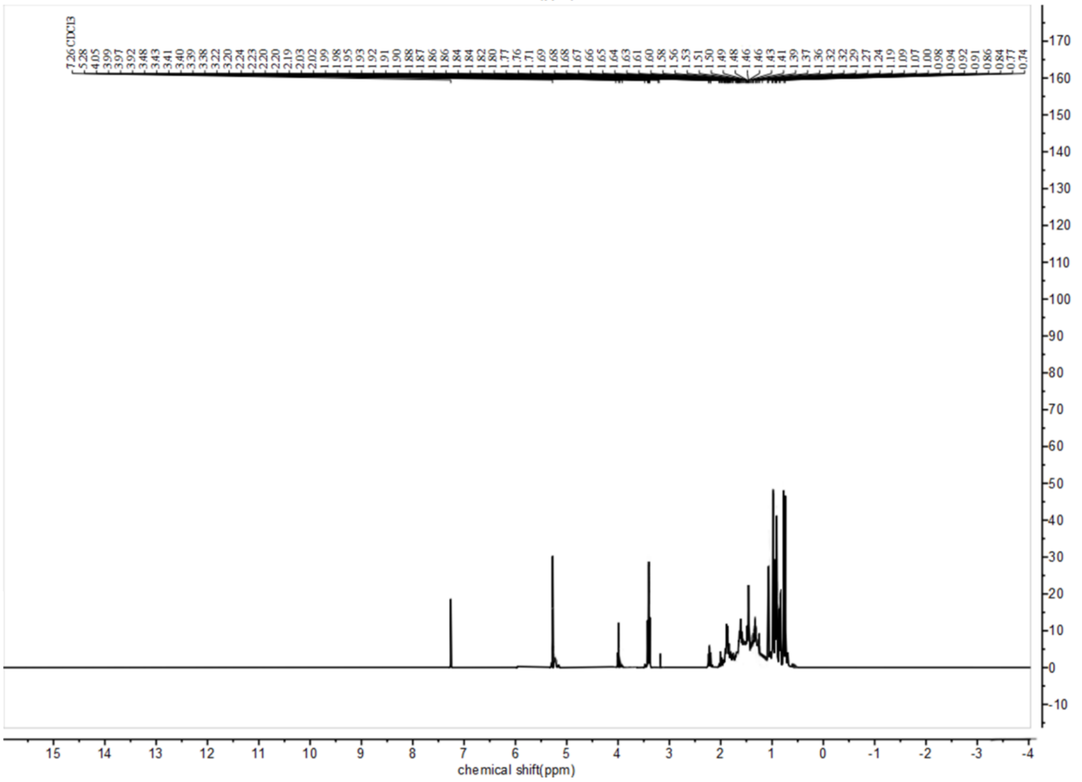

Figures S6. ^{13}C *and* ^{1}H *NMR of compound* ***6***.

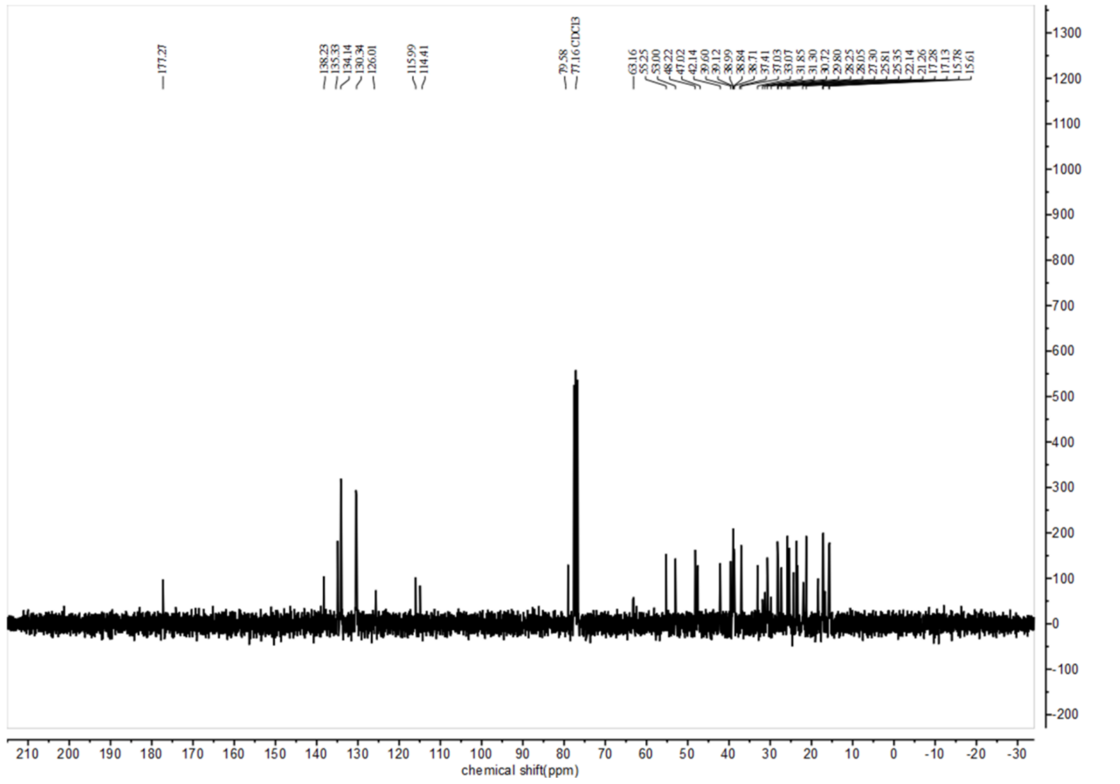

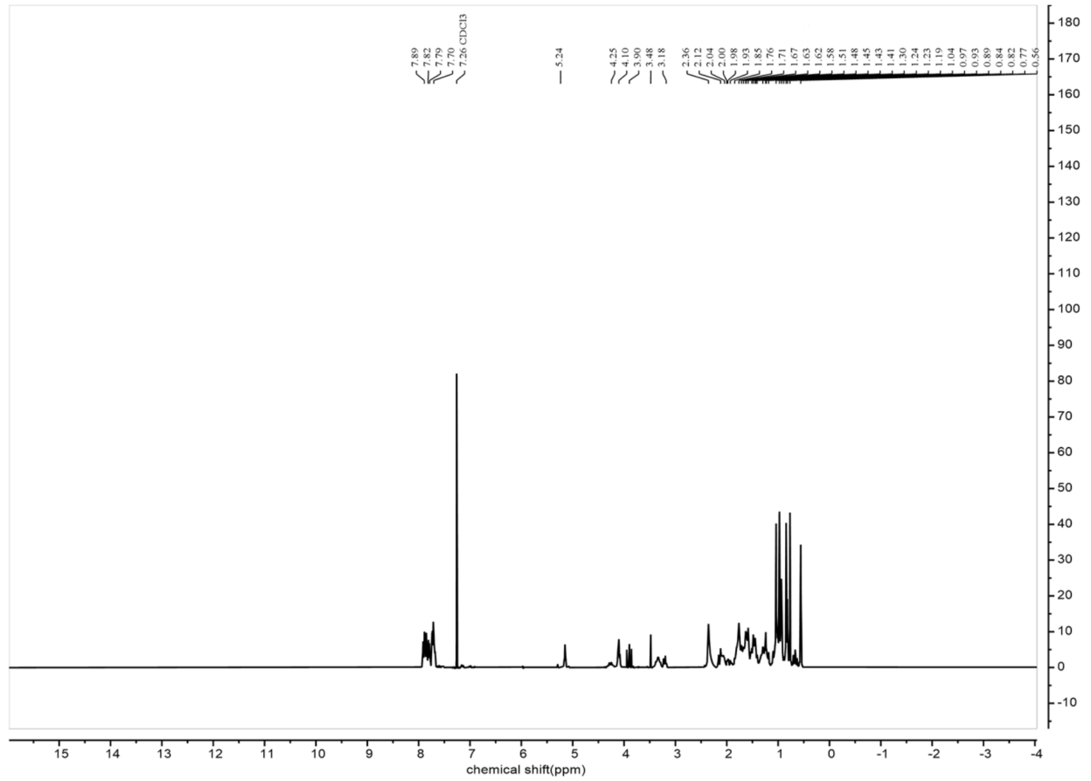

Figures S7. *^{13}C and ^{1}H NMR of compound* **7**.

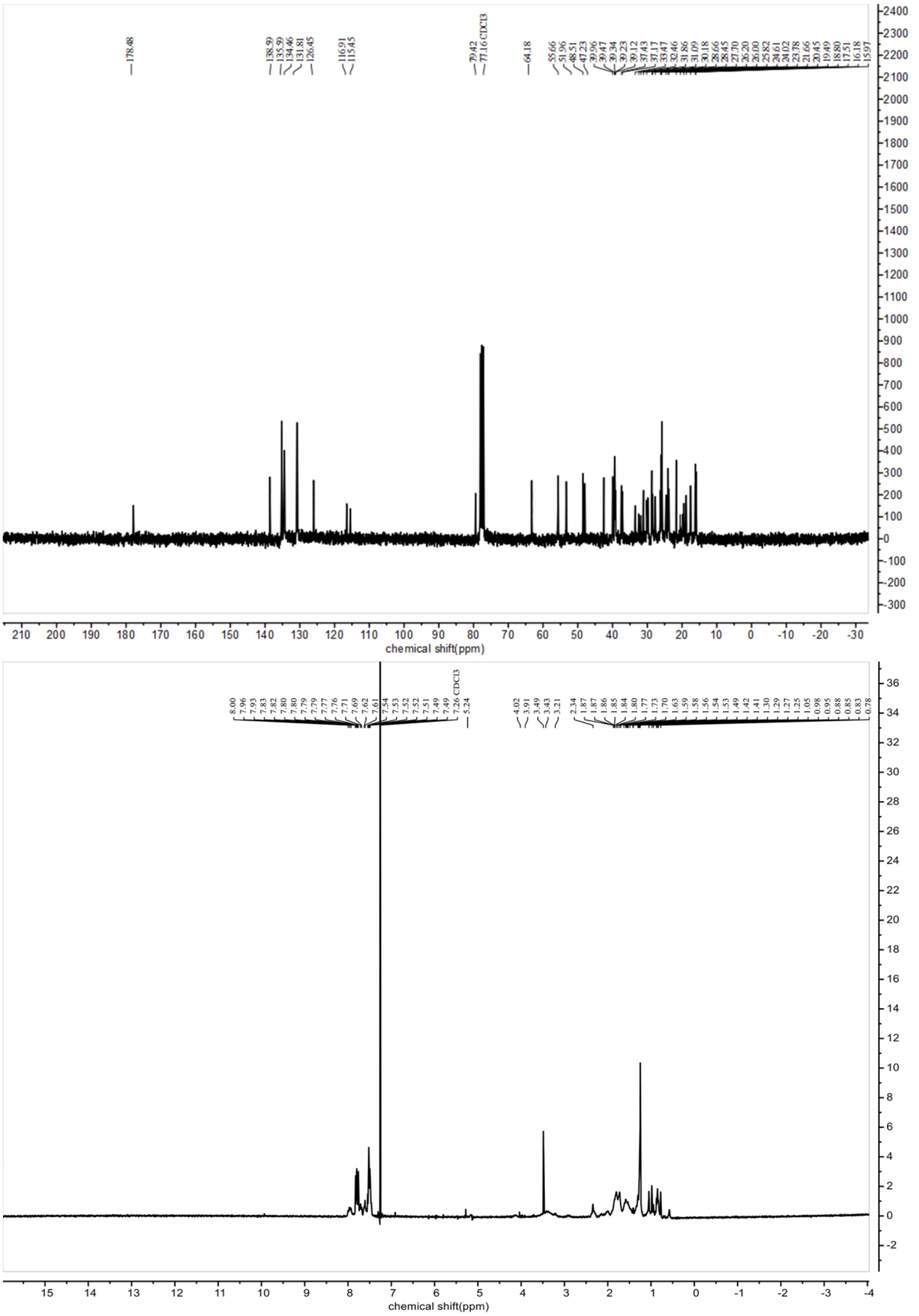

Figures S8. ^{13}C *and* ^{1}H *NMR of compound* ***8***.

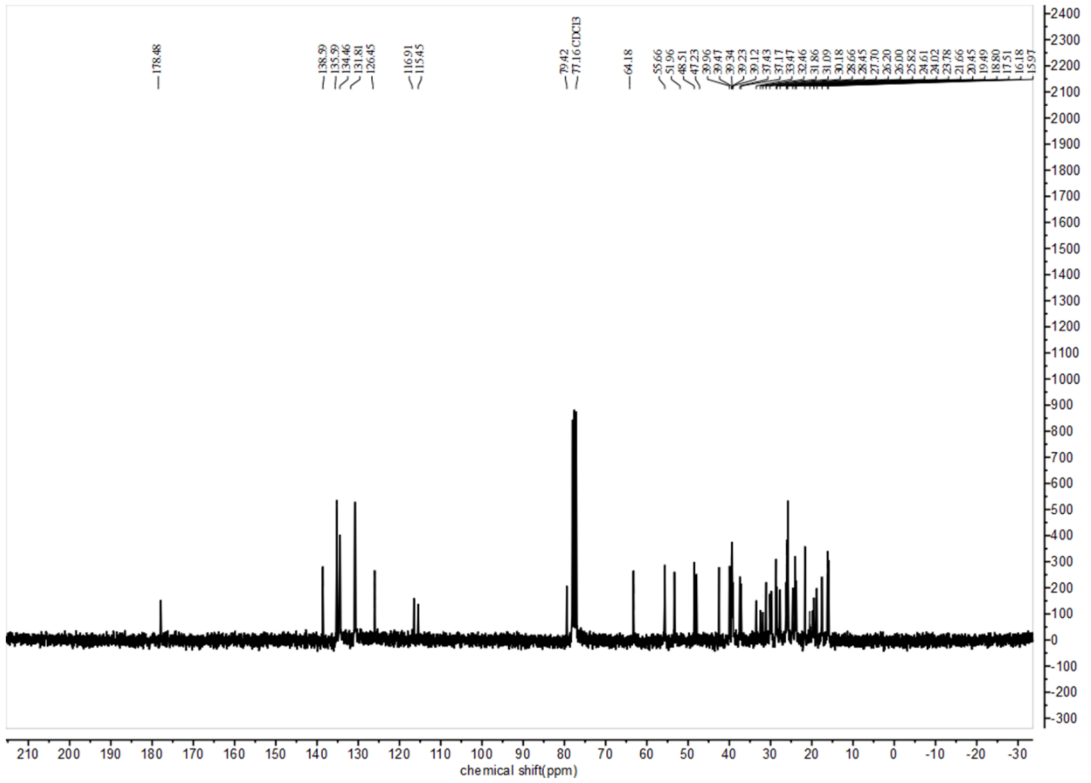

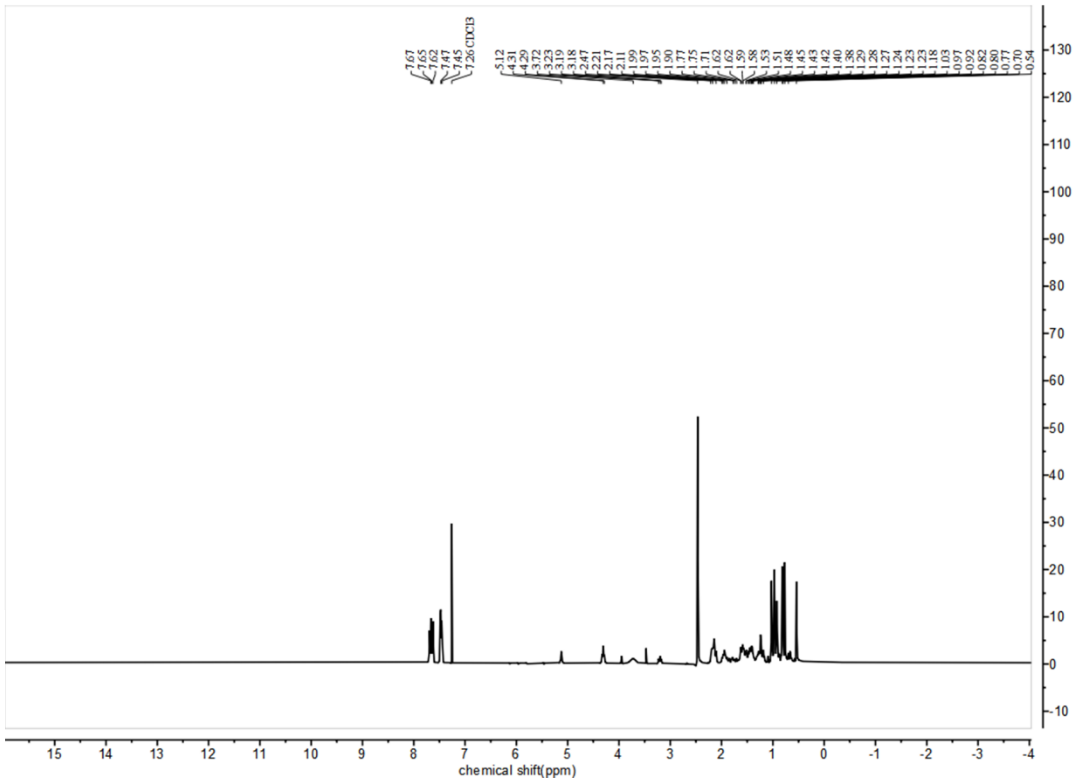

Figures S9. ^{13}C *and* ^{1}H *NMR of compound* ***9***.

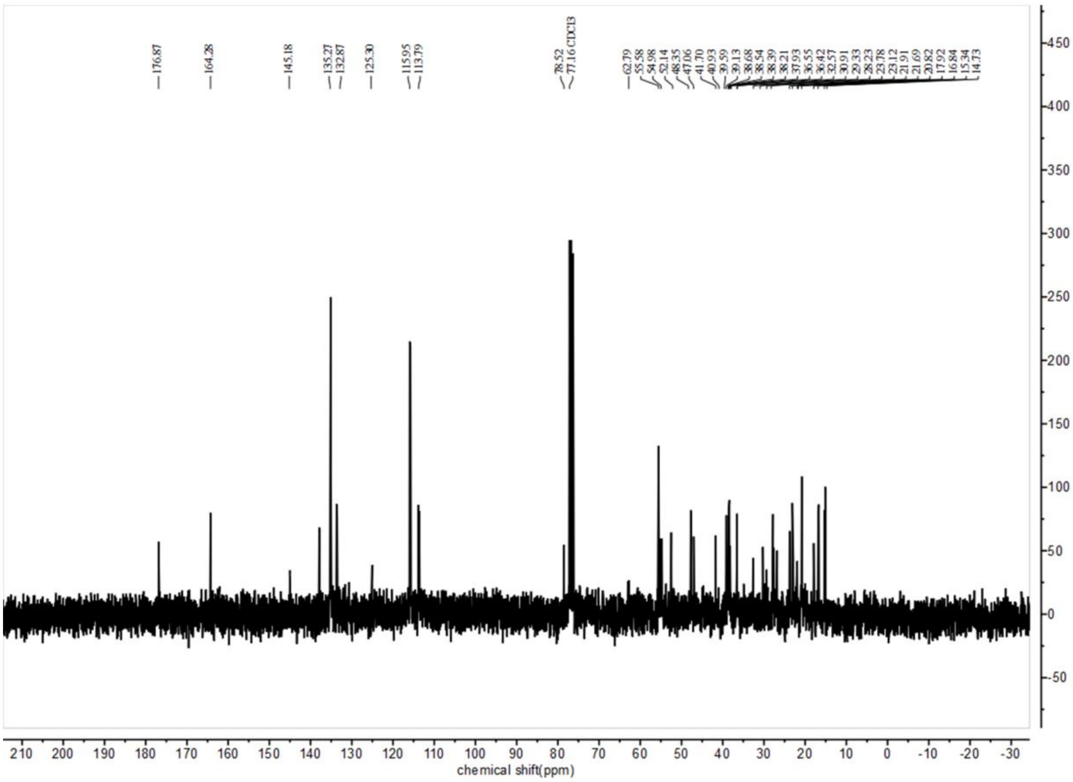

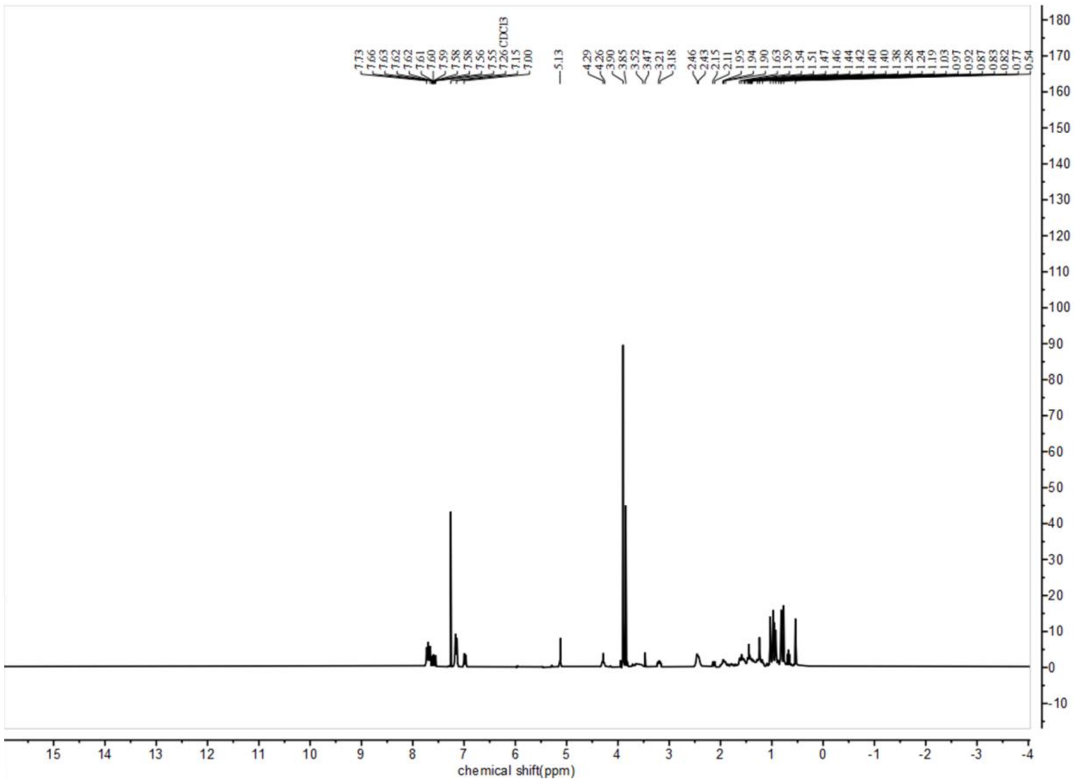

Figures S10. ^{13}C *and* ^{1}H *NMR of compound* ***10***.

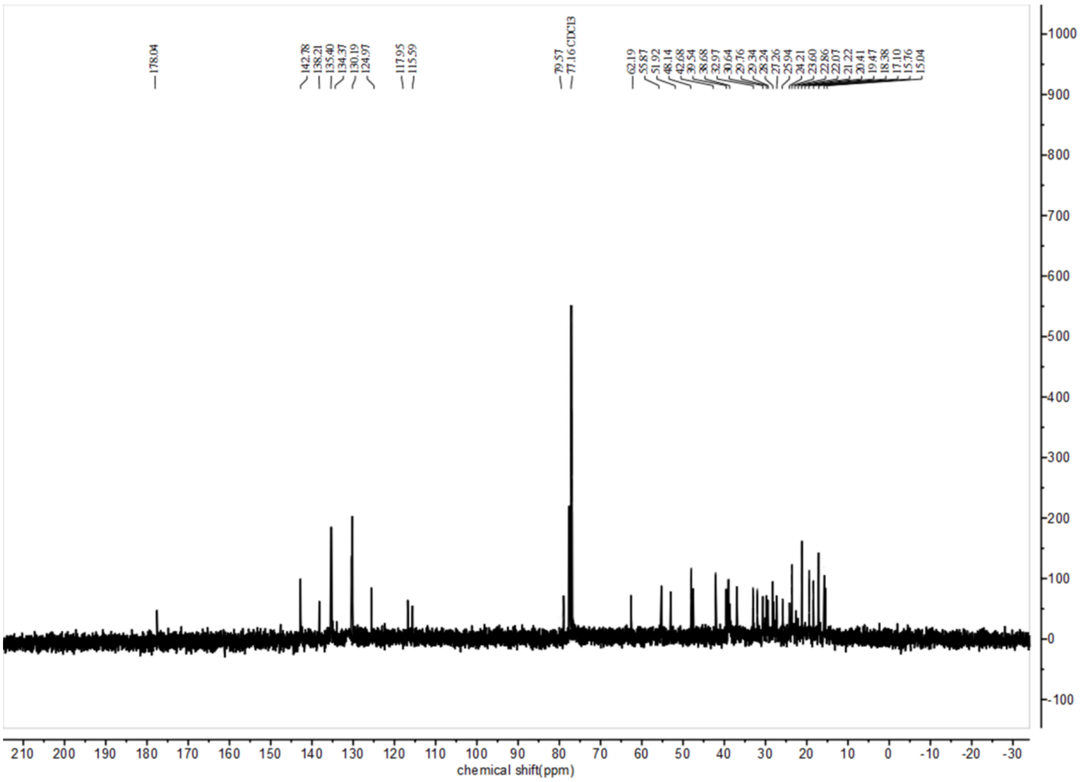

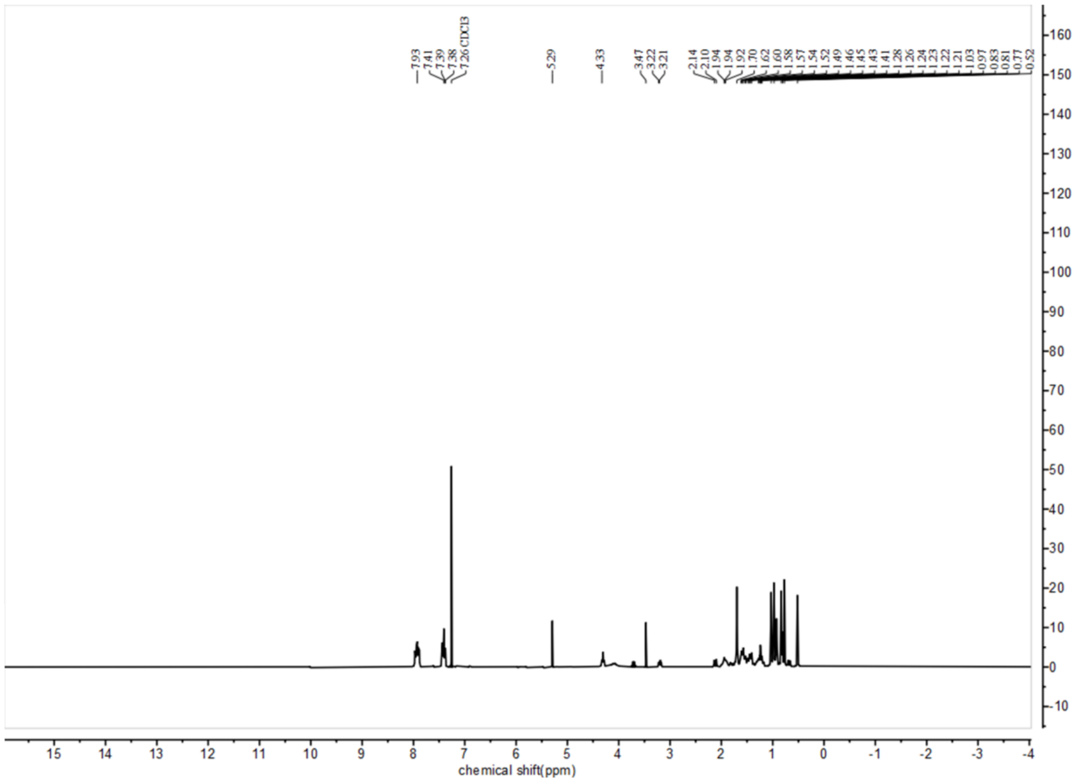

Figures S11 ^{13}C *and* ^{1}H *NMR of compound* ***11***.

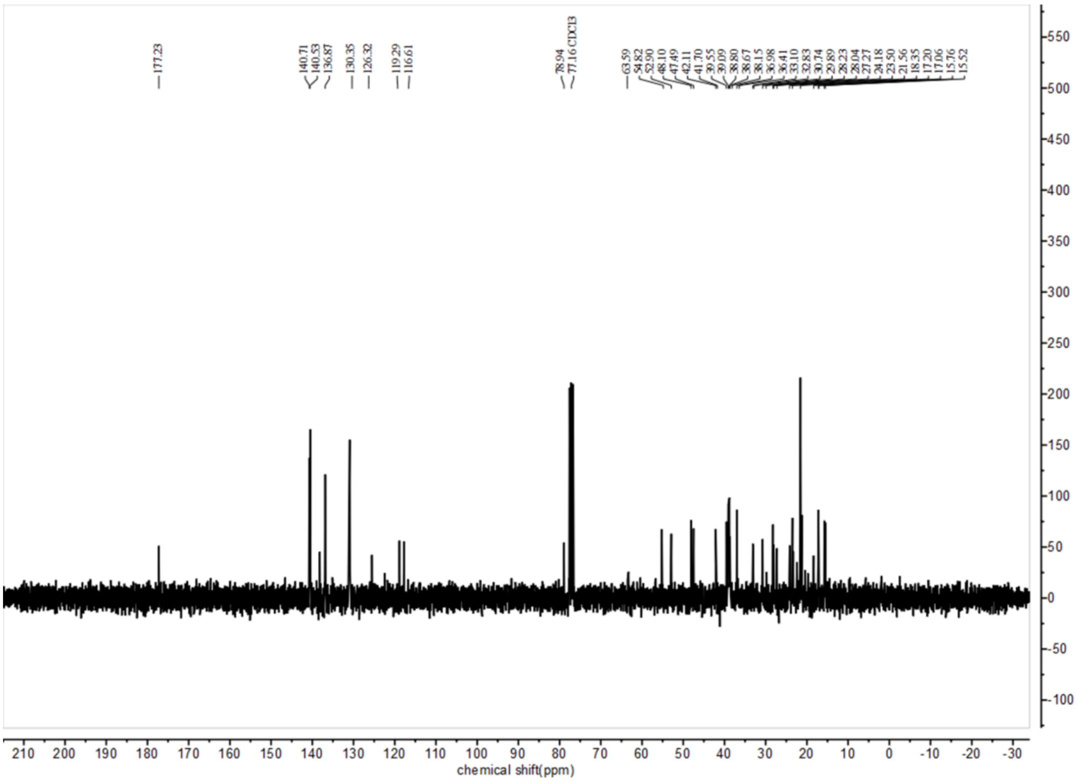

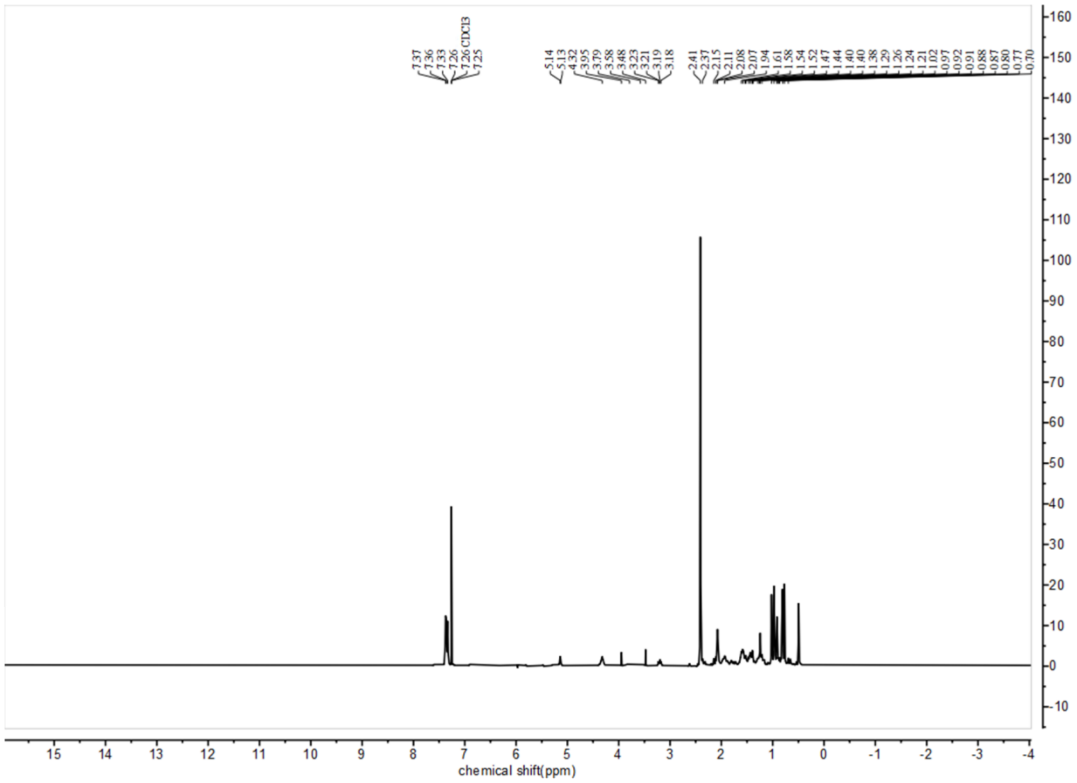

Figures S12. ^{13}*C and* ^{1}*H NMR of compound* ***12***.

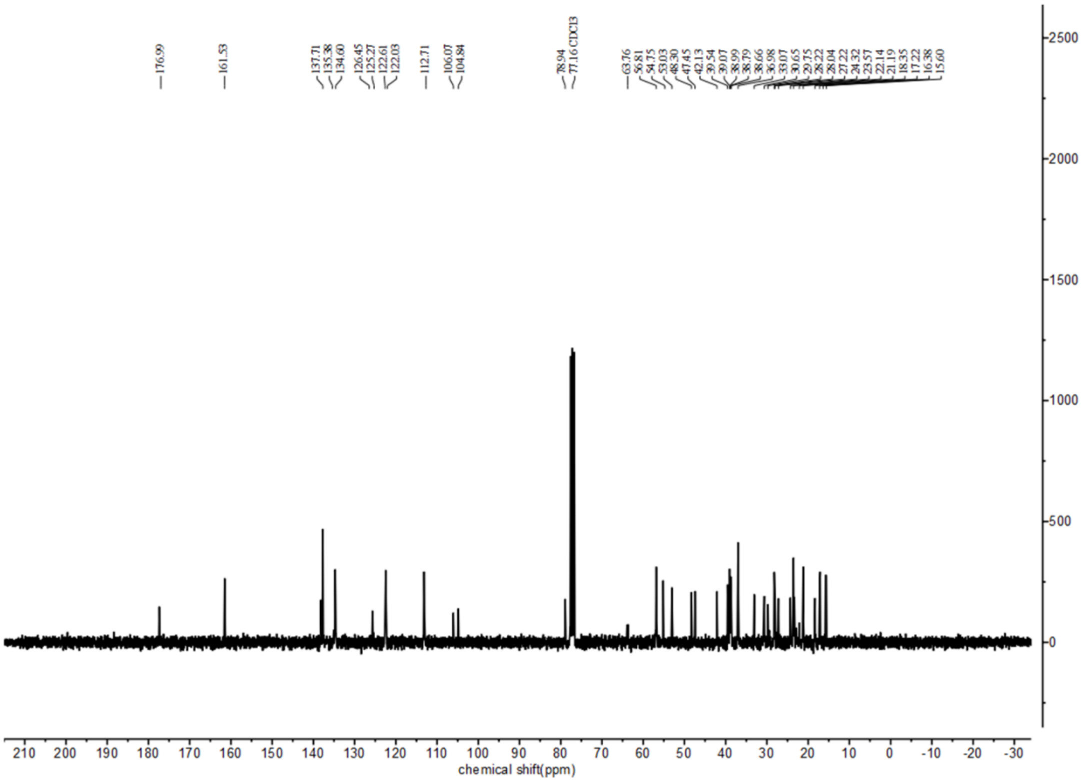

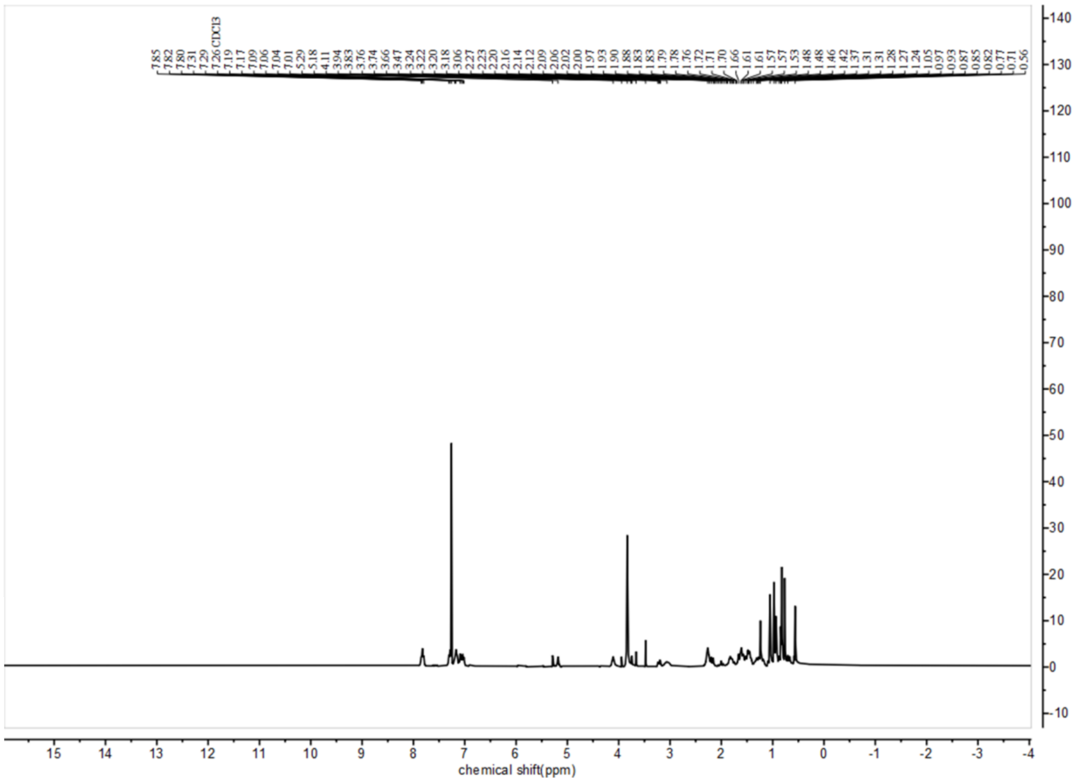

Figures S13. *^{13}C and ^{1}H NMR of compound **13***.

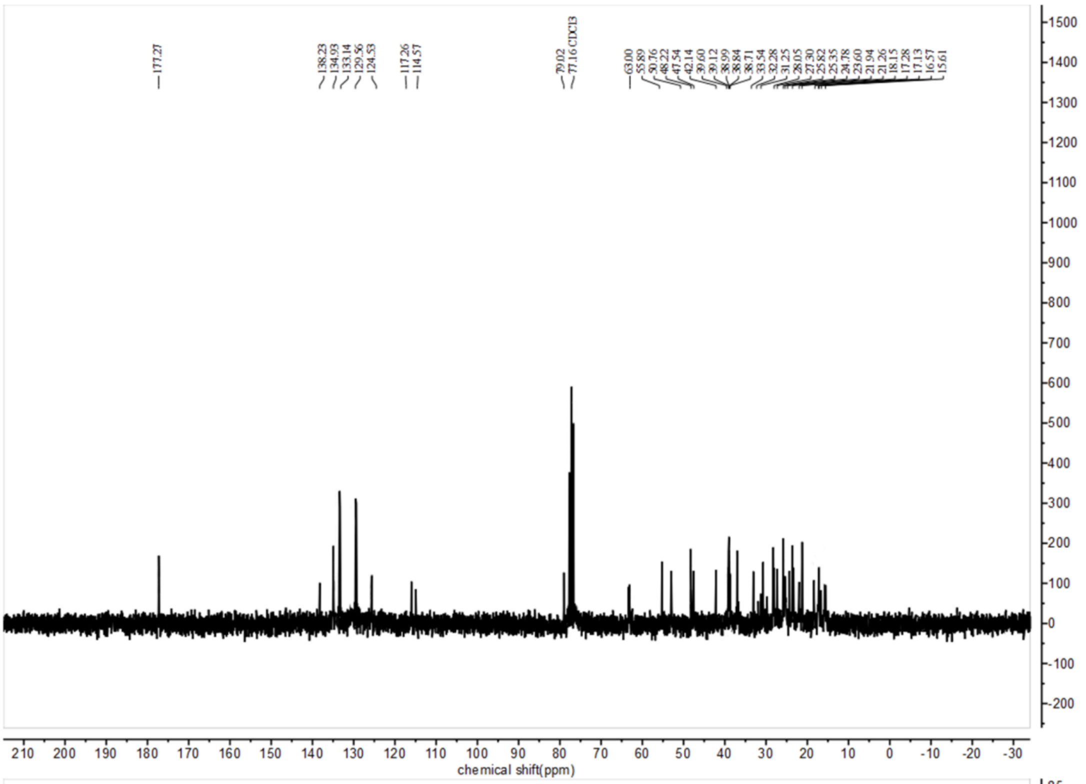

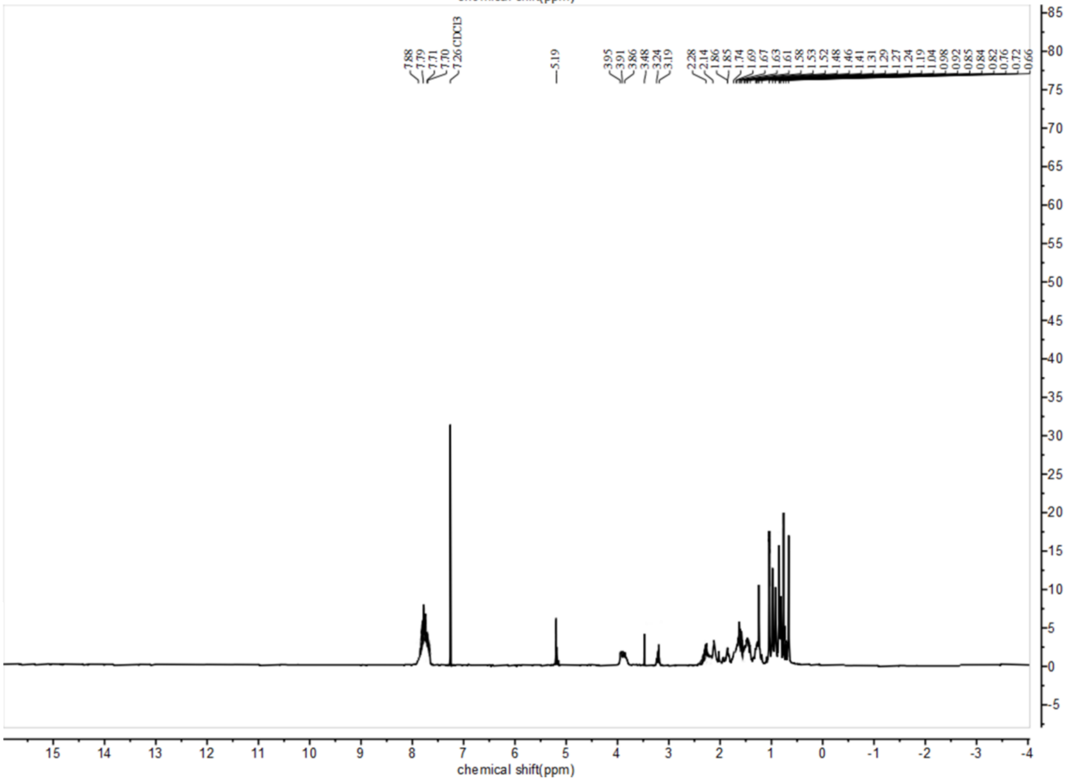

Figures S14. ^{13}C *and* ^{1}H *NMR of compound* ***14***.

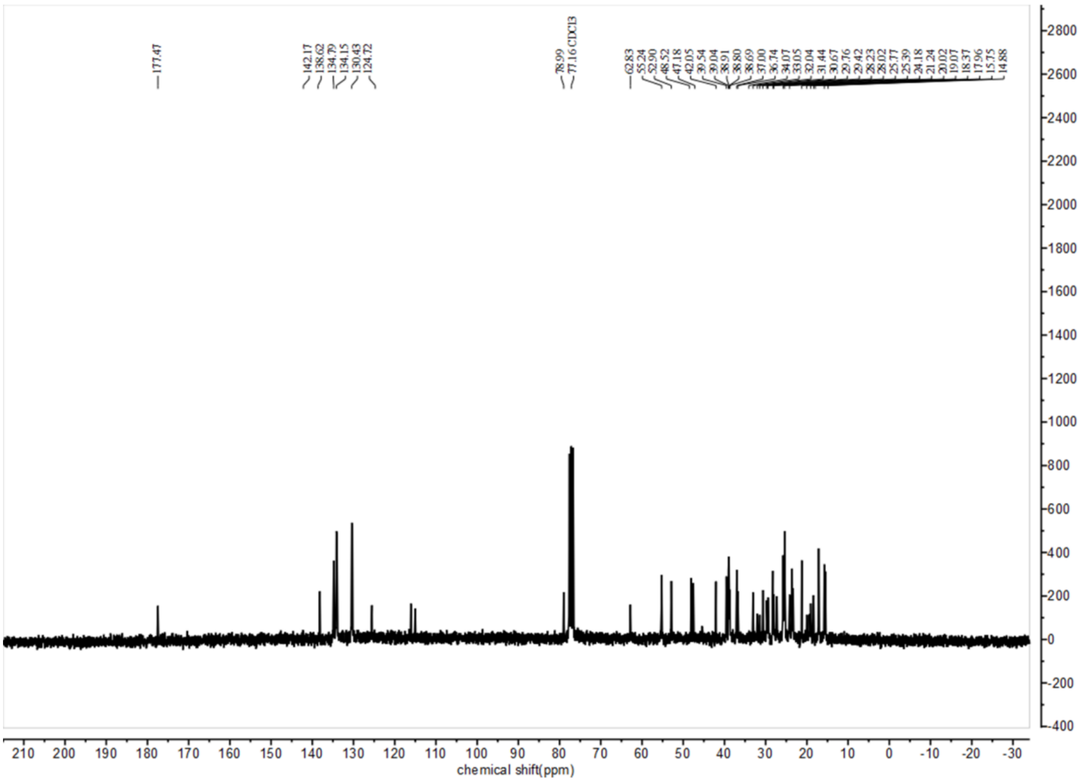

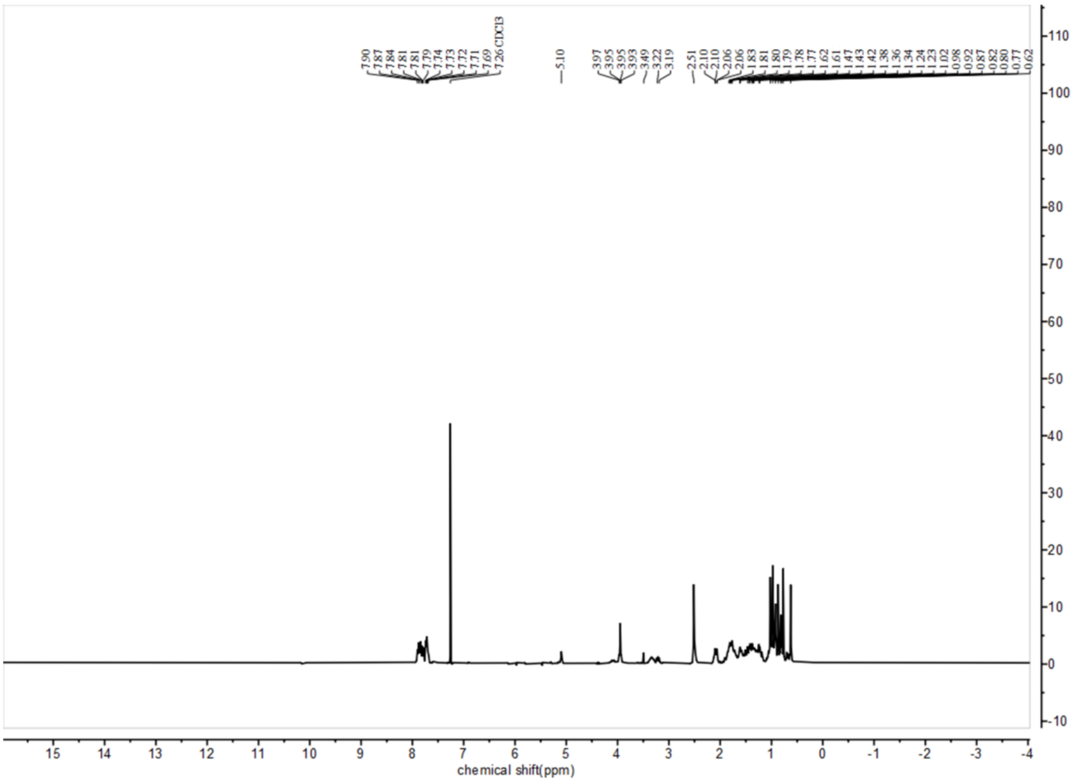

Figures S15. ^{13}C *and* ^{1}H *NMR of compound* ***15***.

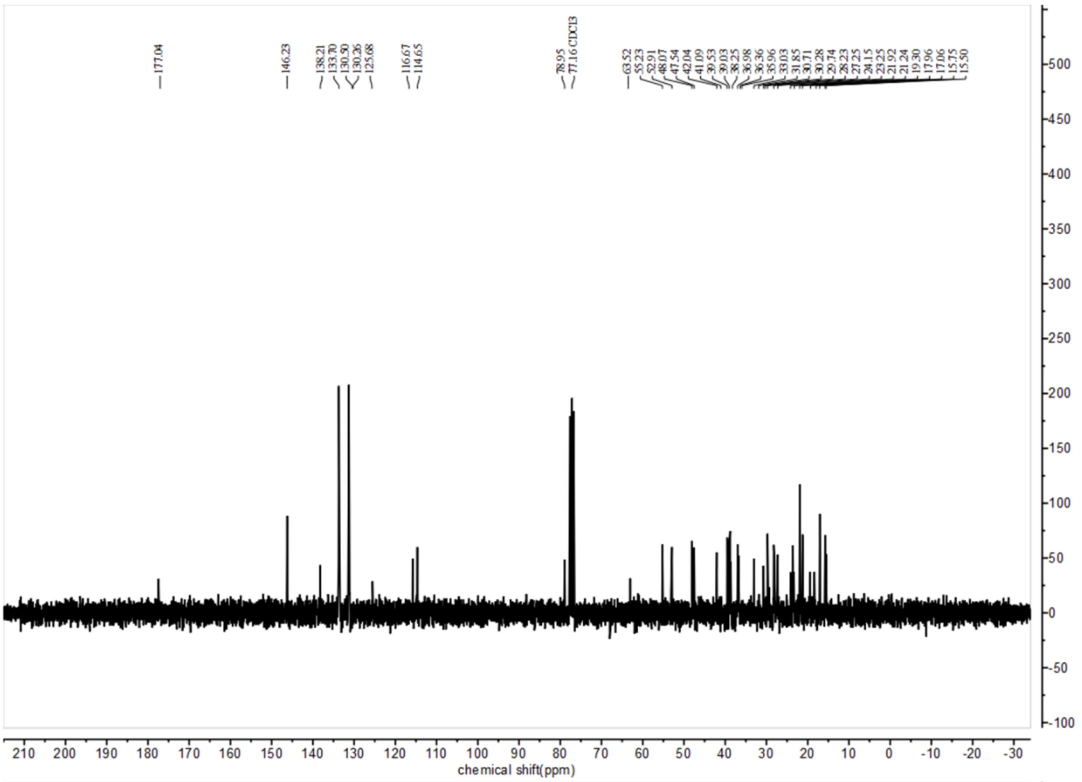

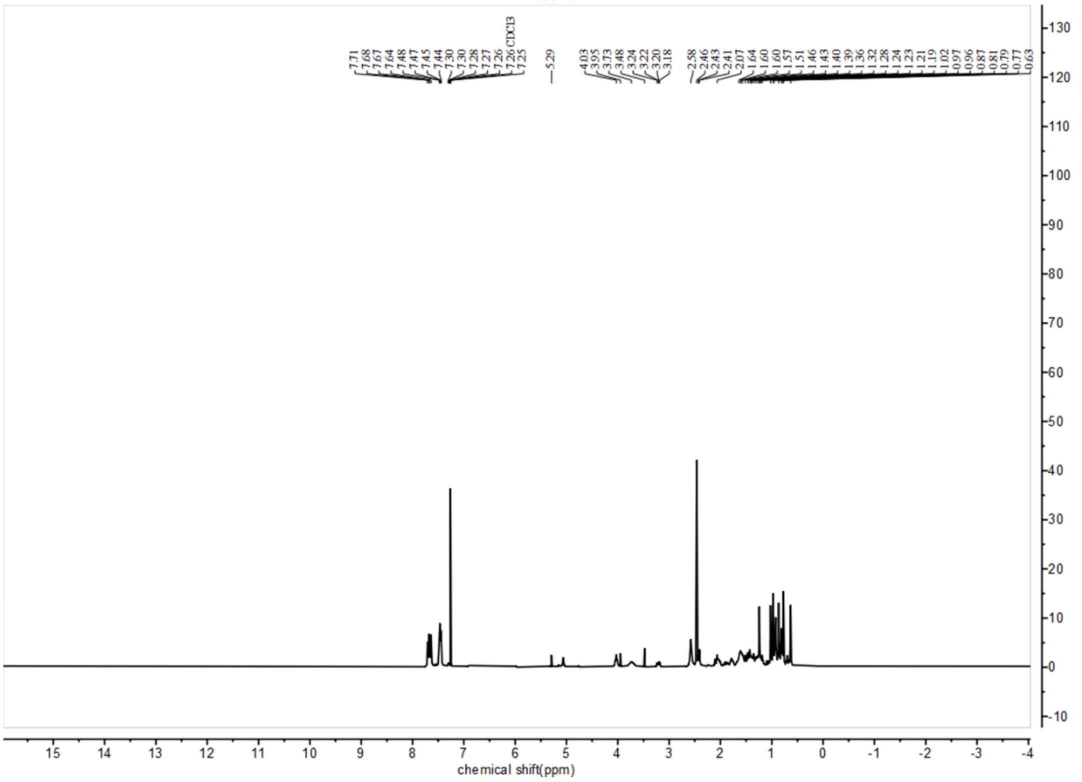

Figures S16. ^{13}C *and* ^{1}H *NMR of compound* ***16***.

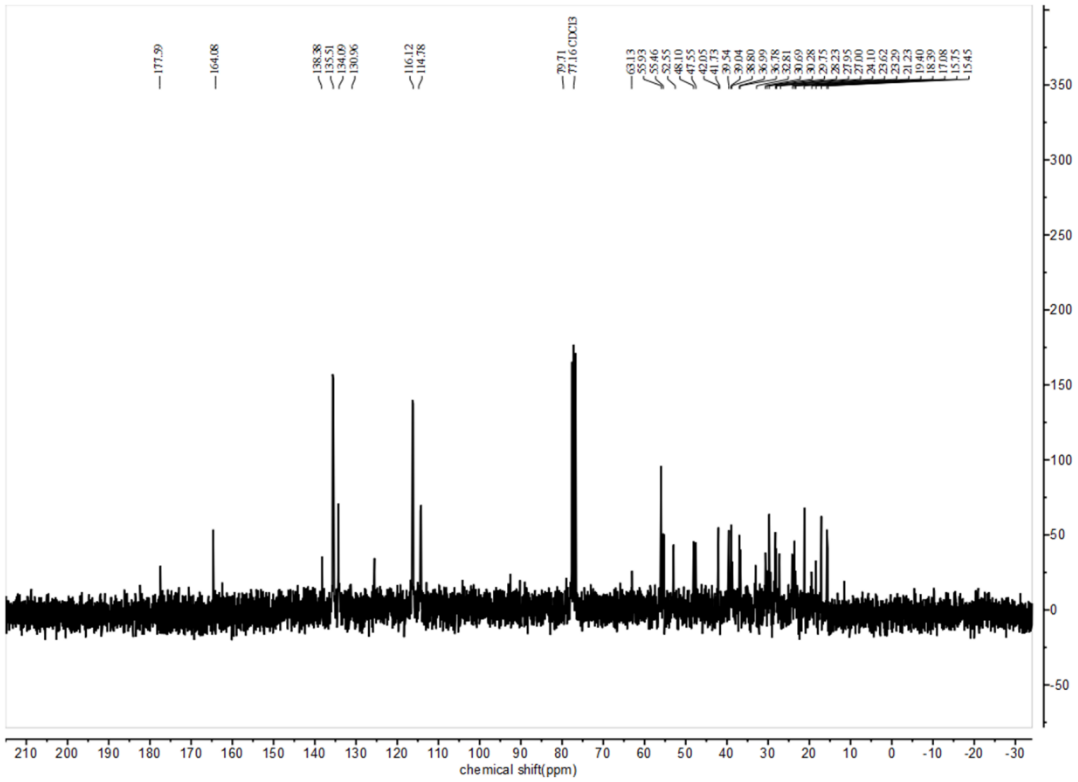

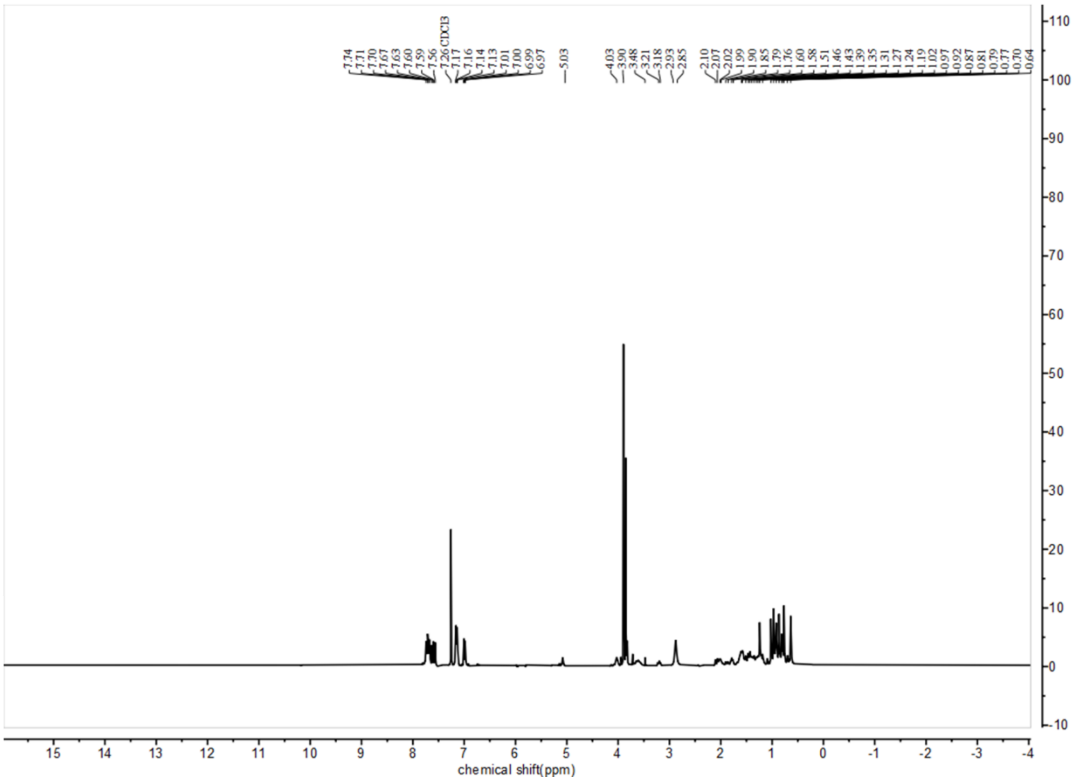

Figures S17. *^{13}C and ^{1}H NMR of compound **17**.*

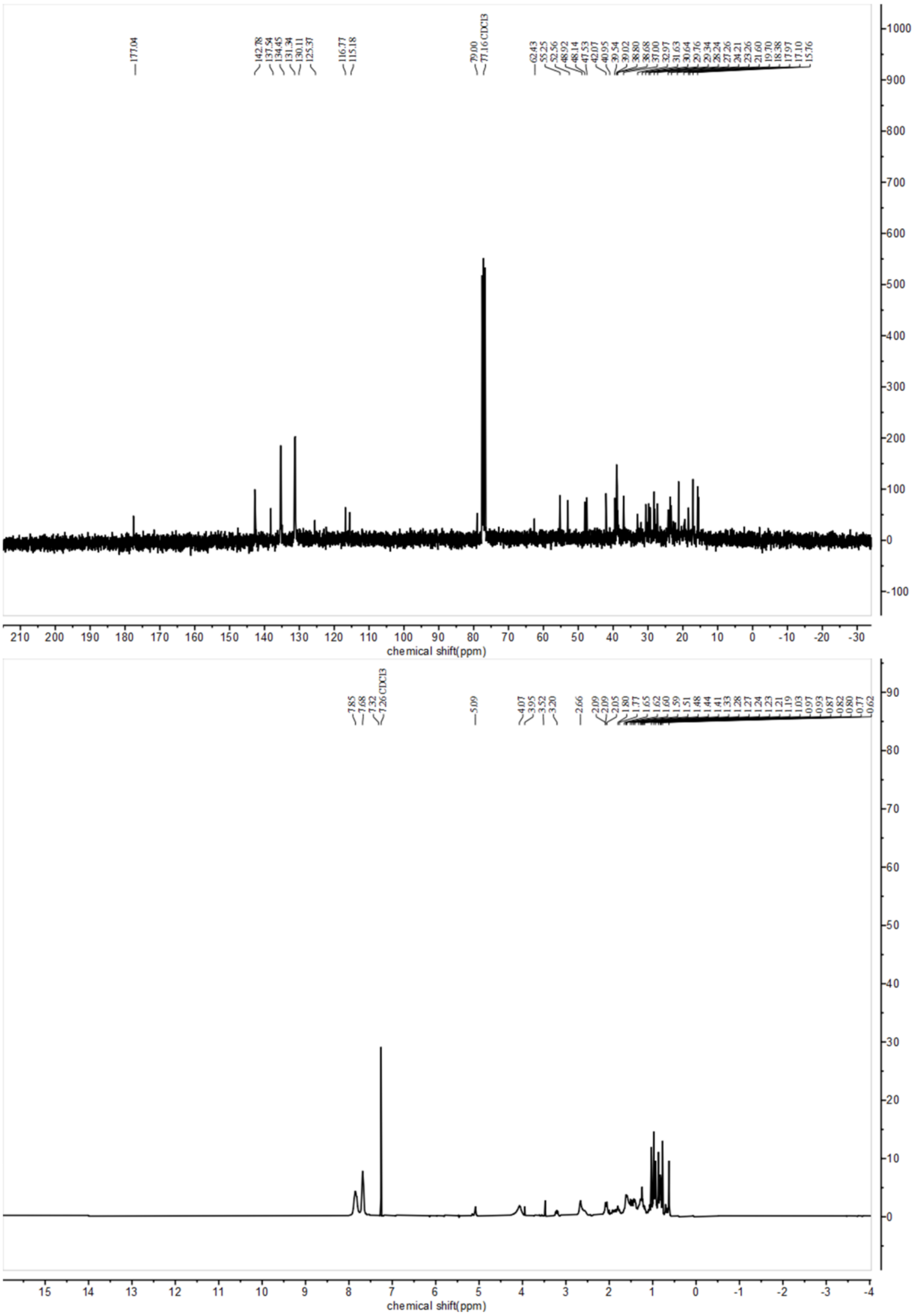

Figures S18. ^{13}C *and* ^{1}H *NMR of compound* ***18***.

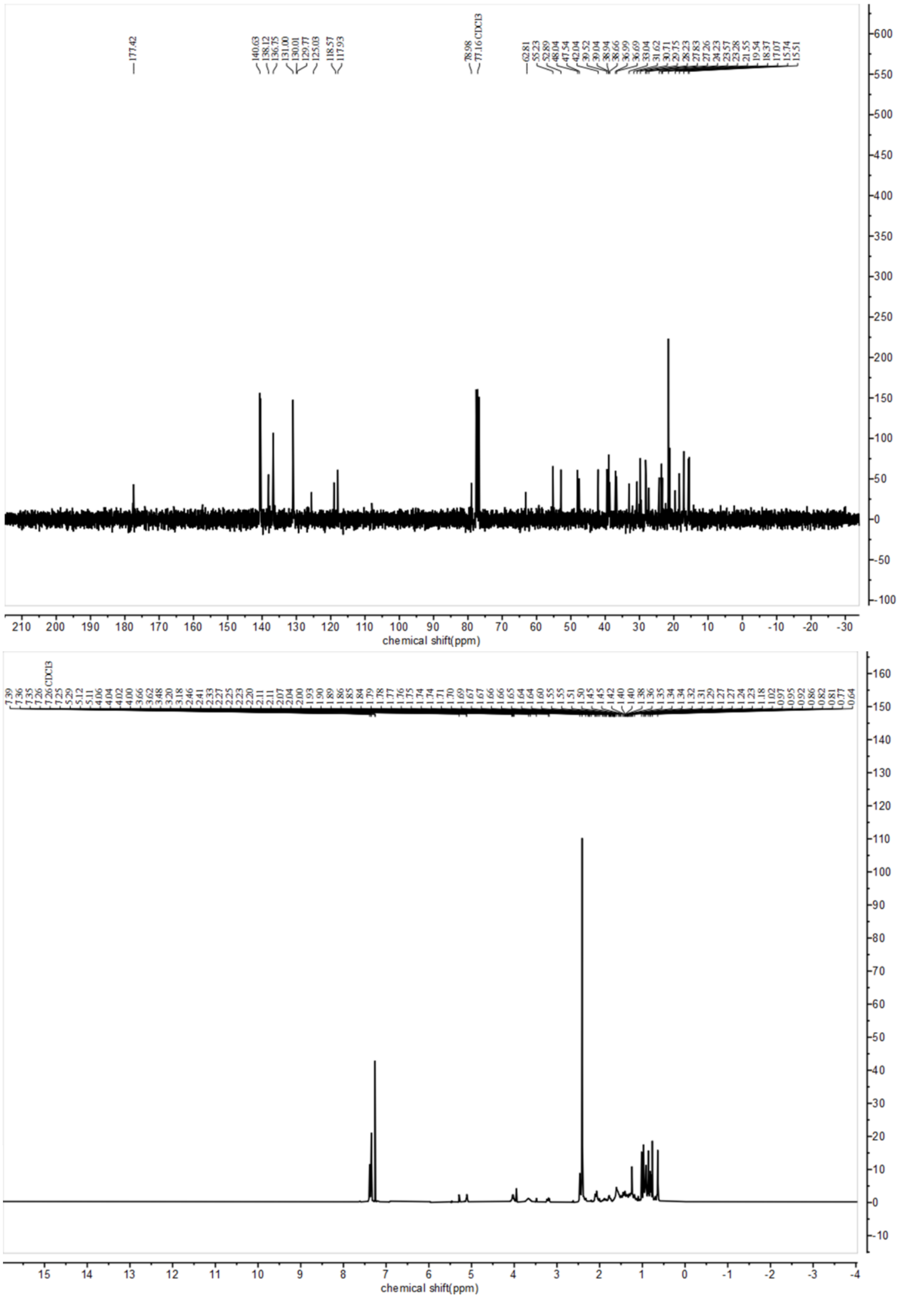

Figures S19. ^{13}C *and* ^{1}H *NMR of compound* ***19***.

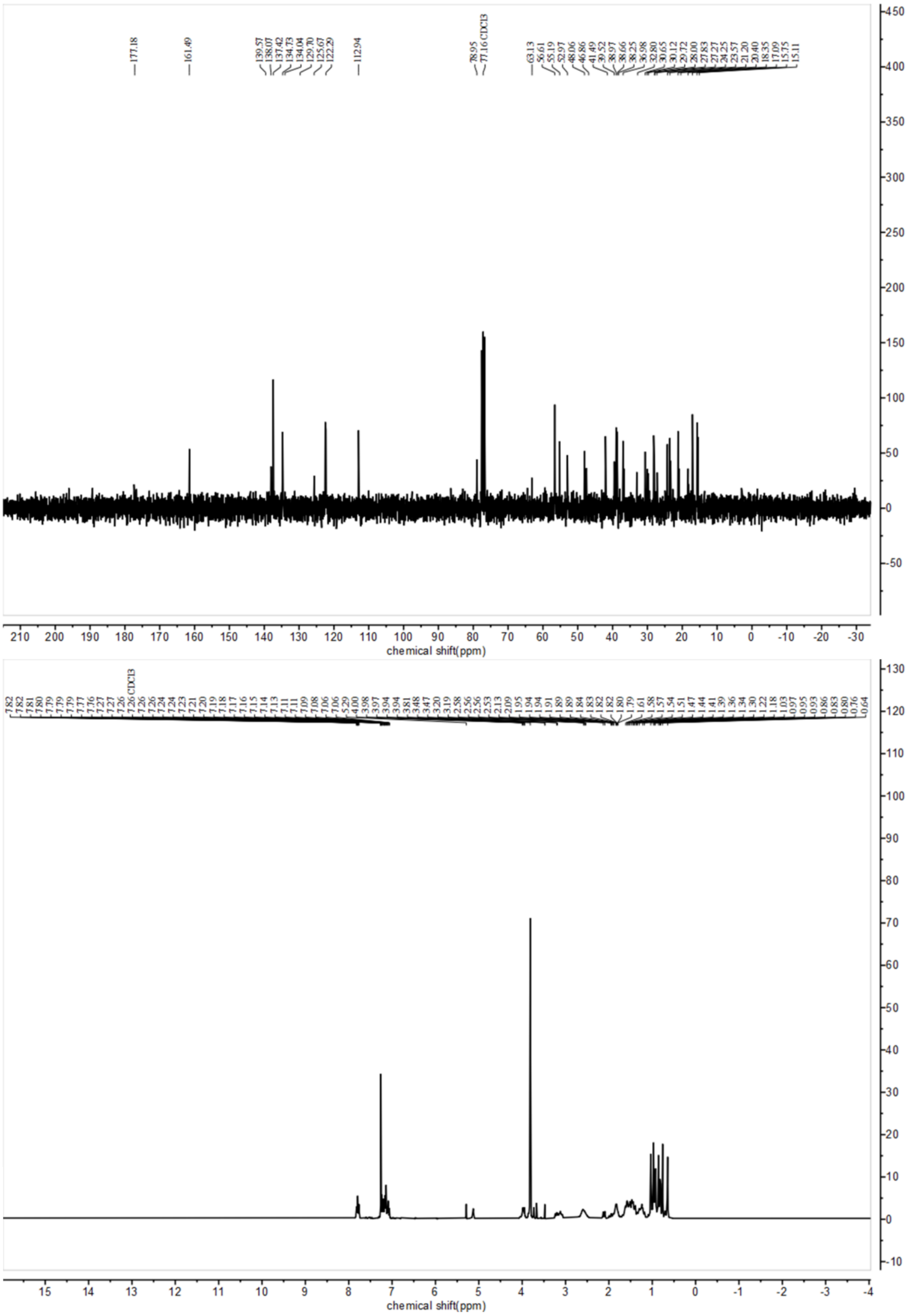

Figures S20. ^{13}C *and* ^{1}H *NMR of compound* ***20***.

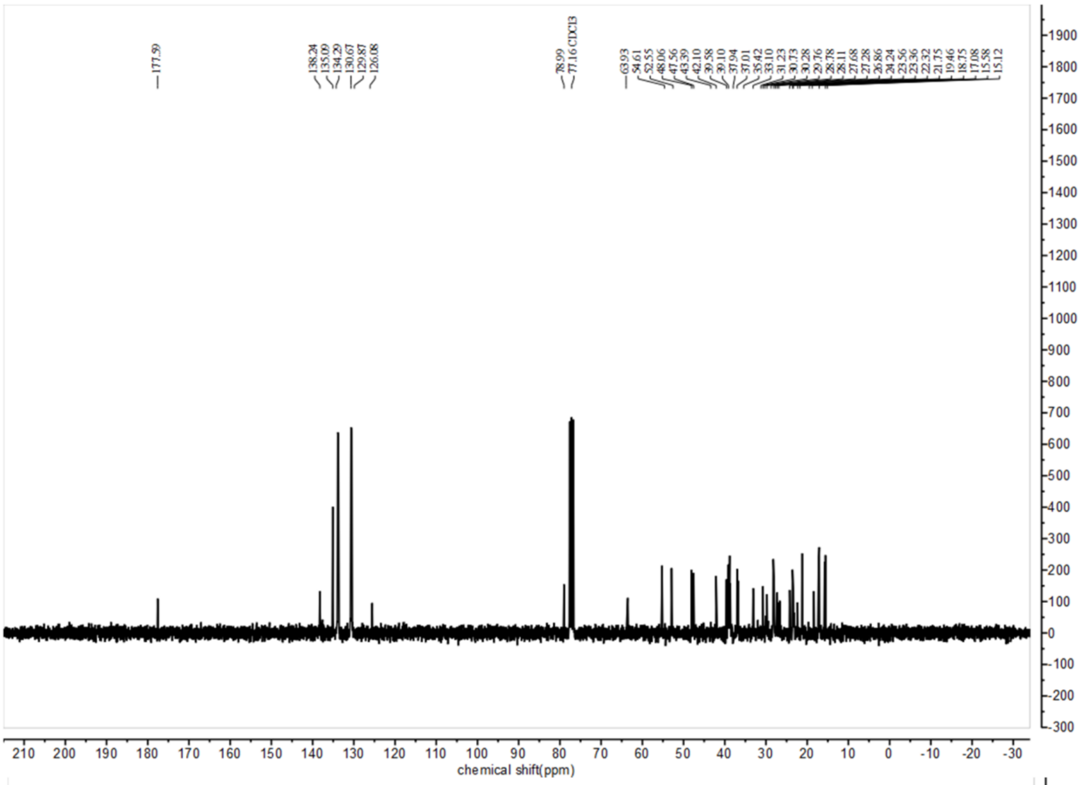

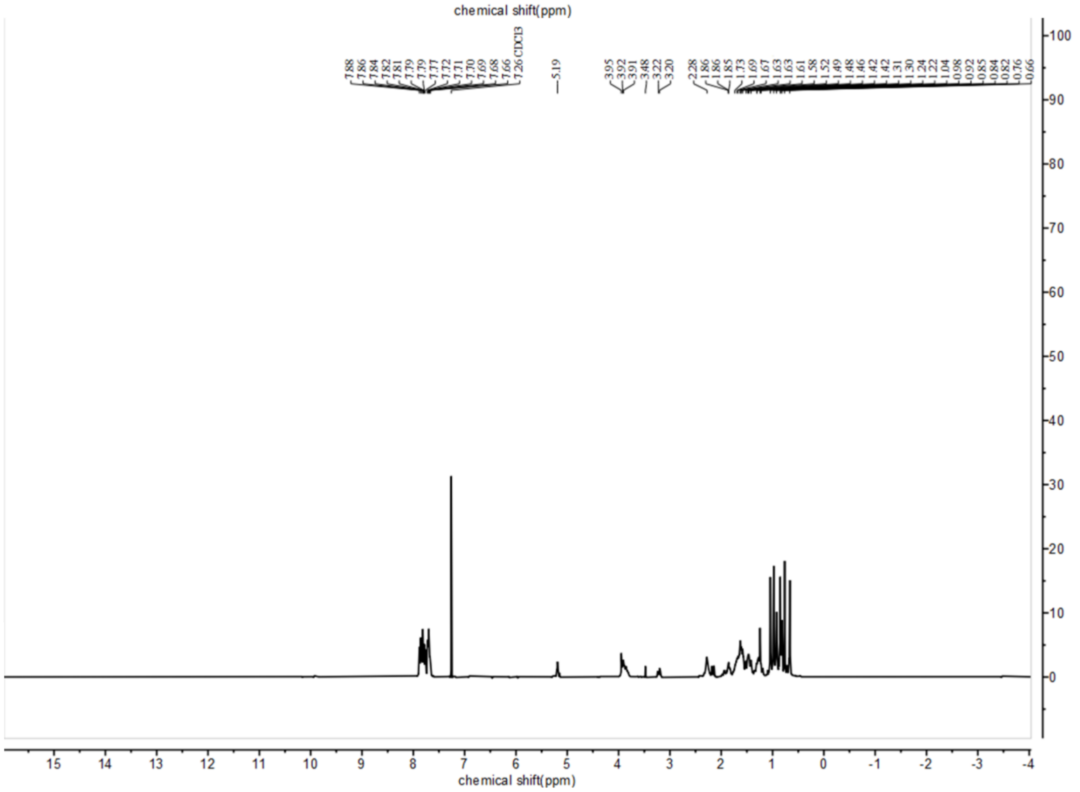

Figures S21. ^{13}C *and* ^{1}H *NMR of compound* ***21***.

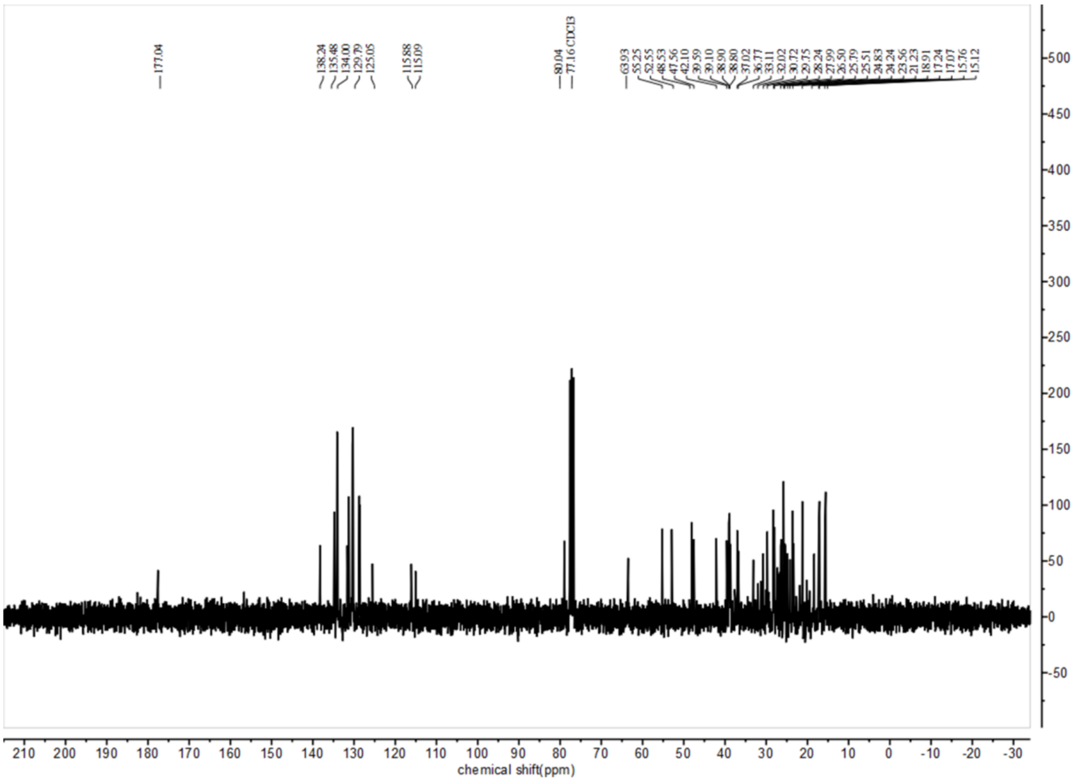

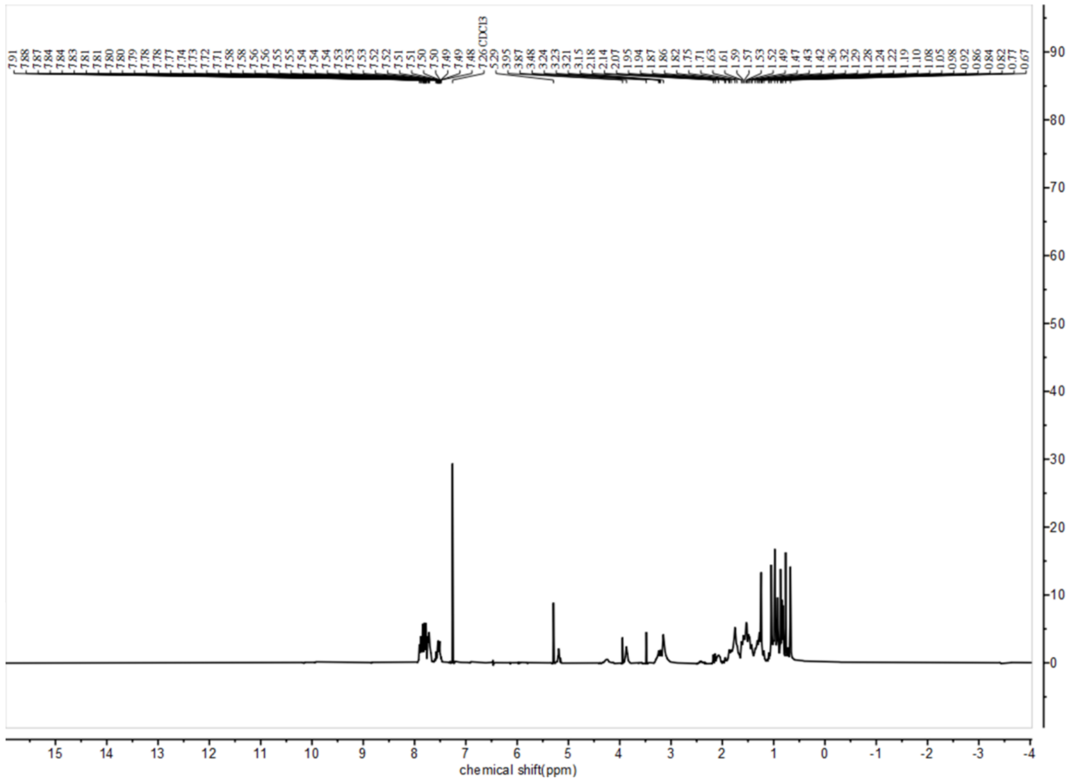

Figures S22. ^{13}C *and* ^{1}H *NMR of compound* ***22***.

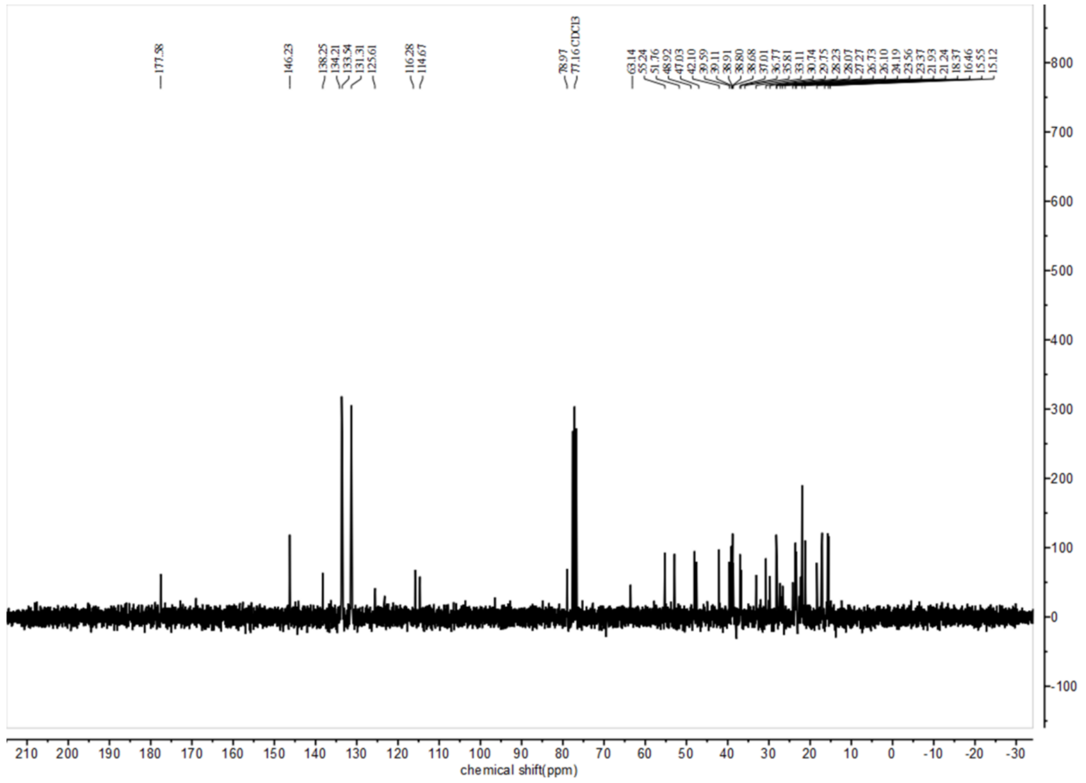

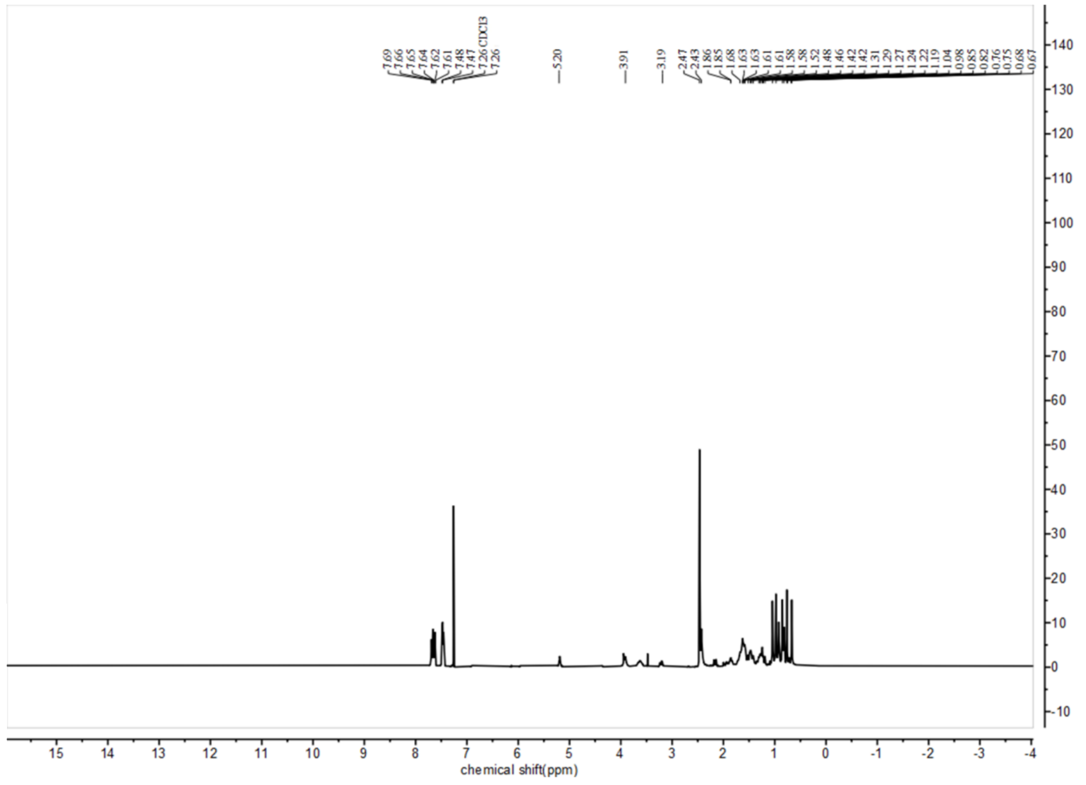

Figures S23. ^{13}*C and* ^{1}*H NMR of compound* ***23***.

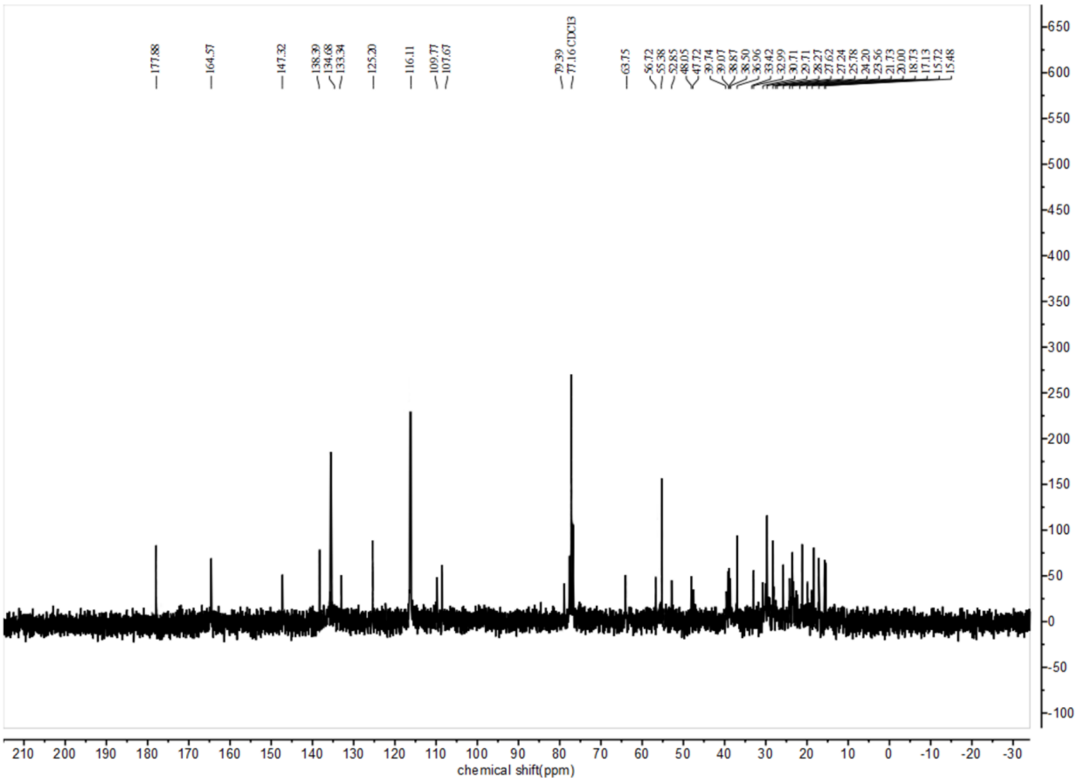

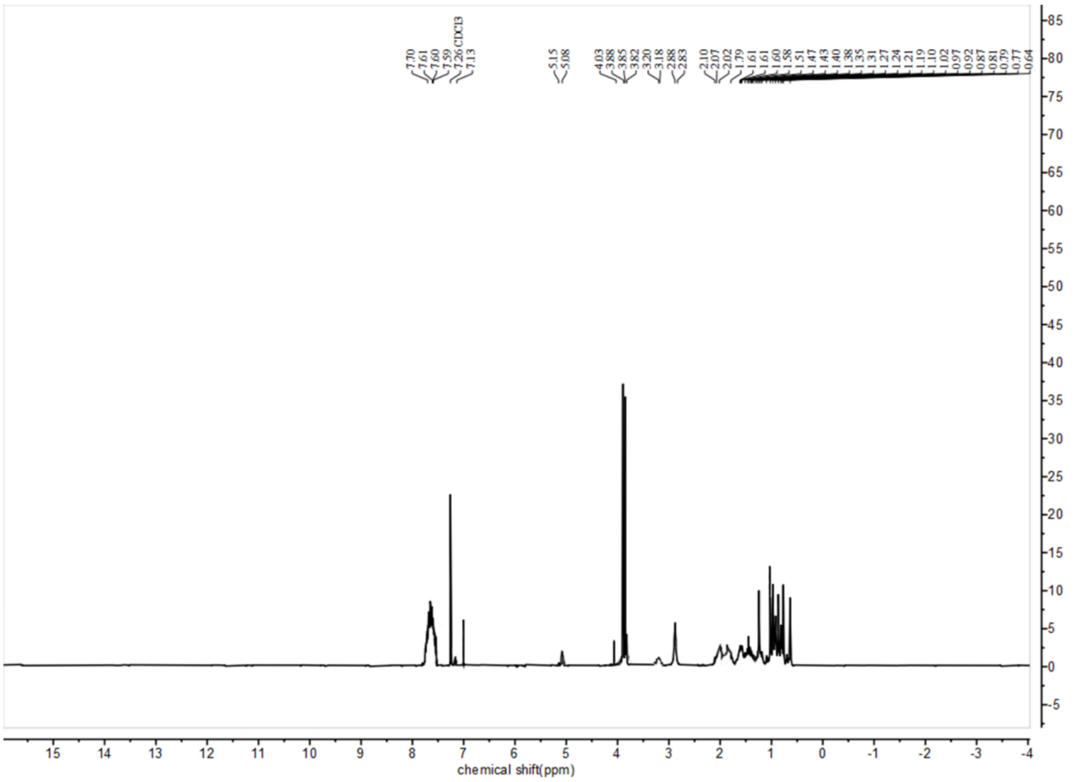

Figures S24. *^{13}C and ^{1}H NMR of compound **24**.*

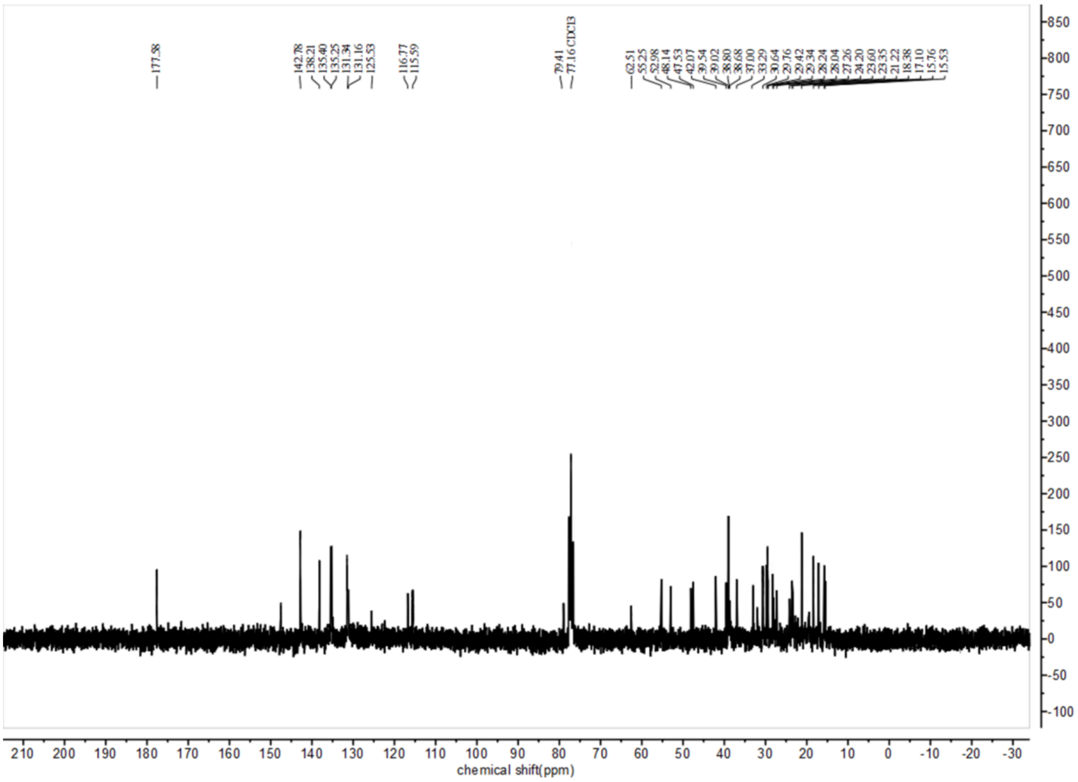

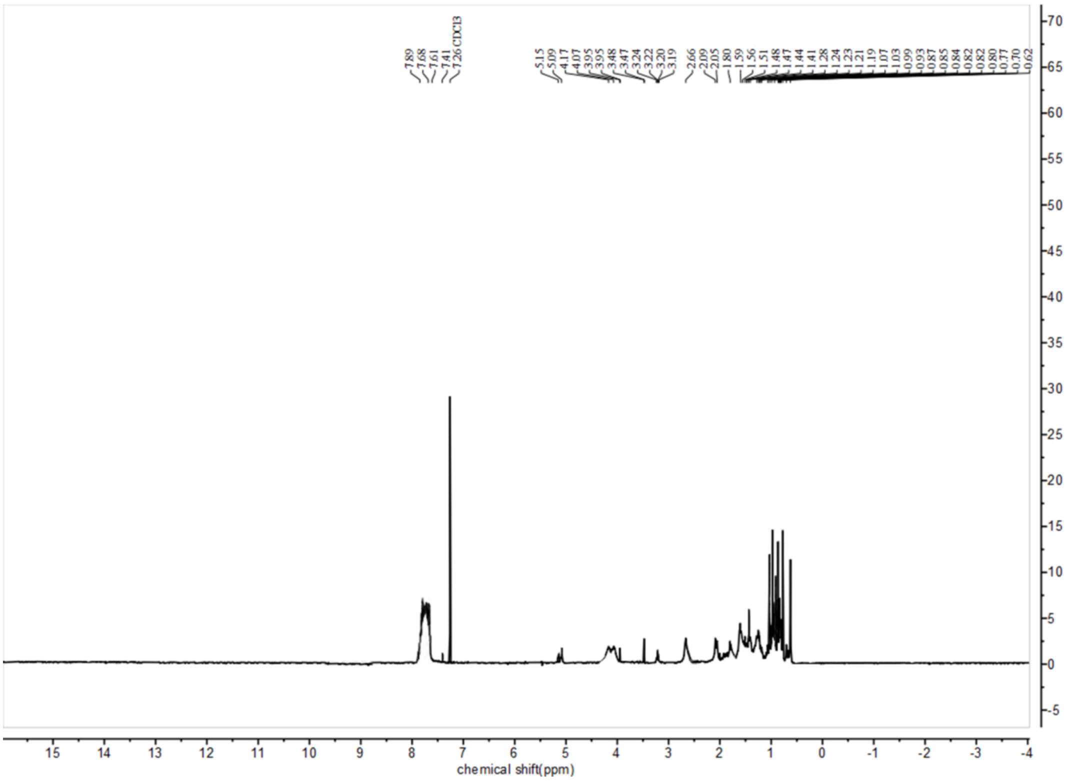

Figures S25. ^{13}*C and* ^{1}*H NMR of compound* ***25***.

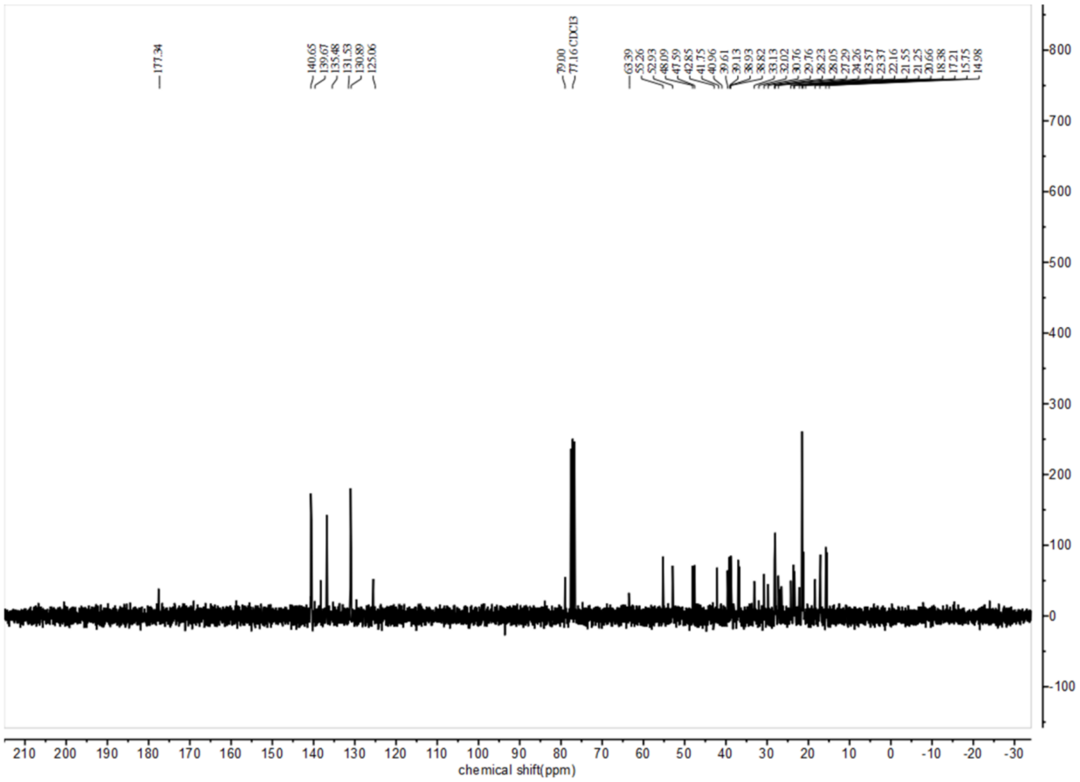

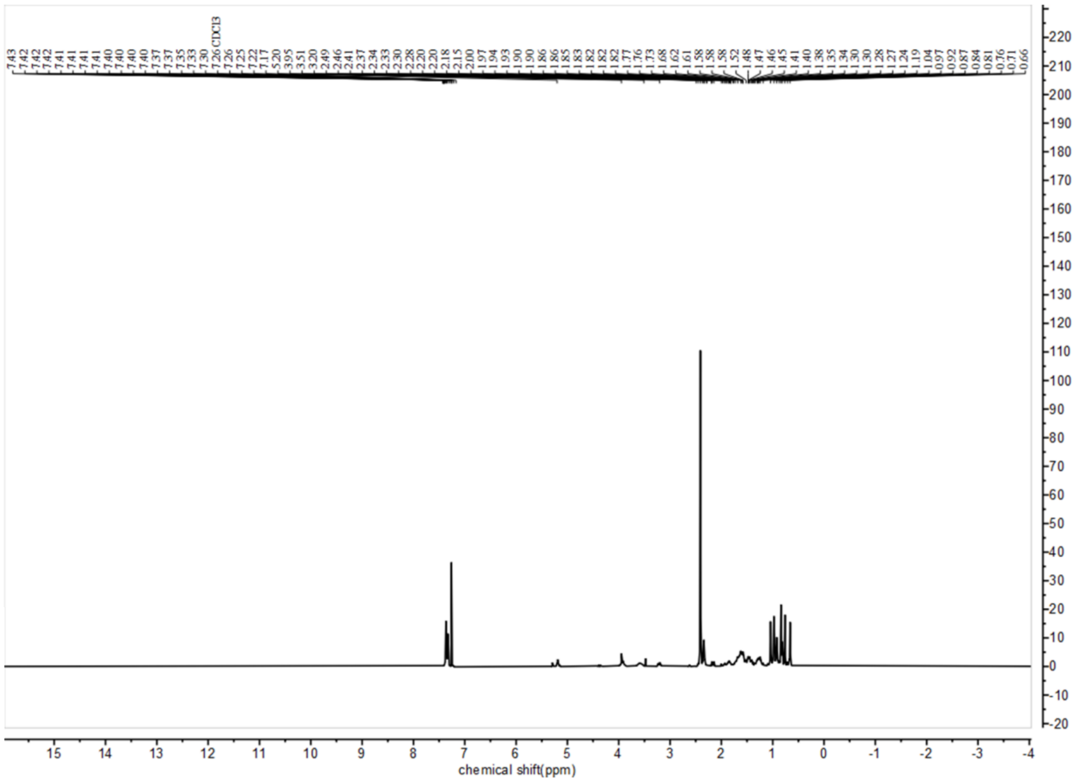

Figures S26. ^{13}C *and* ^{1}H *NMR of compound* ***26***.

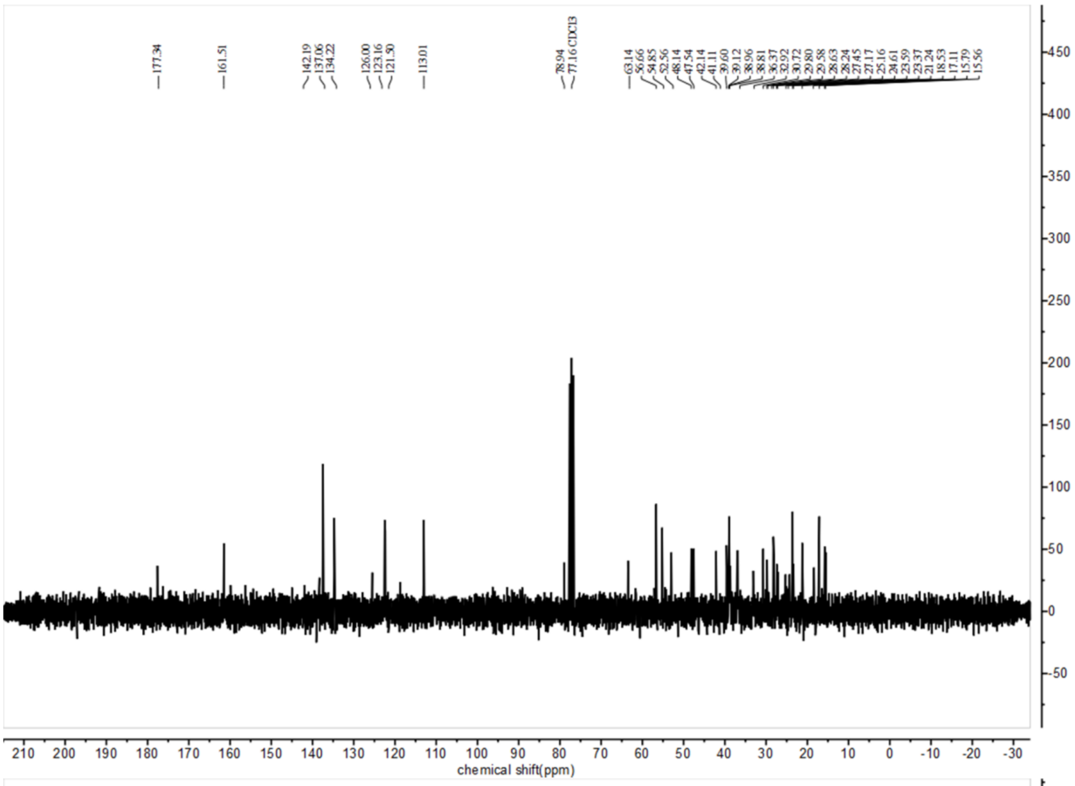

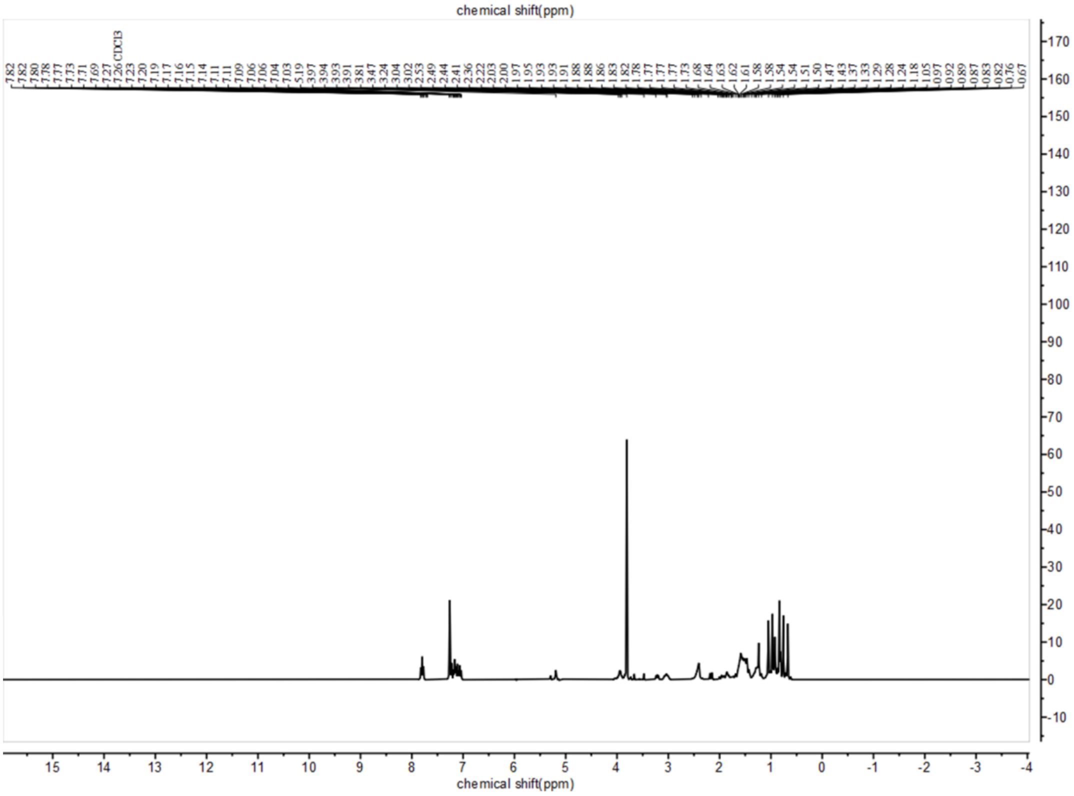

Figures S27. *^{13}C and ^{1}H NMR of compound **27**.*

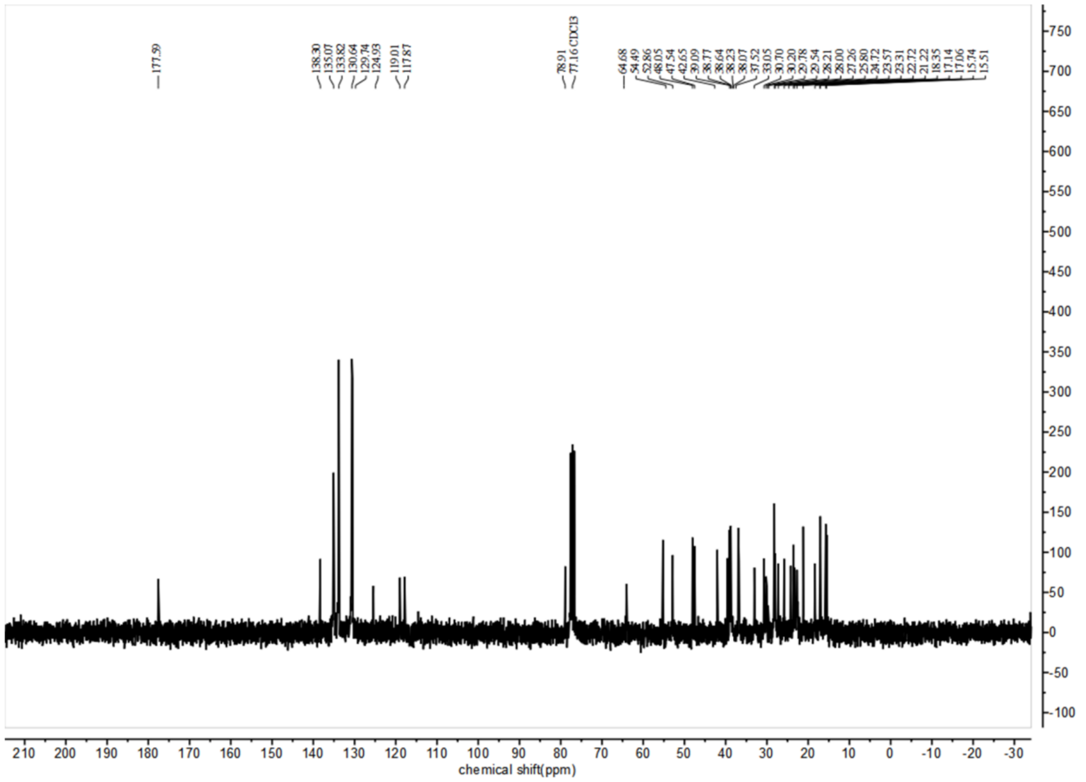

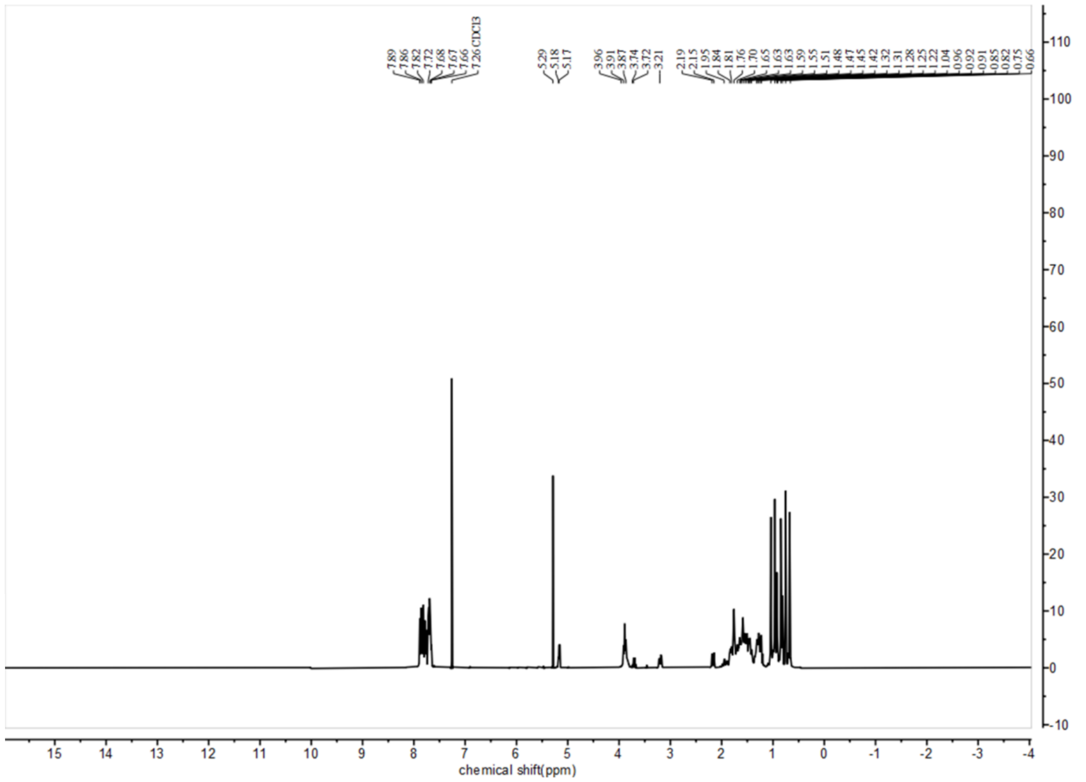

Figures S28. ^{13}C *and* ^{1}H *NMR of compound* ***28***.

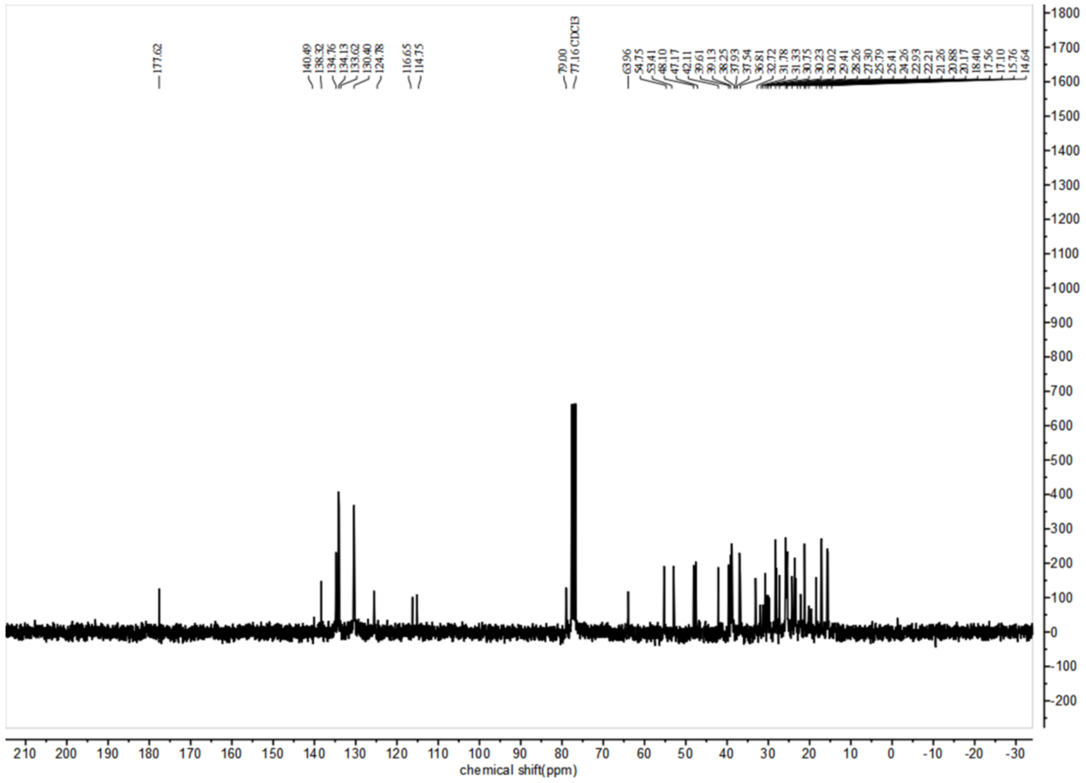

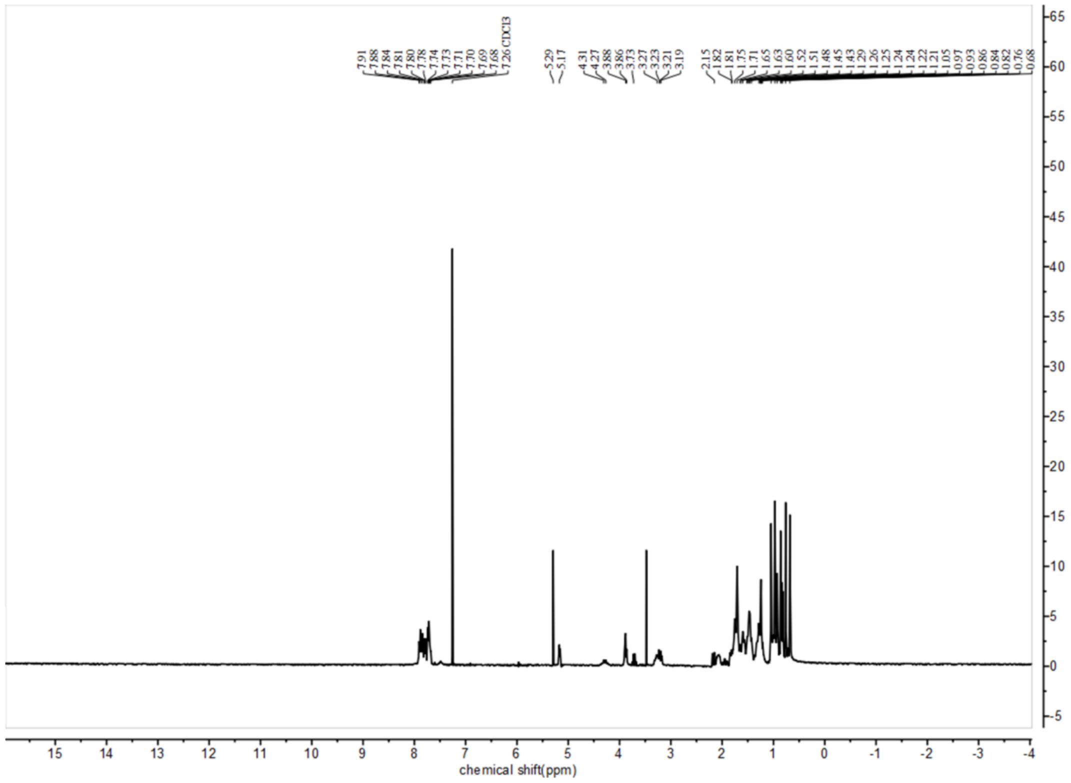

Figures S29. ^{13}*C and* ^{1}*H NMR of compound* ***29***.

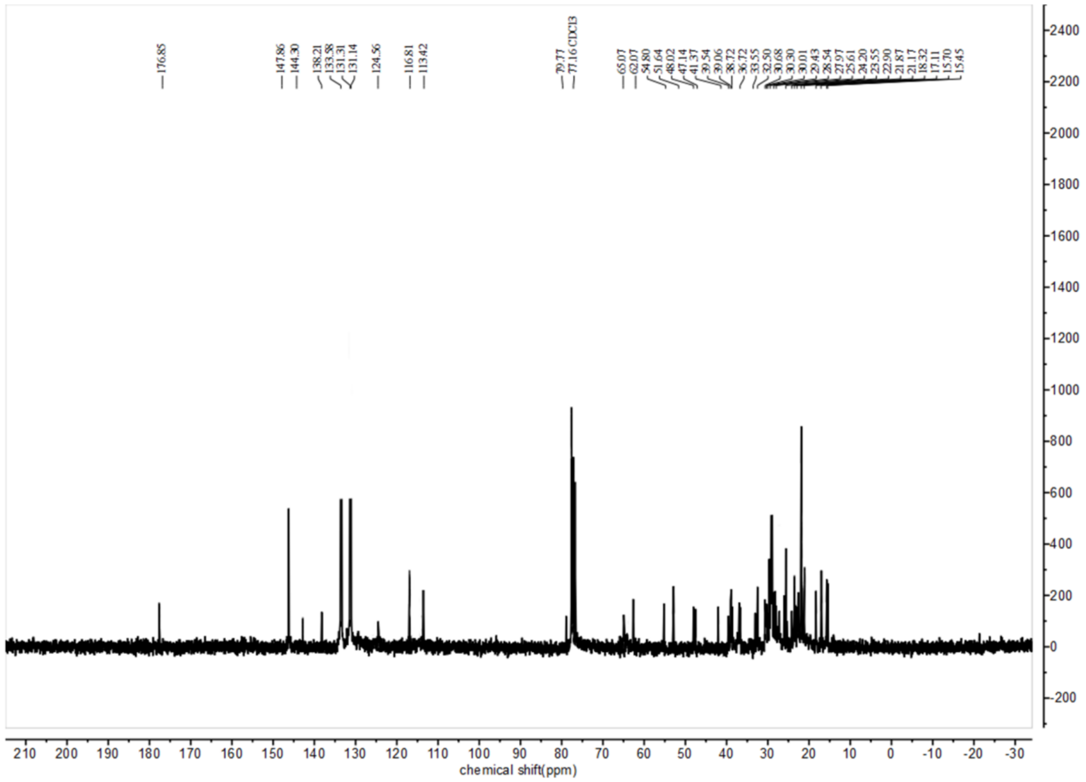

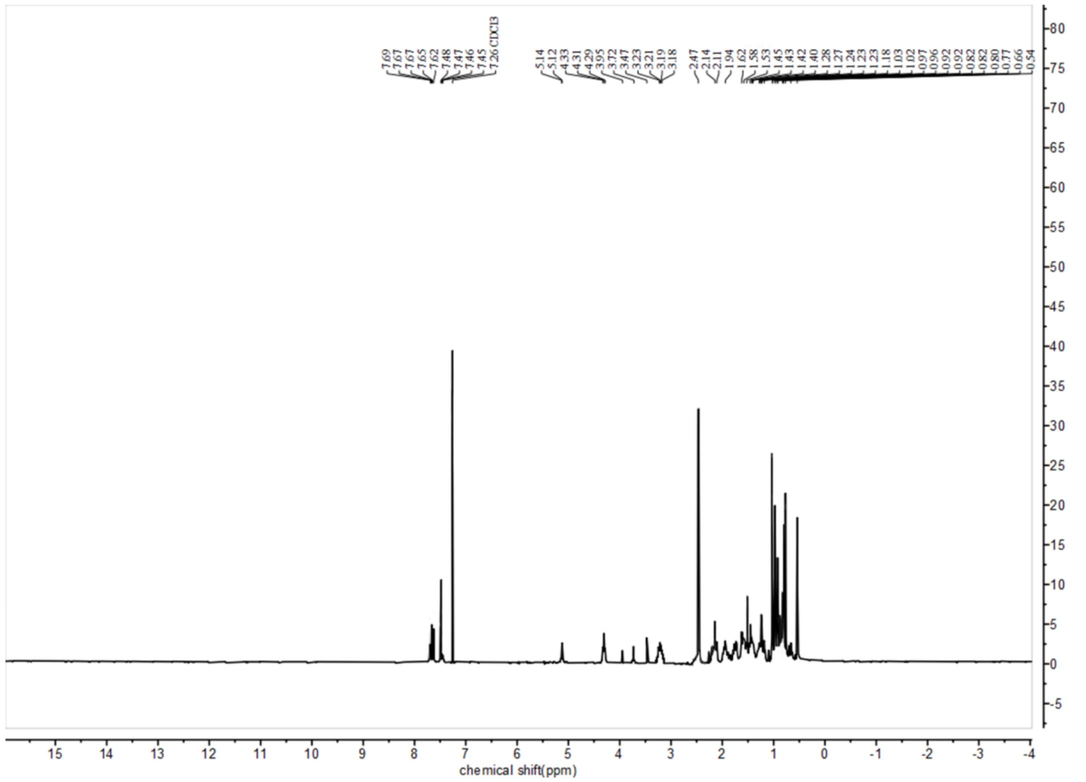

Figures S30. ^{13}C *and* ^{1}H *NMR of compound* ***30***.

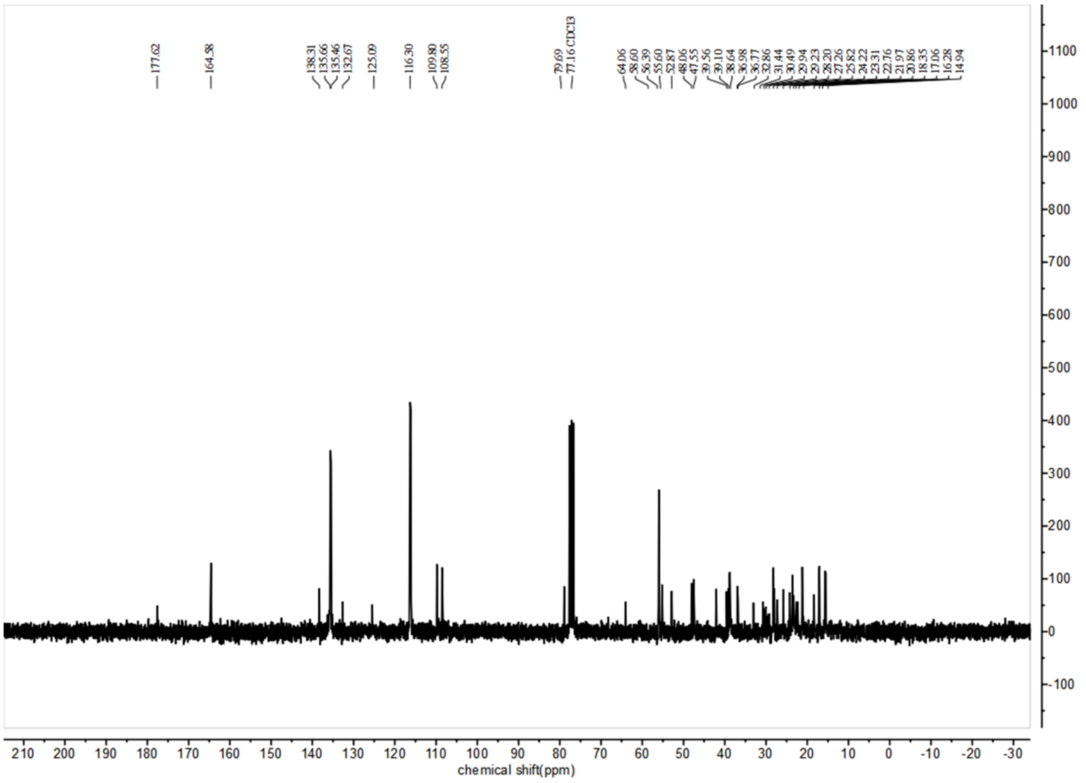

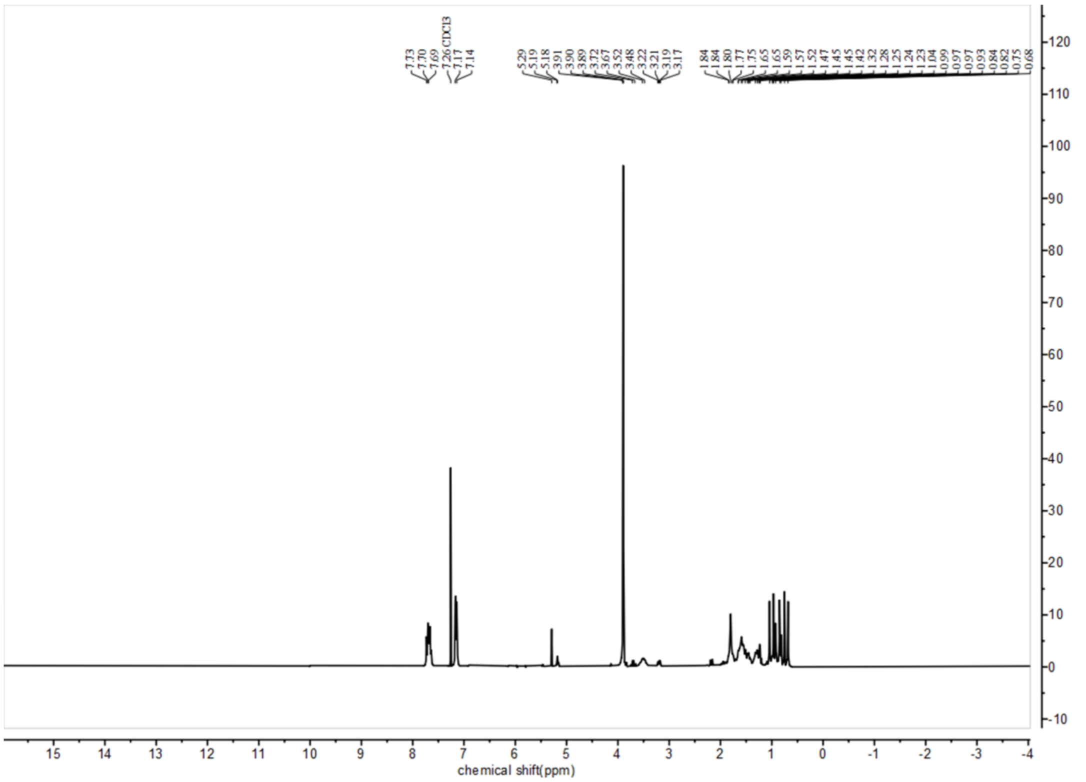

Figures S31. *^{13}C and ^{1}H NMR of compound **31***.

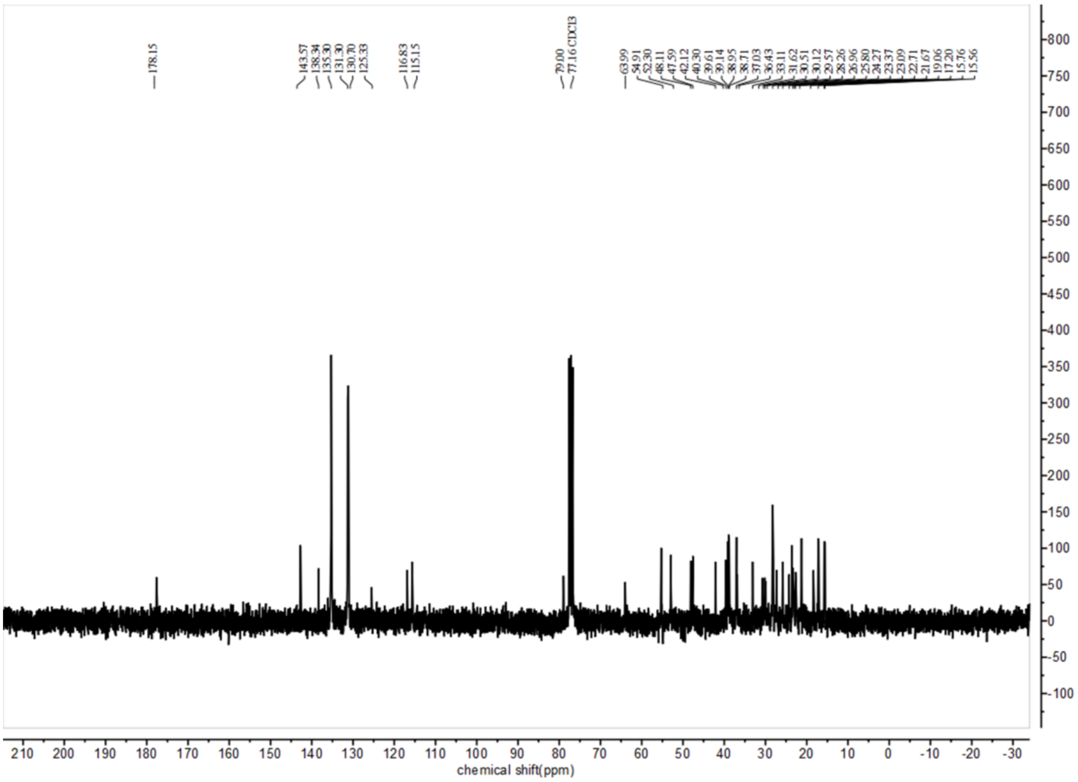

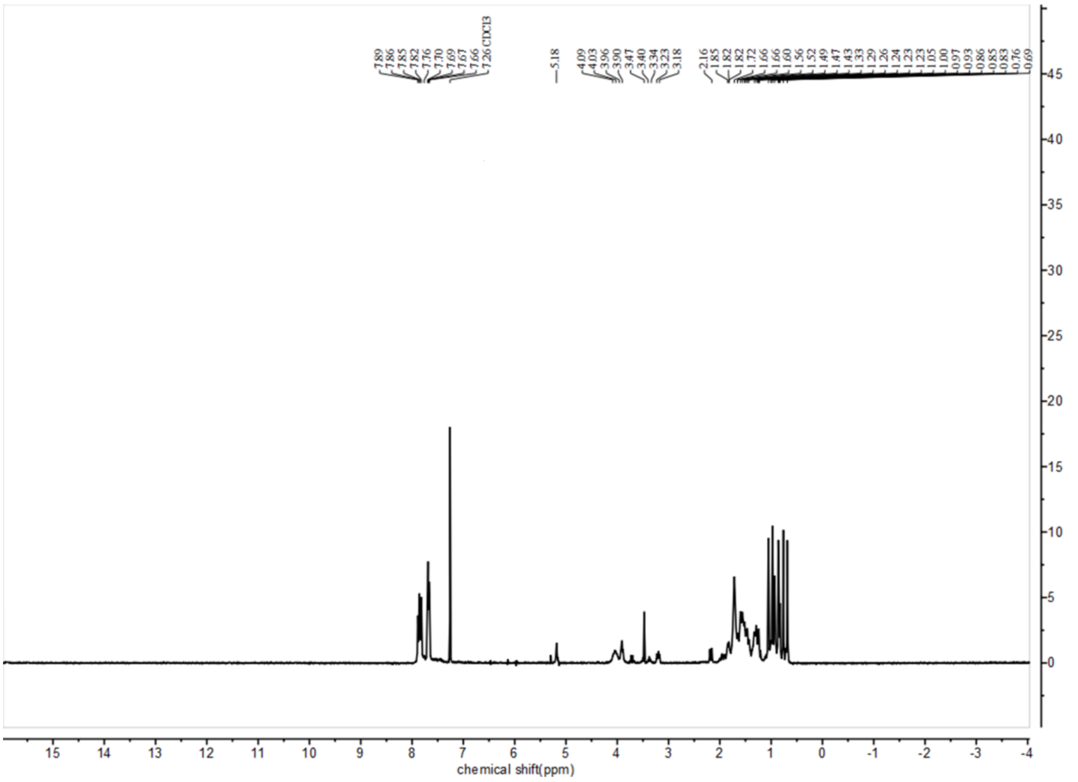

Figures S32. ^{13}C *and* ^{1}H *NMR of compound* ***32***.

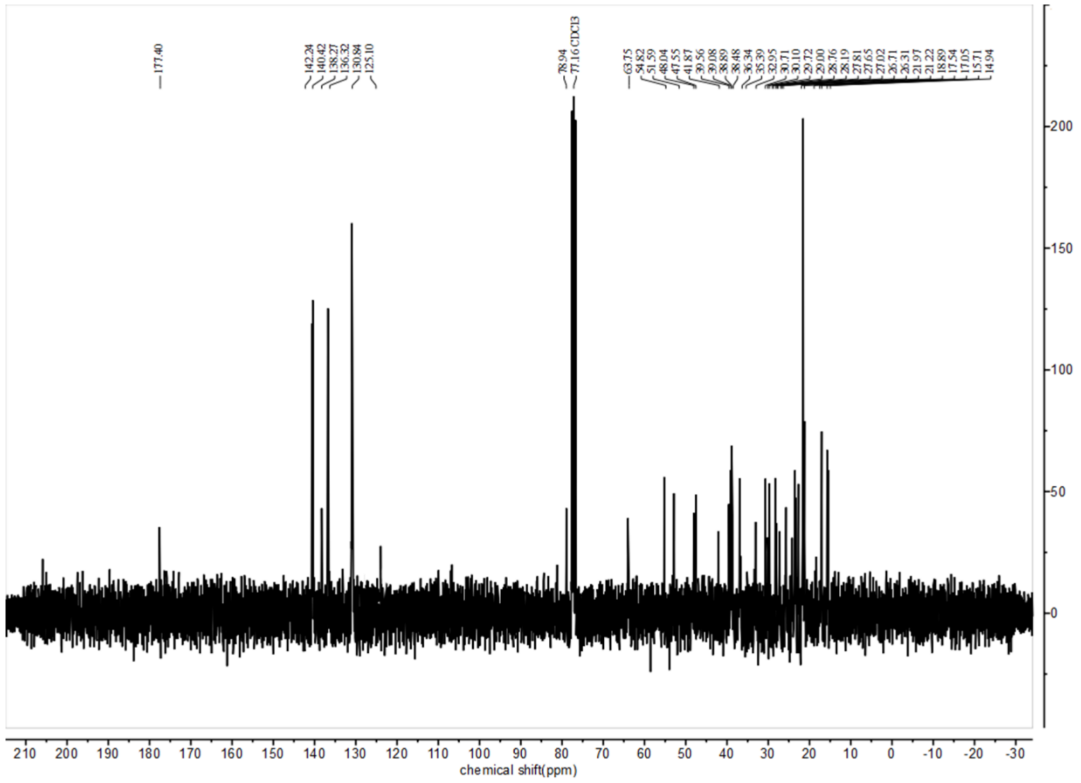

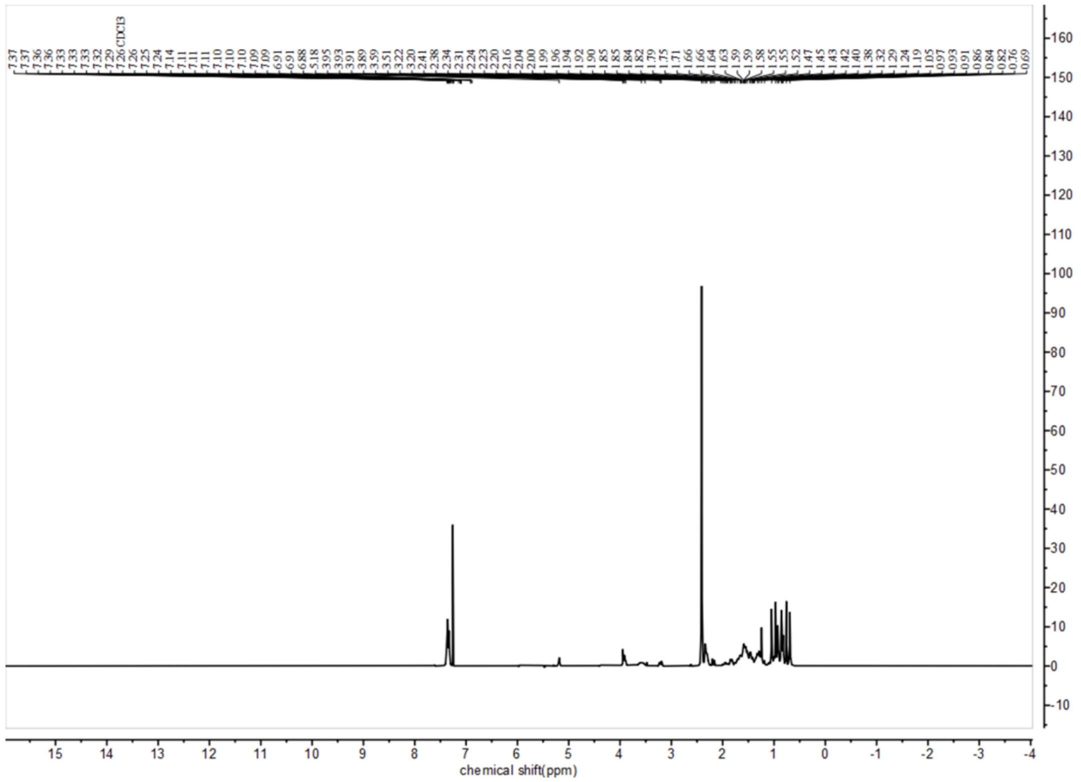

Figures S33. ${}^{13}C$ *and* ${}^{1}H$ *NMR of compound* ***33***.

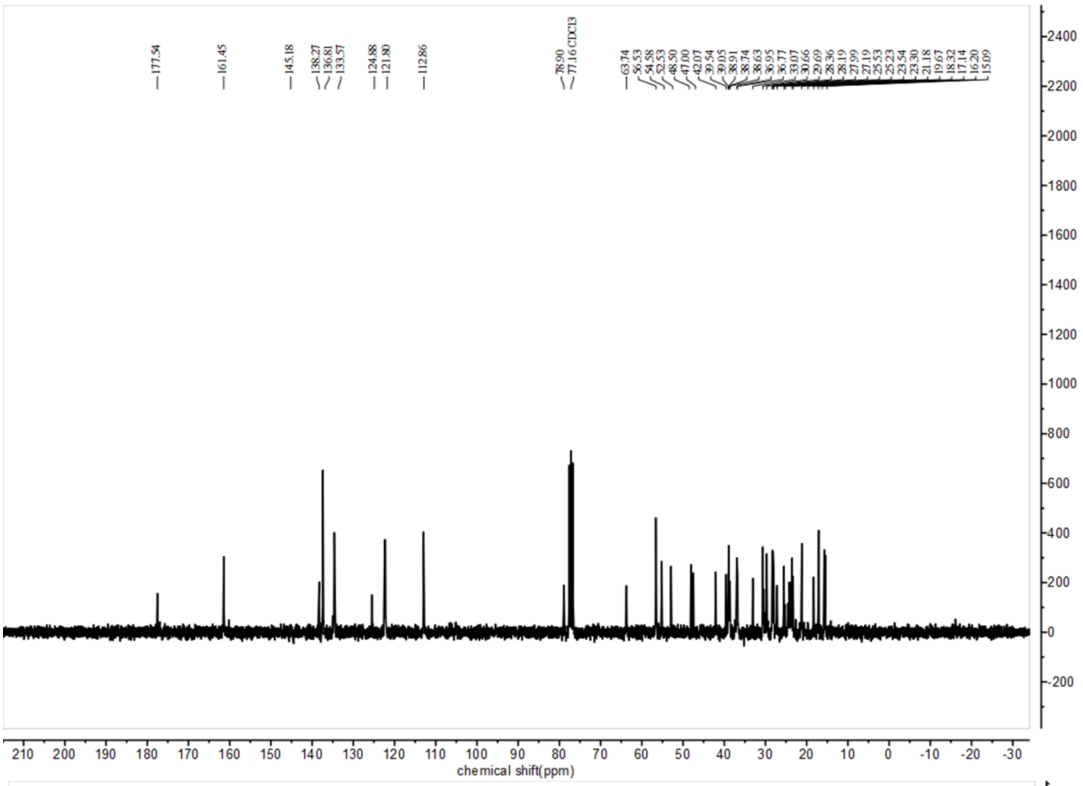

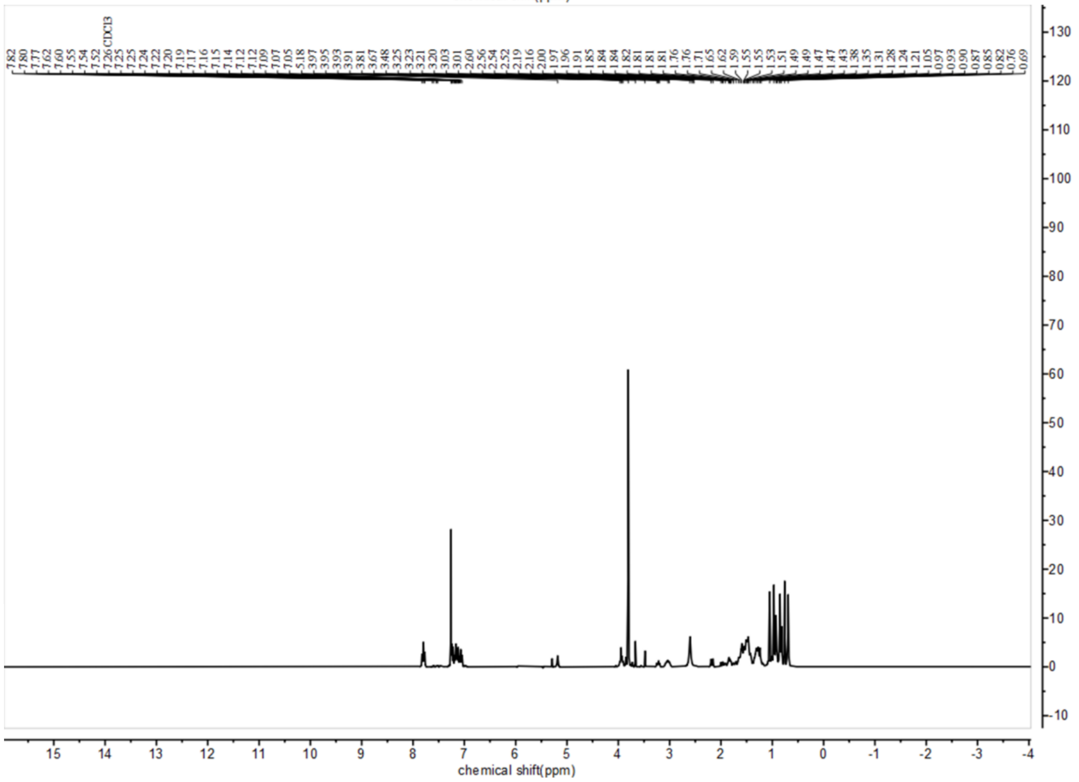

Figures S34. ^{13}C *and* ^{1}H *NMR of compound* ***34***.

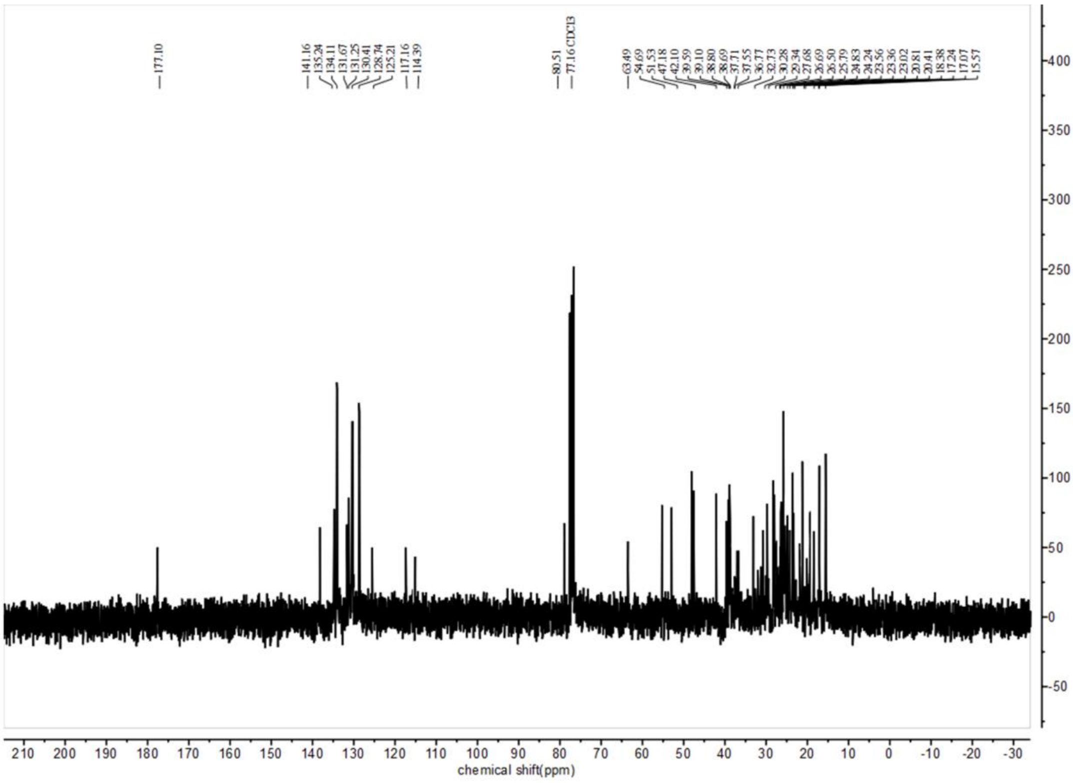

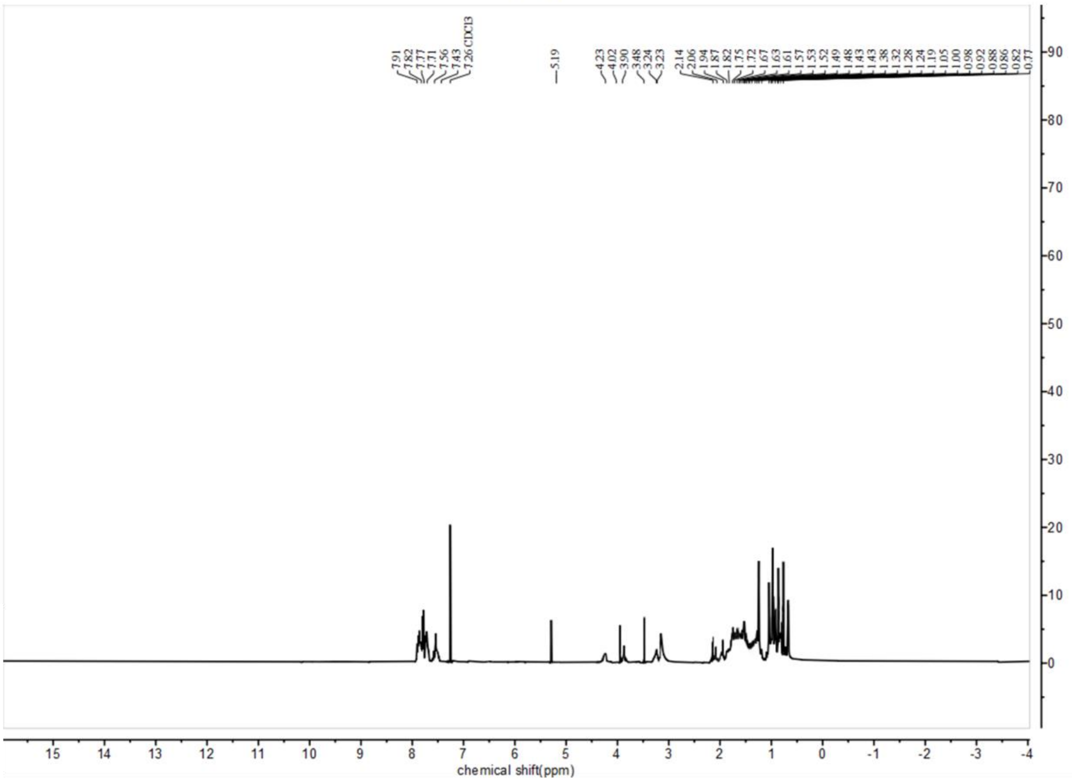

Figures S35. ^{13}C *and* ^{1}H *NMR of compound* ***35****.*

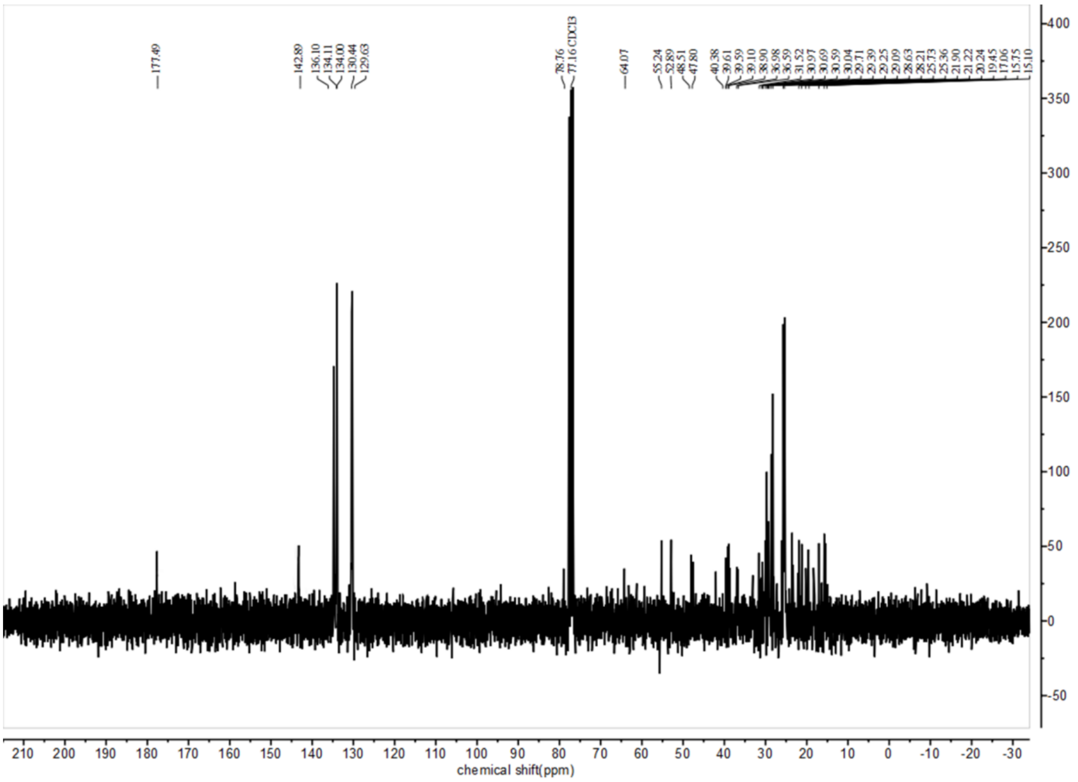

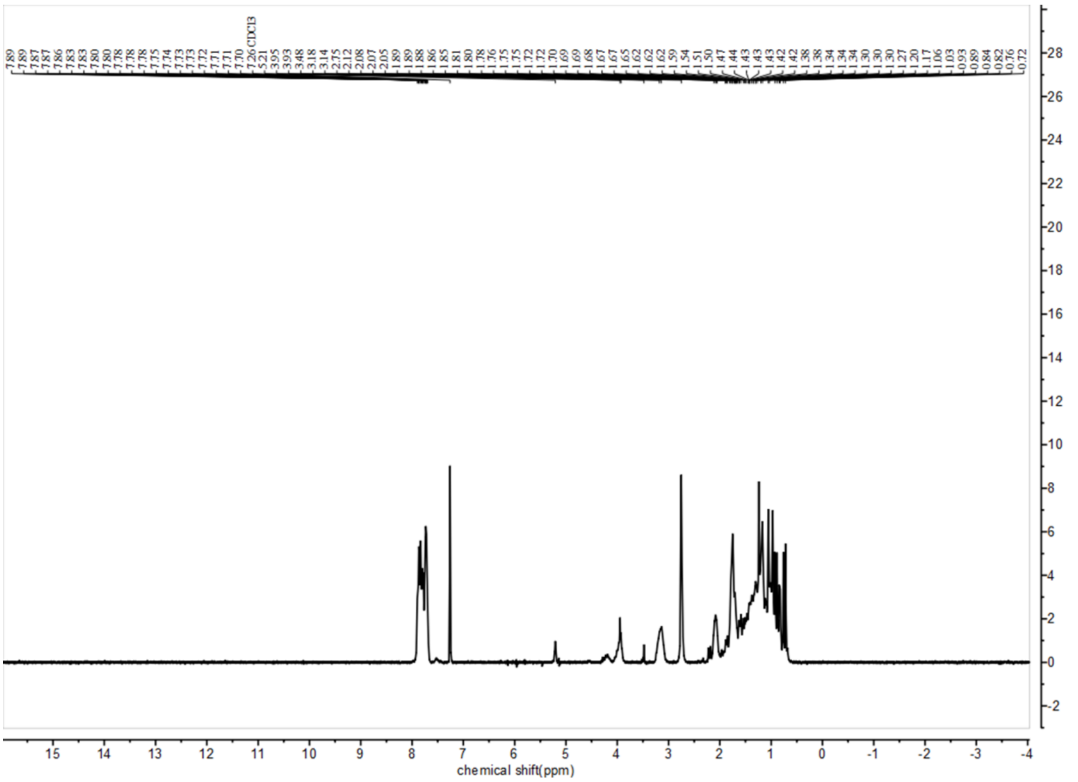

Figures S36. *^{13}C and ^{1}H NMR of compound **36***.

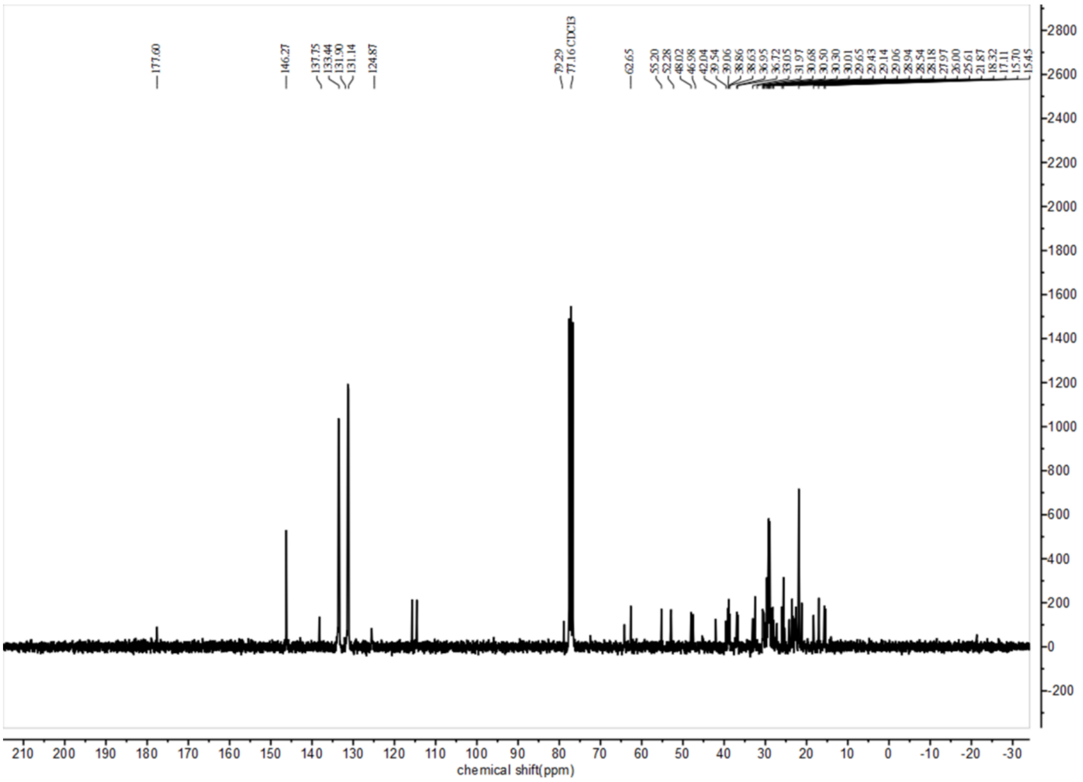

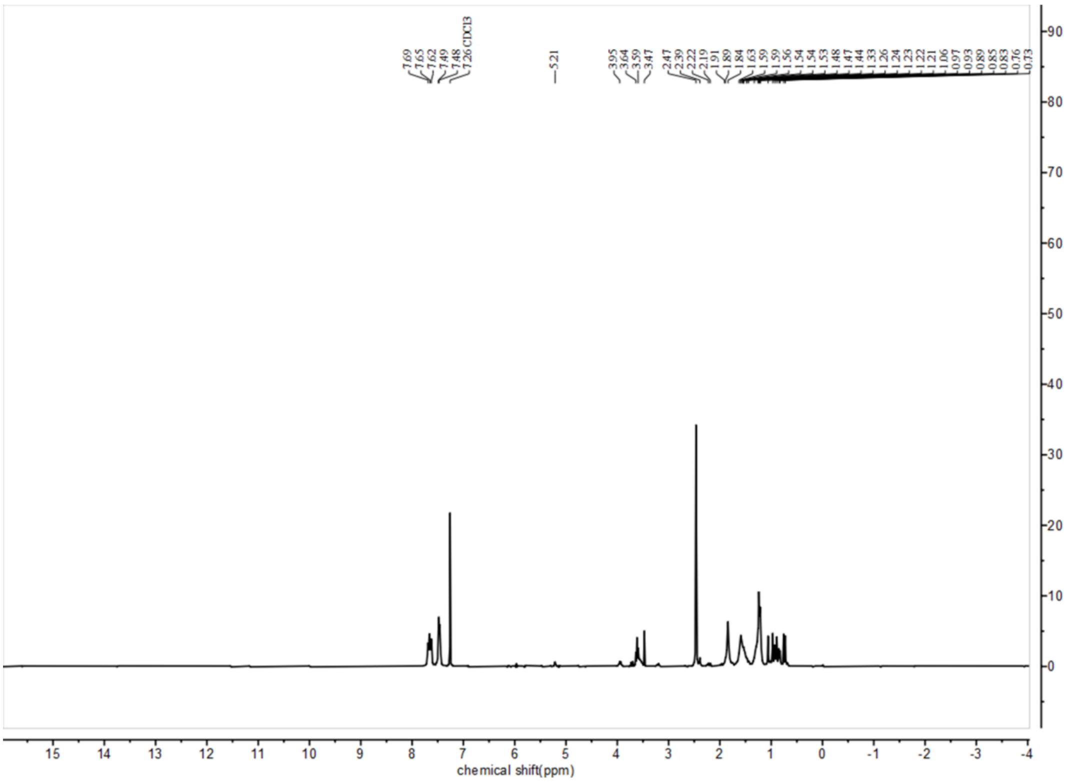

Figures S37. ^{13}C *and* ^{1}H *NMR of compound **37**.*

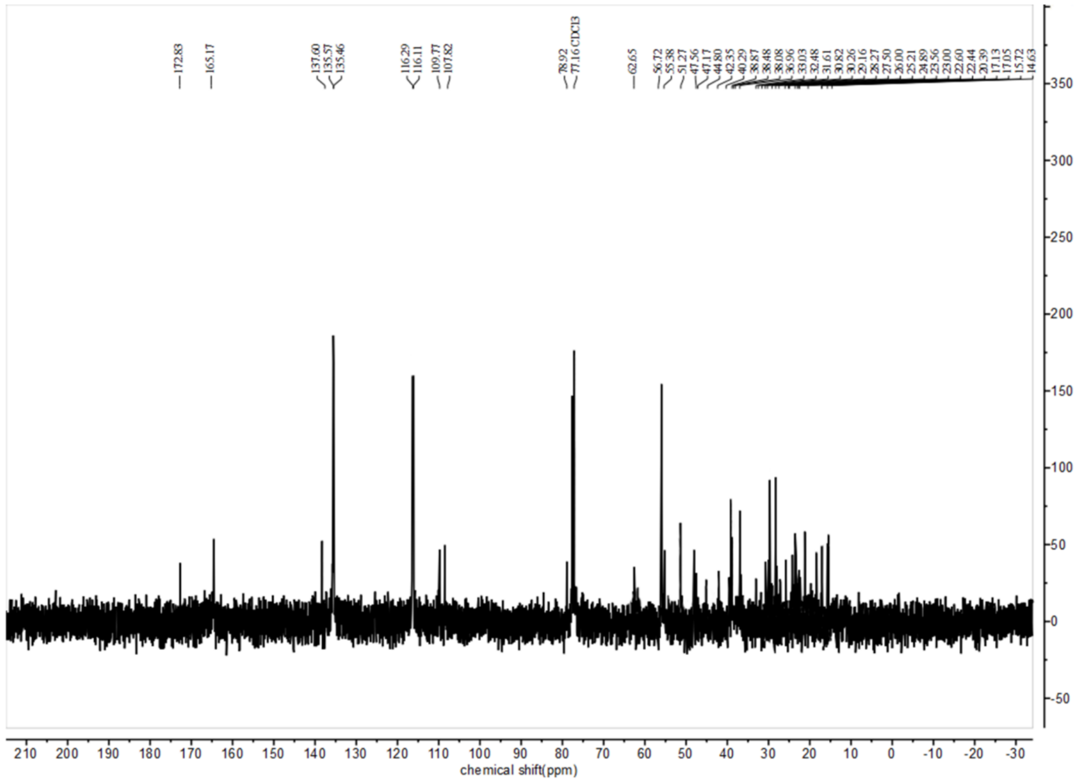

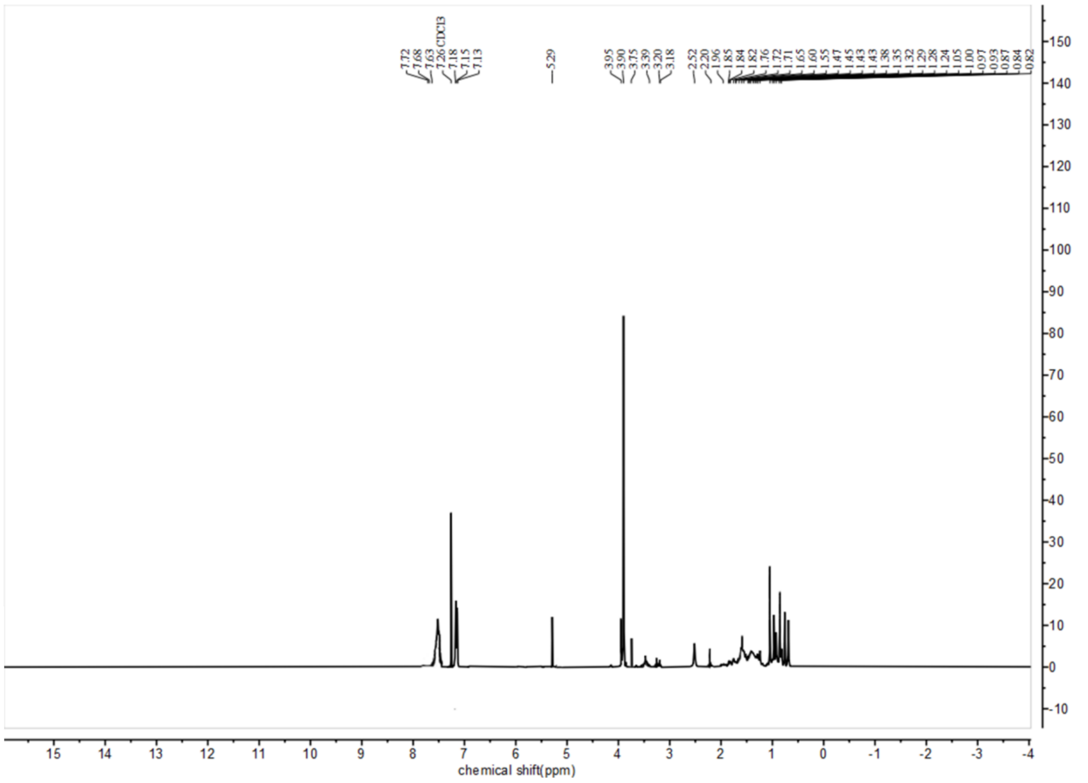

Figures S38. ^{13}C *and* ^{1}H *NMR of compound* ***38***.

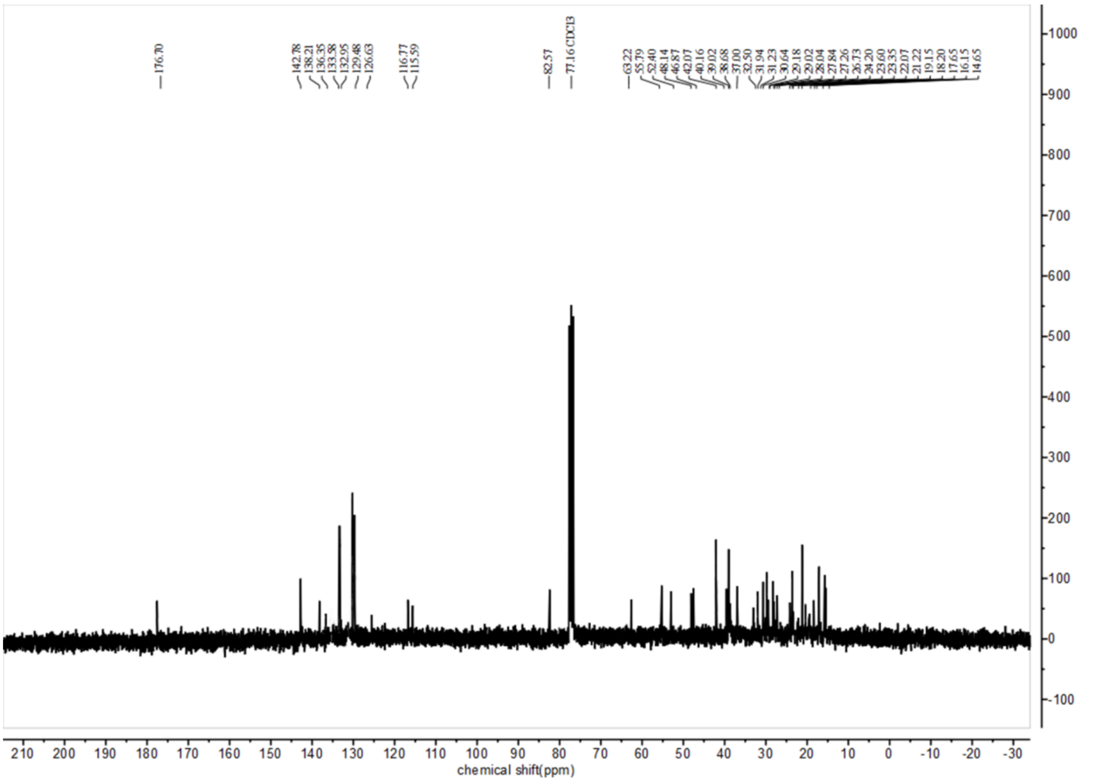

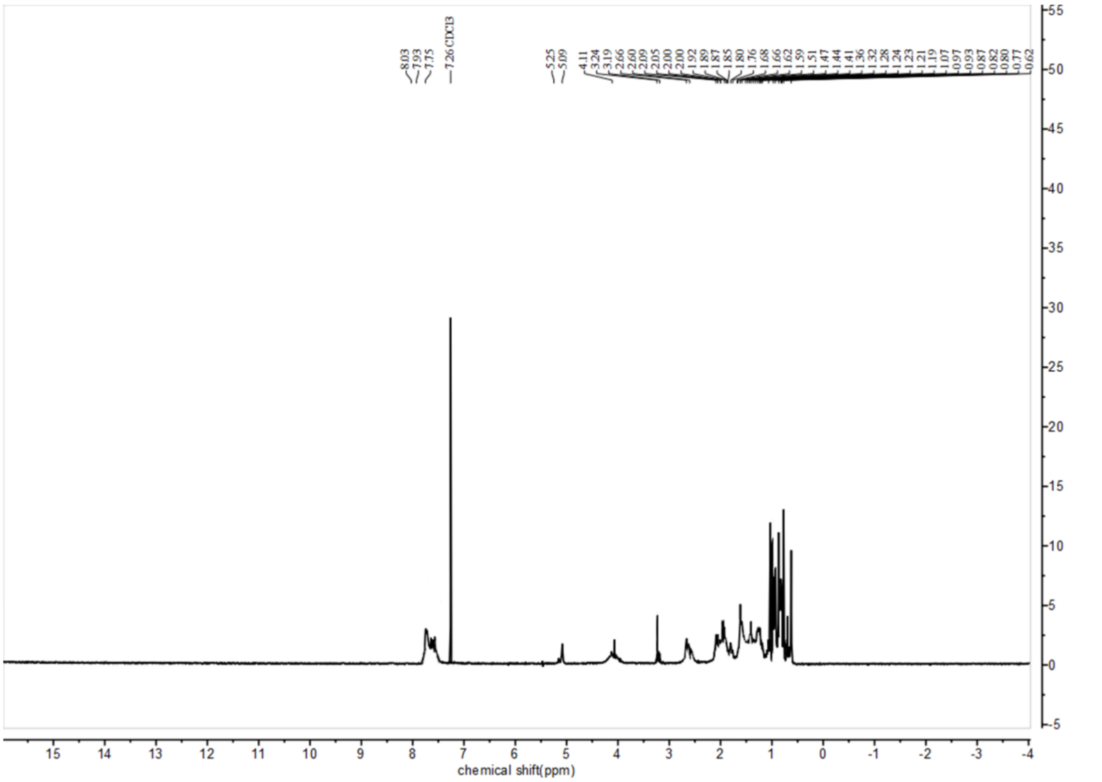

Figures S39. *^{13}C and ^{1}H NMR of compound* ***39***.

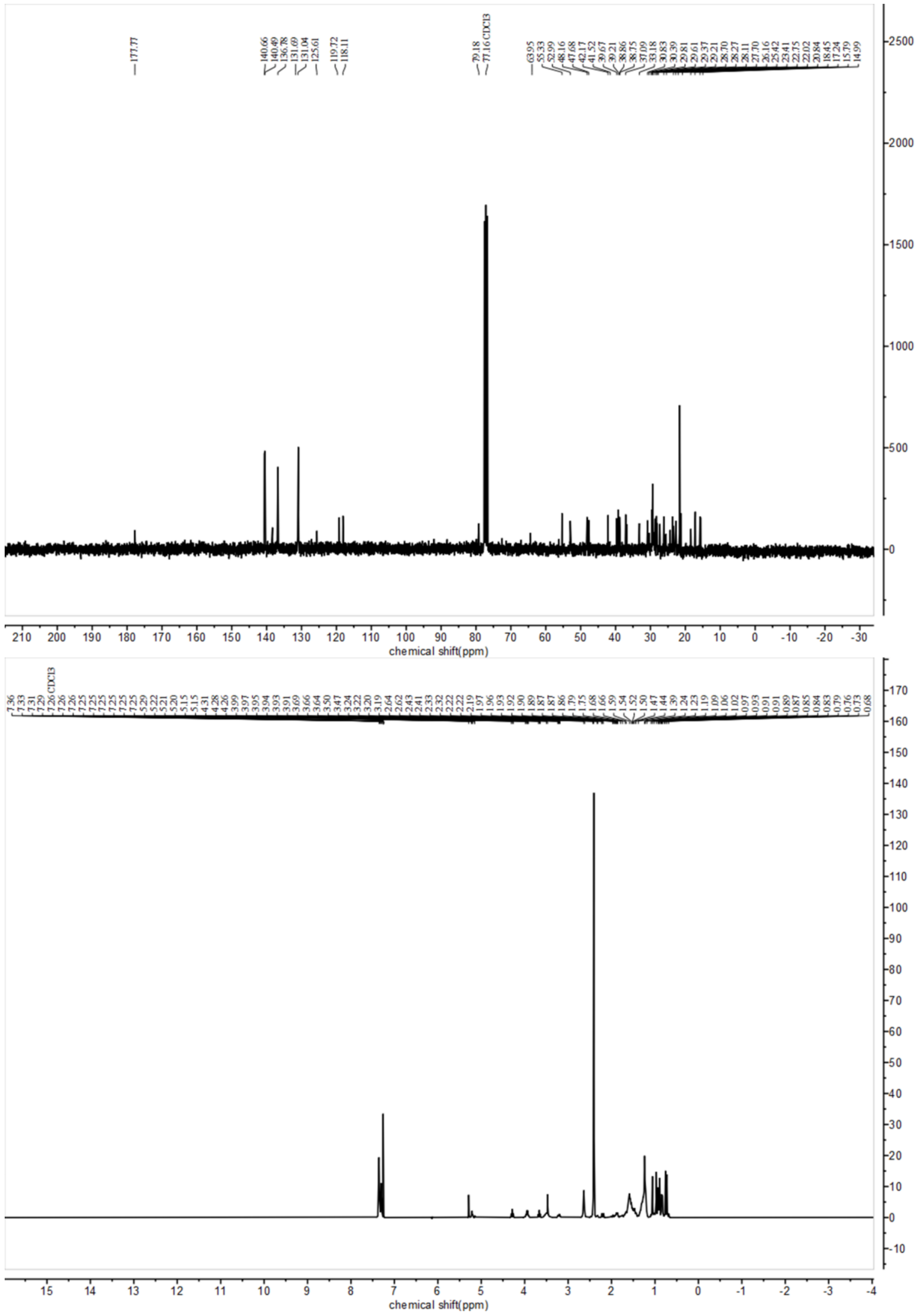

Figures S40. ^{13}C *and* ^{1}H *NMR of compound* ***40***.

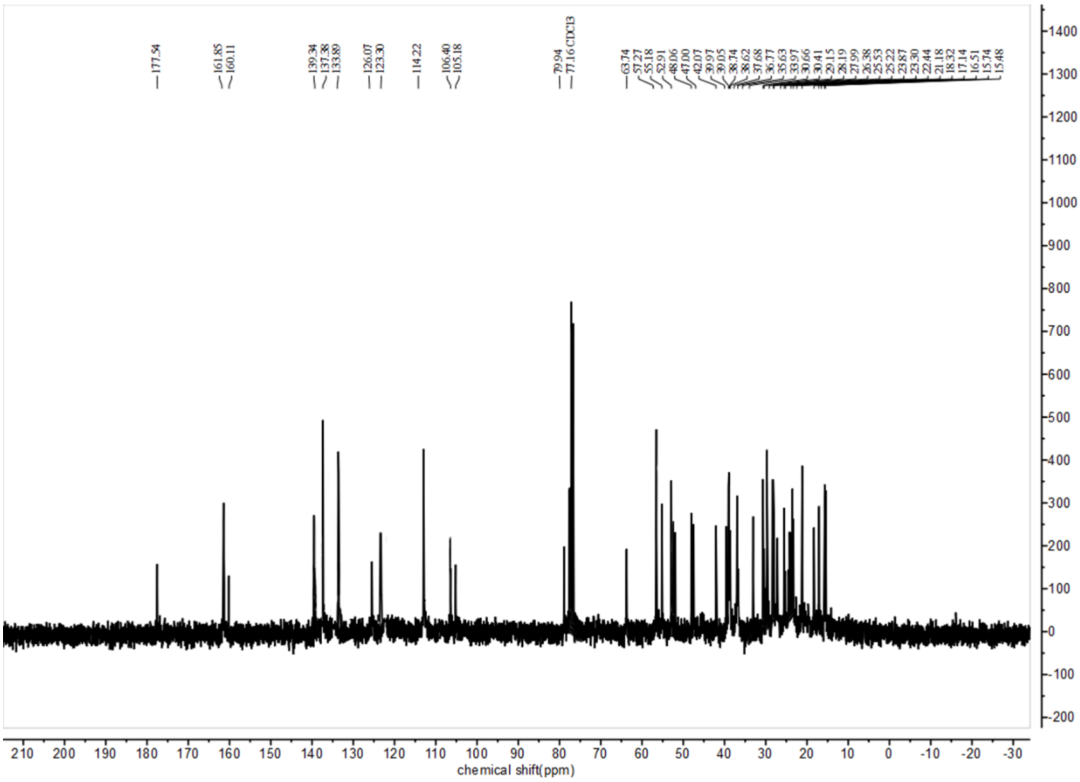

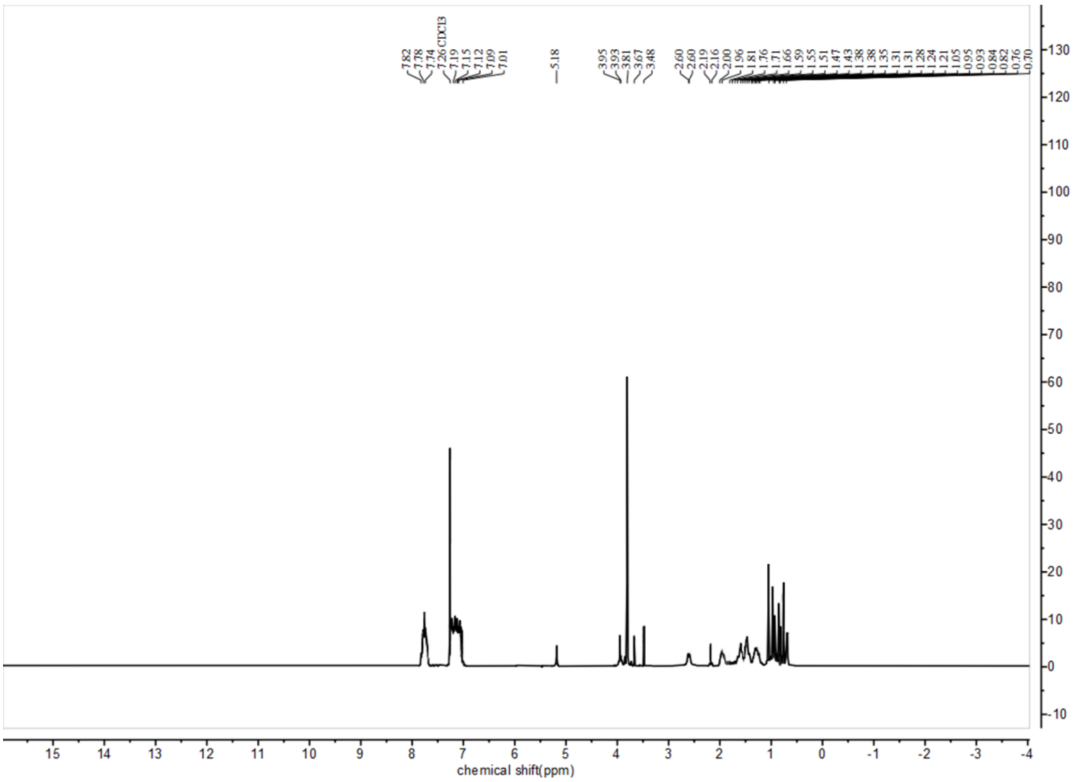

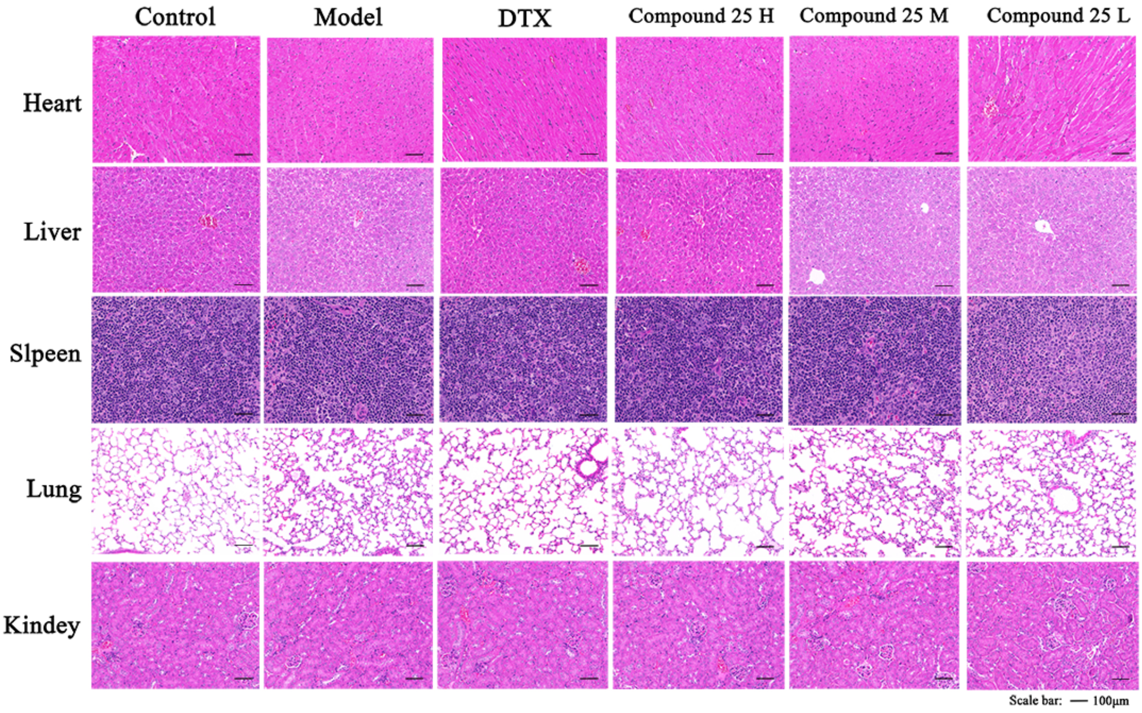

Figures S41. Compound 25 shows no obvious toxic effects on the heart, liver, spleen, lung, and kidney of mice. Data are presented as mean ± SD (n = 3) (Magnification × 100).

Western blot：

BCL2：

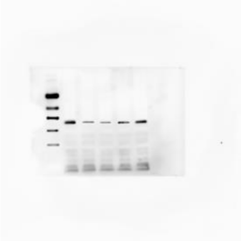

RAC1：

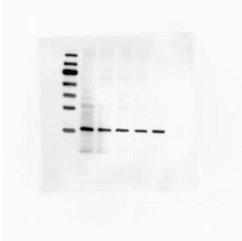

BAX：

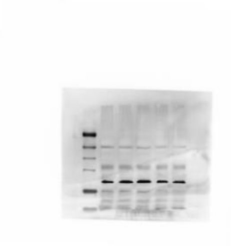

Cleaved-caspased-3：

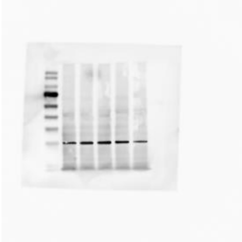

Cleaved-caspased-9：

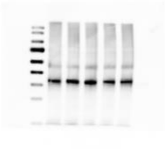

Cyt c：

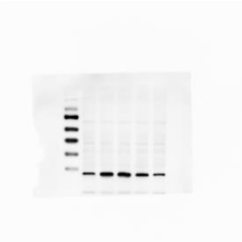

F-actin：

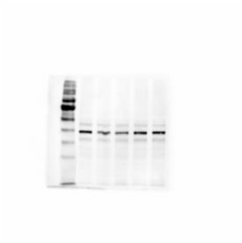

G-actin：

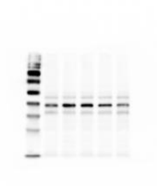

FGFR1：

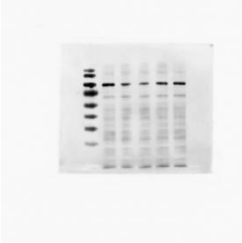

p-FGFR1：

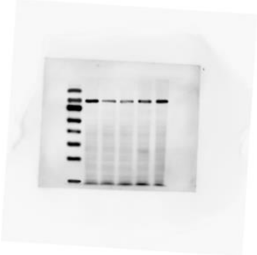

GSN：

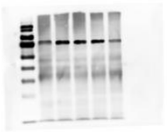

PIP4K2：

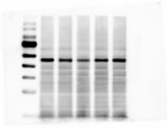

GAPDH：

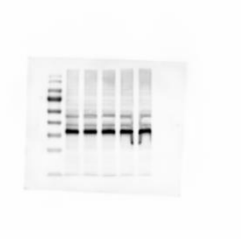

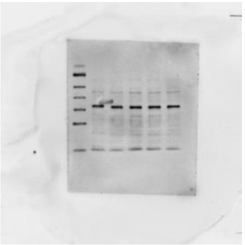

Supplement: Supplementary file 1 [file pharmaceuticals-19-00726-s001.zip › pharmaceuticals-4269319-supplementary.pdf]
